# Aspirin reprogrammes colorectal cancer cell metabolism and sensitises to glutaminase inhibition

**DOI:** 10.1186/s40170-023-00318-y

**Published:** 2023-10-19

**Authors:** Amy K. Holt, Arafath K. Najumudeen, Tracey J. Collard, Hao Li, Laura M. Millett, Ashley J. Hoskin, Danny N. Legge, Eleanor M. H. Mortensson, Dustin J. Flanagan, Nicholas Jones, Madhu Kollareddy, Penny Timms, Matthew D. Hitchings, James Cronin, Owen J. Sansom, Ann C. Williams, Emma E. Vincent

**Affiliations:** 1 School of Cellular and Molecular Medicine, Biomedical Sciences Building, University of Bristol, Bristol, BS8 1TW UK; 2 Cancer Research UK Beatson Institute, Glasgow, G61 1BD UK; 3 Institute of Biotechnology, HiLIFE, University of Helsinki, Helsinki, Finland; 4 School of Translational Health Sciences, Dorothy Hodgkin Building, University of Bristol, Bristol, BS1 3NY UK; 5 Institute of Life Science, Swansea University Medical School, Swansea University, Swansea, SA2 8PP UK; 6 Institute of Cancer Sciences, University of Glasgow, Glasgow, G61 1QH UK; 7 MRC Integrative Epidemiology Unit, Oakfield House, University of Bristol, Bristol, BS8 2BN UK

**Keywords:** Colorectal cancer, Aspirin, Metabolism, Metabolic reprogramming, CB-839, Glutaminase

## Abstract

**Background:**

To support proliferation and survival within a challenging microenvironment, cancer cells must reprogramme their metabolism. As such, targeting cancer cell metabolism is a promising therapeutic avenue. However, identifying tractable nodes of metabolic vulnerability in cancer cells is challenging due to their metabolic plasticity. Identification of effective treatment combinations to counter this is an active area of research. Aspirin has a well-established role in cancer prevention, particularly in colorectal cancer (CRC), although the mechanisms are not fully understood.

**Methods:**

We generated a model to investigate the impact of long-term (52 weeks) aspirin exposure on CRC cells, which has allowed us comprehensively characterise the metabolic impact of long-term aspirin exposure (2–4mM for 52 weeks) using proteomics, Seahorse Extracellular Flux Analysis and Stable Isotope Labelling (SIL). Using this information, we were able to identify nodes of metabolic vulnerability for further targeting, investigating the impact of combining aspirin with metabolic inhibitors in vitro and in vivo.

**Results:**

We show that aspirin regulates several enzymes and transporters of central carbon metabolism and results in a reduction in glutaminolysis and a concomitant increase in glucose metabolism, demonstrating reprogramming of nutrient utilisation. We show that aspirin causes likely compensatory changes that render the cells sensitive to the glutaminase 1 (GLS1) inhibitor—CB-839. Of note given the clinical interest, treatment with CB-839 alone had little effect on CRC cell growth or survival. However, in combination with aspirin, CB-839 inhibited CRC cell proliferation and induced apoptosis in vitro and, importantly, reduced crypt proliferation in *Apc*^*fl/fl*^ mice in vivo*.*

**Conclusions:**

Together, these results show that aspirin leads to significant metabolic reprogramming in colorectal cancer cells and raises the possibility that aspirin could significantly increase the efficacy of metabolic cancer therapies in CRC.

**Supplementary Information:**

The online version contains supplementary material available at 10.1186/s40170-023-00318-y.

## Background

Metabolic reprogramming is a defining feature of cancer cells [1] and is essential to meet both the energetic and biosynthetic requirements of chronic proliferation. Although the specific nature of cancer cell metabolism depends on several factors including tissue of origin and mutational status, often common features include increased aerobic glycolysis (known as the Warburg effect) and glutamine utilisation [2, 3].

Colorectal cancer (CRC) is the second most common cause of cancer-related death in the UK, and incidence is increasing, particularly in younger patient populations [4, 5], highlighting the need for improved therapies. Metabolic reprogramming supports CRC initiation and progression, and it is well established that it is a driver rather than a passive outcome of tumourigenesis. Indeed, many key oncogenic pathways in CRC have been shown to directly control metabolism, including Wnt, PI3K and p53 signalling [6, 7]. As such, cancer metabolism is an attractive target for novel therapies. However, many challenges remain in developing metabolic anti-cancer therapies, such as the large overlap between the metabolic programme favoured by cancer cells and normal proliferating cells, resulting in a small therapeutic window and the increased likelihood of toxicity [8]. There is also the challenge of overcoming metabolic plasticity; cancer cells are well suited to adapting their metabolism to meet environmental constraints, such as hypoxia and hypoglycaemia, and the contrasting conditions of the bloodstream and metastatic sites [9, 10]. As a result, when targeted with singular metabolic interventions, cancer cells often rewire their metabolism to enable continued proliferation.

Aspirin is a widely used non-steroidal anti-inflammatory drug (NSAID) prescribed for the prevention of cardiovascular events in high-risk patients. A growing body of epidemiological evidence suggests that aspirin reduces cancer incidence, in particular CRC, as well as potentially slowing disease progression [11, 12] and increasing patient survival [13]. The US Preventative Services Task Force recommends daily aspirin for CRC prevention in 50–69-year-olds with an increased risk of cardiovascular disease and no increased risk of bleeding [14]. Furthermore, the National Institute for Health and Care Excellence (NICE) guidelines now recommend daily aspirin for the prevention of CRC in patients with Lynch syndrome [15].

Aspirin is a pleiotropic drug; its actions at the cellular level are not fully understood, particularly with regard to its role in cancer prevention. Increased knowledge of aspirin’s cellular mechanisms could enhance its efficacy, including identification of optimal timing and dose, and those individuals most likely to benefit from taking regular aspirin [16, 17].

Epidemiological data suggest that the effect of aspirin on CRC incidence and progression is affected by the length of time for which aspirin is taken [11, 18]. While the effects of aspirin have been extensively studied in vitro and in vivo, long-term exposure has not been modelled before in cell lines. Therefore, in this study, we investigated the impact of long-term (52 weeks) aspirin exposure on CRC cells, with the aim of identifying novel mechanisms of action. Detailed proteomic and metabolomic analysis revealed altered metabolism and nutrient utilisation with aspirin exposure.

Several key enzymes involved in central carbon metabolism were identified as being regulated by aspirin, including pyruvate carboxylase (PC), pyruvate dehydrogenase kinase 1 (PDK1) and glutaminase 1 (GLS1). Although aspirin alone did not impact the ability of the cells to produce ATP, it does inhibit net glutaminolysis, despite inducing a (likely compensatory) increase in GLS1 expression. Importantly, although the GLS1 inhibitor CB-839 alone had little effect on CRC cell survival, aspirin renders colorectal cells sensitive to the drug both in vitro and in vivo. In addition, reduced glutaminolysis upon aspirin exposure leads to a concomitant and likely compensatory increase in glucose utilisation in the tricarboxylic acid (TCA) cycle, leaving cells sensitive to the mitochondrial pyruvate carrier 1 (MPC1) inhibitor, UK-5099. In summary, we demonstrate that aspirin causes metabolic rewiring in colorectal cancer cells providing therapeutic opportunities to sensitise colorectal cancer to existing metabolic cancer therapies currently under clinical investigation.

## Methods

### Cell lines and culture

The human colorectal carcinoma-derived cell lines; SW620 and LS174T were obtained from the American Type Culture Collection (ATCC, Maryland, USA), and HCA7 was a kind gift from Dr. Susan Kirkland, Imperial College, London. All cell lines were routinely tested for mycoplasma contamination using MycoAlert PLUS mycoplasma detection kit (Lonza, MD, USA) and molecularly characterised using an “in house” panel of cellular and molecular markers to check that cell lines have not been cross contaminated (every 3–6 months). Stocks were securely catalogued and stored; passage numbers strictly adhered to prevent phenotypic drift. All cell lines were cultured in Dulbecco’s modified Eagle medium (DMEM) (Sigma-Aldrich, Merck, KGAa) with added 10% foetal bovine serum (FBS) (Sigma-Aldrich, Merck, KGaA), 2 mM glutamine (Gibco, ThermoFisher Scientific Inc.), 100 units/ml penicillin and 100 units/ml streptomycin (Gibco, ThermoFisher Scientific Inc). For stock purposes, cells were maintained in 25cm^2^ tissue culture (T25) flasks (Corning, NY, USA) and incubated at 37℃ in dry incubators maintained at 5% CO_2_. Cell media were changed every 3–4 days. Experiments were performed in triplicate independently with distinct passages of cells, unless otherwise stated.

### Treatments

#### Long-term aspirin

For the long-term aspirin-treated cells, a 20-mM stock solution of aspirin (Sigma, Merck KGaA, Darmstadt, Germany) was created by adding 3.6 mg/ml aspirin to 10% DMEM, and fresh aspirin was made up immediately prior to use. Aspirin concentration in the growth media was maintained continuously for ~ 52 weeks. Passage frequency and ratio were adjusted to maintain confluency in aspirin-treated cells.

#### CB-839, inhibitor 968 and UK-5099

Stock of CB-839 (Sigma-Aldrich, Merck, KGaA), inhibitor 968 (Sigma-Aldrich, Merck, KGaA) and UK-5099 (Sigma Aldrich, Merck, KGaA) were made in dimethyl sulfoxide (DMSO). DMSO concentration was maintained consistently in all treatment concentrations and vehicle control.

### Proliferation assays

#### Crystal violet staining

To measure the proliferation of cells treated with aspirin in combination with either CB-839 or UK-5099, cells were seeded into 96 well plates (Corning, NY, USA) (20,000 cells per well in all conditions except HCA7 cells treated with 4 mM aspirin, where 40,000 cells per well were seeded) in normal growth medium and incubated for 24 h, with 3–4 technical replicate wells per treatment condition. Cells were then treated with media containing drug treatments (or vehicle control) and incubated for a further 72 h. Plates were then fixed with 4% PFA for 15 min, then stained with 0.5% crystal violet solution (Sigma-Aldrich, Merck KGaA), before solubilisation in 2% SDS, and subsequent OD595 measurements were obtained using an iMark microplate reader (Bio-Rad, Laboratories, Inc.). The number of adherent cells was claculated by the confluence in each concentration of CB-839/UK-5099 at 72 h, relative to the same concentration of aspirin in control conditions, in order to compare the effect of the drugs between different aspirin treatments.

For experiments using Human Plasma-Like Medium (HPLM – Gibco, ThermoFisher Scientific, Inc.), HPLM was supplemented with 10% dialysed FBS (dFBS) and experiments were performed as above. Cells were incubated in 10% dFBS HPLM for at least 48 h prior to the start of the treatment, to allow for metabolic adaptation. During the experiment, media was changed every 24 h (unlike experiments in DMEM where the same media was left for the full 72 h of treatment), in order to avoid depletion of the low levels of nutrients.

#### IncuCyte

To simultaneously measure cell proliferation and apoptosis upon treatment with aspirin in combination with CB-839, a IncuCyte ZOOM live cell imaging system was used. Cells were seeded in 96 well plates (20,000 cells per well) and incubated for 24 h, with 3–4 wells per treatment condition (technical replicates). Cells were then treated with treatment-containing media (or vehicle control) and placed in the IncuCyte system. The percentage of confluence was measured every 4 h for the total time indicated on the graphs. The IncuCyte system took four different image fields per well. At the time of treatment, the cells were also treated with 2-µM CellEvent caspase-3/7 green detection reagent (C10423; Invitrogen, ThermoFisher Scientific, Inc), which was used to measure apoptosis. Green fluorescent cells, indicating active caspase-3/7 and apoptosis, were measured by the IncuCyte system as green object count (1/mm^2^). For each individual well, the green object count was normalised to the confluence at each timepoint, and results were expressed as relative apoptosis. The same method was performed using SW620 cells treated with 2 µM ABT-737 compared to a vehicle control prior to the start of the assay, as a positive control for apoptosis in order to validate this assay and confirm the detection of apoptotic cells.

### Proteomic analysis

#### TMT labelling and high pH reversed-phase chromatography

Following 52 weeks of aspirin treatment to develop long-term treated cell lines, cells were seeded in T25 flasks, and following maintenance in aspirin for a further 72 h whole-cell protein lysates were collected. Lysates were collected as described previously [19]. Protein concentrations were ascertained, and samples were adjusted to 2 mg/mL. One hundred micrograms of each sample was digested with trypsin overnight at 37℃, labelled with tandem mass tag (TMT) ten plex reagents according to the manufacturer’s protocol (ThermoFisher Scientific, Inc.) and the labelled samples pooled.

An aliquot of the pooled sample was evaporated to dryness, resuspended in 5% formic acid and then desalted using a SepPak cartridge according to the manufacturer’s instructions (Waters, Milford, Massachusetts, USA). Eluate from the SepPak cartridge was again evaporated to dryness and resuspended in buffer A (20-mM ammonium hydroxide, pH 10) prior to fractionation by high pH reversed-phase chromatography using an ultimate 3000 liquid chromatography system (ThermoFisher Scientific, Inc.). In brief, the sample was loaded onto a XBridge BEH C18 column (130 Å, 3.5 µm, 2.1 mm X 150 mm, Waters, Milford, Massachusetts, USA) in buffer A and peptides eluted with an increasing gradient of buffer B (20-mM ammonium hydroxide in acetonitrile, pH 10) from 0 to 95% over 60 min. The resulting fractions (15 in total) were evaporated to dryness and resuspended in 1% formic acid prior to analysis by nano-LC MSMS using an Orbitrap Fusion Tribrid mass spectrometer (ThermoFisher Scientific, Inc.).

#### Nano-LC mass spectrometry

High pH RP fractions were further fractionated using an ultimate 3000 nano-LC system in line with an Orbitrap Fusion Tribrid mass spectrometer (ThermoFisher Scientific, Inc.). In brief, peptides in 1% (v/v) formic acid were injected onto an Acclaim PepMap C18 nano-trap column (ThermoFisher Scientific, Inc.). After washing with 0.5% (v/v), acetonitrile 0.1% (v/v) formic acid peptides were resolved on a 250 mm × 75 μm Acclaim PepMap C18 reverse-phase analytical column (ThermoFisher Scientific, Inc.) over a 150-min organic gradient, using 7 gradient segments (1–6% solvent B over 1 min, 6–15% B over 58 min, 15–32% B over 58 min, 32–40%B over 5 min, 40–90%B over 1 min, held at 90%B for 6 min and then reduced to 1%B over 1 min) with a flow rate of 300 nl min^−1^. Solvent A was 0.1% formic acid, and solvent B was aqueous 80% acetonitrile in 0.1% formic acid. Peptides were ionised by nano-electrospray ionisation at 2.0 kV using a stainless-steel emitter with an internal diameter of 30 μm (Thermo Scientific) and a capillary temperature of 275℃.

All spectra were acquired using an Orbitrap Fusion Tribrid mass spectrometer controlled by Xcalibur 2.1 software (Thermo Scientific) and operated in data-dependent acquisition mode using an SPS-MS3 workflow. FTMS1 spectra were collected at a resolution of 120,000, with an automatic gain control (AGC) target of 200,000 and a max injection time of 50 ms. Precursors were filtered with an intensity threshold of 5000, according to charge state (to include charge states 2–7) and with monoisotopic peak determination set to peptide. Previously interrogated precursors were excluded using a dynamic window (60 s ± 10 ppm). The MS2 precursors were isolated with a quadrupole isolation window of 1.2 m/z. ITMS2 spectra were collected with an AGC target of 10,000, max injection time of 70 ms and CID collision energy of 35%.

For FTMS3 analysis, the Orbitrap was operated at 50,000 resolution with an AGC target of 50,000 and a max injection time of 105 ms. Precursors were fragmented by high energy collision dissociation (HCD) at a normalised collision energy of 60% to ensure maximal TMT reporter ion yield. Synchronous precursor selection (SPS) was enabled to include up to 5 MS2 fragment ions in the FTMS3 scan.

#### Data analysis

The raw data files (supplied in Supplementary Data File S2) were processed and quantified using Proteome Discoverer software v2.1 (ThermoFisher Scientific, Inc.) and searched against the UniProt Human database (downloaded September 2017; 140,000 sequences) using the SEQUEST HT algorithm. Peptide precursor mass tolerance was set at 10 ppm, and MS/MS tolerance was set at 0.6 Da. Search criteria included oxidation of methionine (+ 15.995 Da), acetylation of the protein N terminus (+ 42.011 Da) and methionine loss plus acetylation of the protein N terminus (− 89.03 Da) as variable modifications and carbamidomethylation of cysteine (+ 57.021 Da) and the addition of the TMT mass tag (+ 229.163 Da) to peptide N termini and lysine as fixed modifications. Searches were performed with full tryptic digestion, and a maximum of two missed cleavages were allowed. The reverse database search option was enabled, and all data was filtered to satisfy a false discovery rate (FDR) of 5%.

#### Protein abundance processing

Protein groupings were determined by PD2.1; however, the master protein selection was improved with an in-house script. This enables us to infer biological trends more effectively in the dataset without any loss in the quality of identification or quantification. The MS data were searched against the human Uniprot database retrieved on October 2, 2019, and updated with additional annotation information on April 21, 2020.

The protein abundances were normalised within each sample to the total peptide amount, then Log2 transformed to bring them closer to a normal distribution.

#### Statistics

Statistical significance was then determined using Welch’s *T* tests between the conditions of interest. The *p* values were FDR-corrected using the Benjamini–Hochberg method.

#### QIAGEN Ingenuity Pathway Analysis (QIAGEN IPA)

Data were analysed using ingenuity pathway analysis. Proteins from the dataset that met the cutoff of *p* < 0.05 were considered for the analysis and compared to a reference set consisting of the full list of proteins identified in the experiment. A right-tailed Fisher’s exact test was used to calculate a *p* value determining the probability that the association between the genes in the dataset and the pathways/upstream regulators/functions was by chance alone, and the predicted and observed regulation patterns of the proteins were used to predict an activation *z* score.

#### Overrepresentation analysis

Overrepresentation analysis was performed using Webgestalt (www.webgestalt.org), using the functional databases Geneontology, and pathways (KEGG—Kyoto Encyclopedia of Genes and Genomes). Gene symbols were entered for proteins that showed signification regulation (*p* < 0.05, fold change > 1.4 or < 0.71), in both 2-mM and 4-mM long-term aspirin compared to control.

### SDS-PAGE and immunoblotting

Cell lysates were prepared and subjected to western analysis as described previously [19] using antibodies to the following: α-tubulin (T9026, Sigma-Aldrich, Merck, Inc.), ATF4 (11,815, Cell Signaling Technology Inc. (CST)), ASCT2 (5345, CST), GLS1 (88,964, CST), GPT2 (16,757–1-AP, ProteinTech, Group, Inc.), LAT1 (5347, CST), PC (ab126707, Abcam, Cambridge, UK), PDK1 (3820, CST), HK1 (2024, CST), and GLUT1 (21,829–1-AP20, Proteintech, Group, Inc.). All were used at 1:1000 dilution, except for α-tubulin which was used at 1:10,000 dilution. The density of bands detected by immunoblotting was measured using ImageJ. In order to compare data from independent experiments, protein expression changes were calculated as relative changes from the control conditions in each replicate. Results from at least three independent experiments were analysed with this method.

### Quantitative reverse transcriptase-PCR (qPCR)

Total RNA was extracted using Tri-Reagent (Sigma-Aldrich) as per manufacturer’s instruction an RNAeasy mini kit with an ON-column DNA digest step (Qiagen, Limberg, Netherlands) was used according to the manufacturer’s instructions to clean up the RNA. RNA concentration and purity were measured using a NanoDrop™ spectrophotometer (ThermoFisher Scientific Inc.). Synthesis of cDNA and qRT-PCR were performed as previously described [20] using the following primers (all from Qiagen, Limberg, Netherlands): *ATF4* (QT00074466), *GPT2* (QT00066381), *GLS1* (QT00019397), *PC* (QT01005592), *PDK1* (QT00069636), *SLC7A11* (QT00002674) and *SLC7A5* (QT00089145). Gene expression was normalised to the housekeeping genes *TBP* (cat. no. QT00000721) or *HPRT* (QT00059066), both from Qiagen.

### Extracellular flux analysis

Extracellular flux analysis was carried out using the XFp Seahorse Extracellular Flux Analyzer (Agilent), according to the manufacturer’s protocol. Long-term aspirin-treated SW620 cells (60,000 cells) were seeded onto a Cell-Tak (354,240, Corning, NY, USA)-coated microplate (2–3 technical replicate wells per condition) and centrifuged at 200 g for 1 min (no brake), allowing for immediate adhesion. Cells were seeded in Seahorse XF assay media (Agilent, CA, USA) supplemented with 10 mM glucose, 2 mM glutamine and 1 mM pyruvate (Agilent, CA, USA). Corresponding OCR/ECAR (oxygen consumption rate/extracellular acidification rate) changes were monitored for the duration of the experiment. Wells had subsequent injections of oligomycin (2 µM), FCCP (2 µM), antimycin A (1 µM) and rotenone (1 µM) and monensin (20 µM) (in order to determine the maximal glycolytic rate, as shown by Mookerjee et al. [21, 22]) all from Sigma-Aldrich. Data were acquired using the Seahorse Wave software v2.6 (Agilent, CA, USA). The experiment was performed independently in triplicate.

### Stable isotope tracer analysis

For stable isotope labelling (SIL) experiments, cells were cultured with U-[^13^C]-Glc or U-[^13^C]-Q (Cambridge Isotopes Laboratories, Inc.) for the indicated time points. ^13^C-labelled nutrients were added to glucose, glutamine-free DMEM, supplemented with 10% dFBS, 100 units/ml penicillin and 100 units/ml streptomycin, 10 mM glucose and 2 mM glutamine (^13^C-labelled or unlabelled as appropriate). Cellular metabolites were extracted and analysed by gas chromatography-mass spectrometry (GC–MS) using protocols described previously [23–25]. Metabolite extracts were derived using *N*-(tert-butyldimethylsilyl)-*N*-methyltrifluoroacetamide (MTBSTFA) as described previously [26]. D-myristic acid (750 ng/sample) was added as an internal standard to metabolite extracts, and metabolite abundance was expressed relative to the internal standard and normalised to cell number. Mass isotopomer distribution was determined using a custom algorithm developed at McGill University [25]. The experiment was performed with three different flasks of cells from the same passage number per condition. Raw data are supplied in Supplementary Data File S1.

### In vivo experiments

All in vivo experiments were carried out in accordance with the UK Home Office regulations (under project licences: 70/8646 and PP3908577) and by adhering to the ARRIVE guidelines with approval from the Animal Welfare and Ethical Review Board of the University of Glasgow. Mice were housed under a 12-h light–dark cycle, at constant temperature (19–23℃) and humidity (55 ± 10%). Standard diet and water were available ad libitum. The majority of the work was performed in the C57BL/6J background. The following alleles were used in this study: *VillinCreER* [27], *Apc*^*fl*^ [28]. Full intestinal recombination was obtained by two intraperitoneal injections of 2mg tamoxifen, and tissues were harvested 4 days post induction. For drug studies in vivo*,* Villin-Cre^ERT2^*Apc*^*fl/fl*^ mice were treated with CB-839 (200mg/kg in 25% (w/v) hydroxypropyl-β-cyclodextrin in 10mm citrate at pH 2.0) or vehicle from day 1 post i.p. tamoxifen administration. For aspirin and combination treatments, mice received aspirin (2.6mg/ml in drinking water) 2 days prior to tamoxifen administration and remained on aspirin till the end of the study. Animals were injected with BrdU (i.p.) 2 h prior to sampling tissues.

### Immunohistochemistry (IHC)

Mouse intestines were flushed with water, cut open longitudinally, pinned out onto silicone plates and fixed in 10% neutral buffered formalin overnight at 4℃. Fixed tissue was rolled from the proximal to distal end into Swiss-rolls and processed for paraffin embedding. Tissue blocks were cut into 5μm sections and stained with haematoxylin and eosin (H&E). IHC was performed on formalin-fixed intestinal sections according to standard staining protocols. The primary antibody used was against BrdU (1:150, BD Biosciences, #347,580), and representative images are shown.

### Statistical analysis

Data were presented and statistical analysis was performed using GraphPad Prism 9. Statistical tests were performed as stated, and significance was expressed as *p < 0.05, **p < 0.01, ***p < 0.001, and ****p < 0.0001. Results are expressed as mean values with standard error of the mean (SEM) where independent experiments are compared and with standard deviation (SD) where technical replicates are compared. Here, technical replicates refer to separate wells or flasks of cells that are from the same original passage of cells, seeded and treated at the same time. Independent experiments refer to separate passages of cells that were seeded at different times.

## Results

### Long-term aspirin exposure regulates expression of metabolic pathway genes in CRC cells

**Fig. 1:**
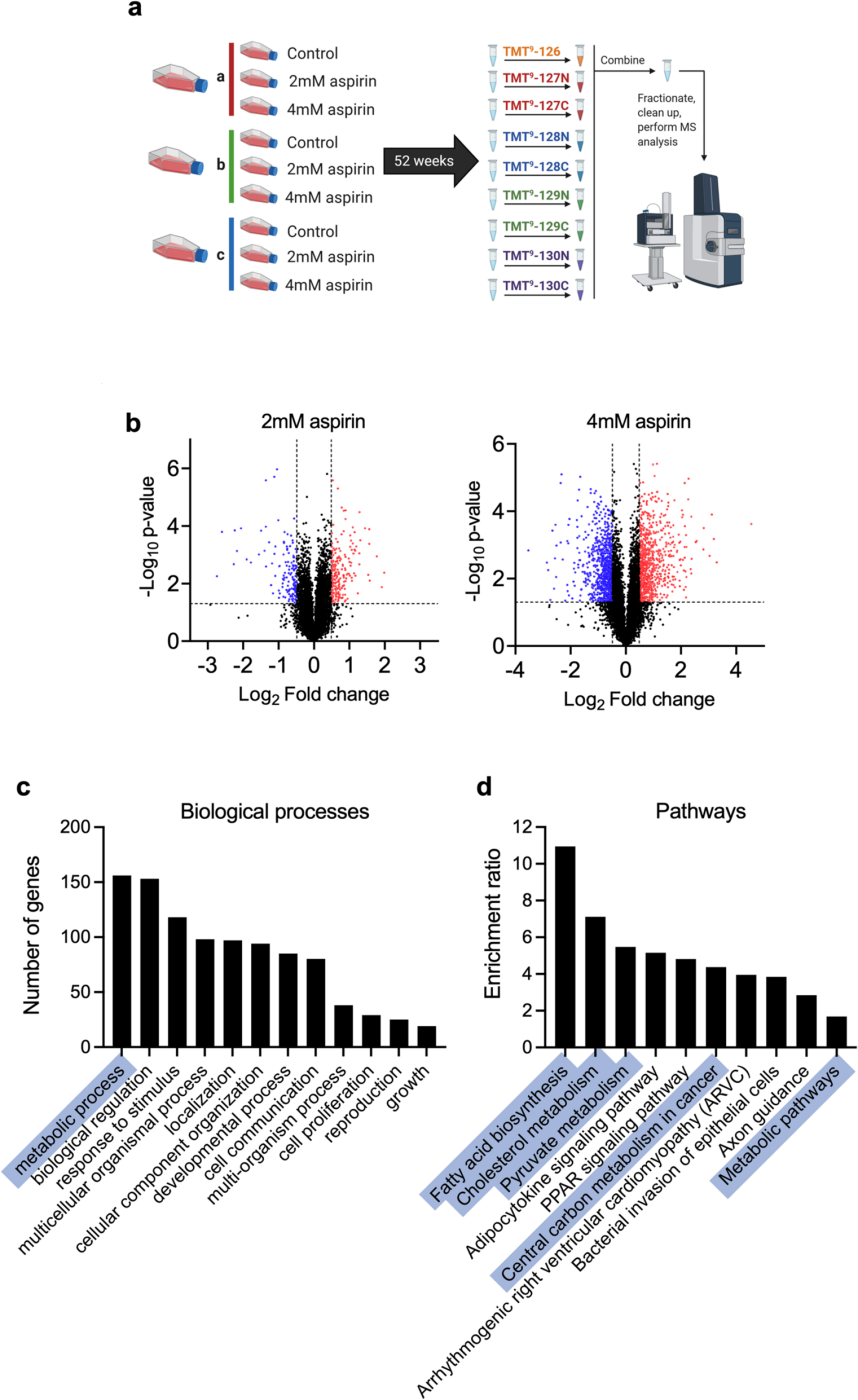
Long-term aspirin treatment regulates cellular metabolism in CRC cells. **a** Experimental design for proteomic analysis of long-term aspirin-treated SW620 cells (each flask represents one independent experiment) (created with BioRender.com). **b** Volcano plots showing protein expression changes with long-term 2-mM or 4-mM aspirin treatment compared to control. Each point represents one protein. Thresholds for proteins of interest were *p* < 0.05, fold change > 1.4 or < 0.71), in both 2mM and 4mM conditions (downregulated proteins in blue and upregulated in red). **c** Number of genes in GO biological processes categories, of all proteins of interest in both 2mM and 4mM aspirin. **d** Overrepresentation analysis of proteins of interest in both 2mM and 4mM aspirin, using the KEGG pathways database

To explore the consequences of long-term aspirin exposure on SW620 colorectal cancer cells, we performed proteomic analysis to compare protein expression in cells treated for 52 weeks in continuous culture with either 2mM or 4mM aspirin to untreated controls (experimental design shown in Fig. 1a). Two hundred sixty-five proteins were significantly differentially regulated in cells treated with both 2mM and 4mM aspirin compared to untreated controls (*p* < 0.05, fold change > 1.4 or < 0.71) [29] (Fig. 1b). Analysis of these proteins using Webgestalt highlighted the “metabolic process” as having the highest number of genes in the gene ontology (GO) biological processes (Fig. 1c). Overrepresentation analysis using the KEGG pathway database highlighted a high enrichment ratio in “metabolic pathways” and “central carbon metabolism in cancer”, as well as some specific metabolic pathways including “pyruvate metabolism”, “cholesterol metabolism” and “fatty acid biosynthesis” (Fig. 1d). These results suggest that long-term aspirin exposure might rewire cellular metabolic pathways in CRC cells.

### Aspirin exposure reprogrammes nutrient utilisation in CRC cells

**Fig. 2:**
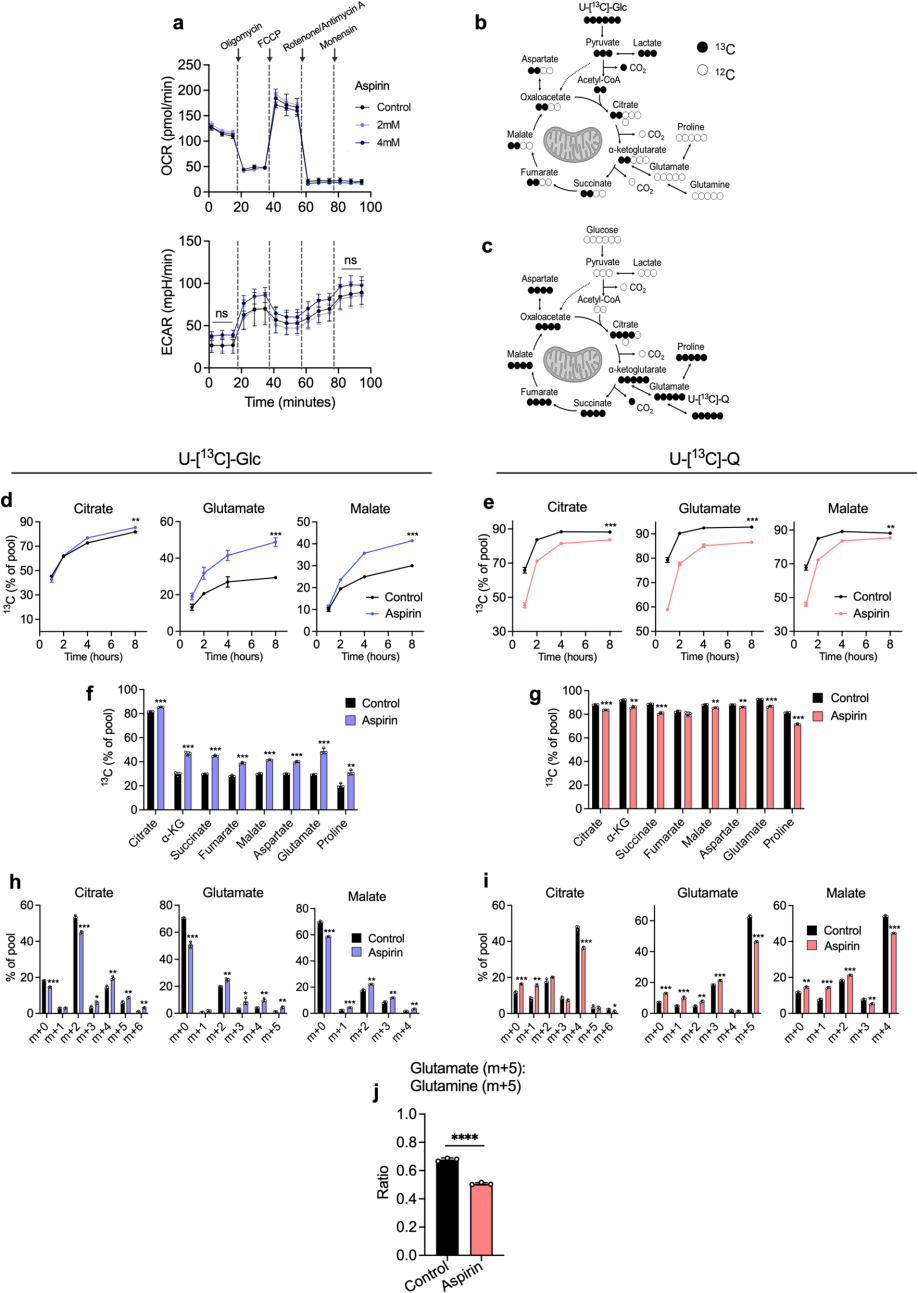
Long-term aspirin treatment reprogrammes nutrient utilisation in CRC cells. **a** Extracellular flux analysis of long-term (52 weeks) 2-mM and 4-mM aspirin-treated SW620 cells compared to controls. Error bars represent SEM (*n* = 3 independent experiments). ns = not significant (*p* > 0.05 following *t* tests at indicated time points). **b**, **c** Schematics of U-[^13^C]-Glc (**b**) and U-[^13^C]-Q (**c**) incorporation into TCA cycle metabolites and amino acids. Created with BioRender.com. **d**–**j** SIL data for long-term (52 weeks) 4-mM aspirin-treated SW620 cells compared to control cells. Error bars represent SD (*n* = 3 technical replicates). Asterisks refer to *p* values obtained from *t* tests at the 8-h time point (*p < 0.05, **p < 0.01, ***p < 0.001, ****p < 0.0001). **d**, **e** Proportion of ^13^C labelling in citrate, glutamate and malate from U-[^13^C]-Glc and U-[^13^C]-Q over time. **f**, **g** Proportion of ^13^C labelling in metabolite pools at 8 h from U-[^13^C]-Glc (f) and U-[^13^C]-Q (g). Asterisks indicate the adjusted *p* value obtained using multiple *t* tests. **h**, **i** Mass isotopomer distribution (MID) analysis at 8 h for U-[^13^C]-Glc (h) and U-[^13^C]-Q (i) labelling in citrate, glutamate and malate. Asterisks indicate the adjusted *p* value obtained using multiple *t* tests. **j** Ratio of m + 5 glutamateo m + 5 glutamine in long-term 4-mM aspirin-treated cells in comparison to control at 8 h from U-[^13^C]-Q

To determine whether aspirin exposure leads to a functional change in energy metabolism as predicted by the proteomic analysis, we next investigated the effect of long-term aspirin exposure on ATP (adenosine triphosphate) production from glycolysis and oxidative phosphorylation (oxphos) using a Seahorse Extracellular Flux Analyzer. Surprisingly, no changes were observed in either basal or maximal oxygen consumption rate (OCR) or extracellular acidification rate (ECAR), proxy measures of oxidative and glycolytic activity, respectively, with long-term aspirin treatment (Fig. 2a). This suggests that aspirin exposure has no net impact on ATP production in CRC cells and is therefore unlikely to explain the known effect of aspirin on cellular proliferation [30].

We next conducted stable isotope labelling (SIL) experiments to investigate whether aspirin altered the metabolic fate of glucose and glutamine, the most important carbon sources for proliferating cancer cells in culture. Long-term (52 weeks) aspirin-treated SW620 cells were incubated with either uniformly labelled ^13^C-glucose (U-[^13^C]-Glc) or glutamine (U-[^13^C]-Q) for up to 8 h in order to capture isotopic steady state [31]. The conventional metabolism of U-[^13^C]-Glc and U-[^13^C]-Q in tumour cells is illustrated in Fig. 2b, c. The majority of metabolites reach isotopic steady state within 8 h of incubation (shown for citrate, glutamate and malate), with the steady-state proportion of labelled metabolite (indicating labelled nutrient contribution) being altered by aspirin exposure (Fig. 2d, e). Incorporation of U-[^13^C]-Glc and U-[^13^C]-Q across TCA cycle metabolites at 8 h was increased and decreased respectively upon aspirin exposure (Fig. 2f, g). Similar results were observed in LS174T and HCA7 cells exposed to long-term aspirin (Supplementary Fig. 1).

Mass isotopomer distribution (MID) analysis of citrate, glutamate and malate shows a decrease in the unlabelled metabolites (m + 0) and an increase in the proportion of the glucose-labelled mass isotopomers with aspirin exposure (Fig. 2h). By contrast, an increase in unlabelled metabolites (m + 0) and a decrease in the proportion of the glutamine-labelled isotopomers was observed (Fig. 2i). These data suggest that glutaminolysis is inhibited by aspirin exposure, confirmed by analysis of the glutamate to glutamine m + 5 ratio (Fig. 2j).

These data demonstrate that despite there being no overall impact of long-term aspirin exposure on ATP production, it does cause metabolic reprogramming in three different CRC cell lines, reducing glutaminolysis and increasing glucose utilisation. Glucose and glutamine cooperate in fuelling the TCA cycle; a decrease in the entry of one nutrient can lead to a compensatory increase in the other [32, 33]. This suggests that the increased glucose utilisation may be a compensation mechanism for the reduction in glutaminolysis in the presence of aspirin in order to maintain carbon entry into the TCA cycle. As there was no overall effect on oxphos, this suggests the cells can maintain TCA cycle function in the presence of aspirin by increasing glucose utilisation. Taken together, these data highlight the metabolic plasticity of the cells, allowing them to minimise the impact on ATP production in the presence of aspirin.

### Aspirin regulates levels of proteins involved in central carbon metabolism

**Fig. 3:**
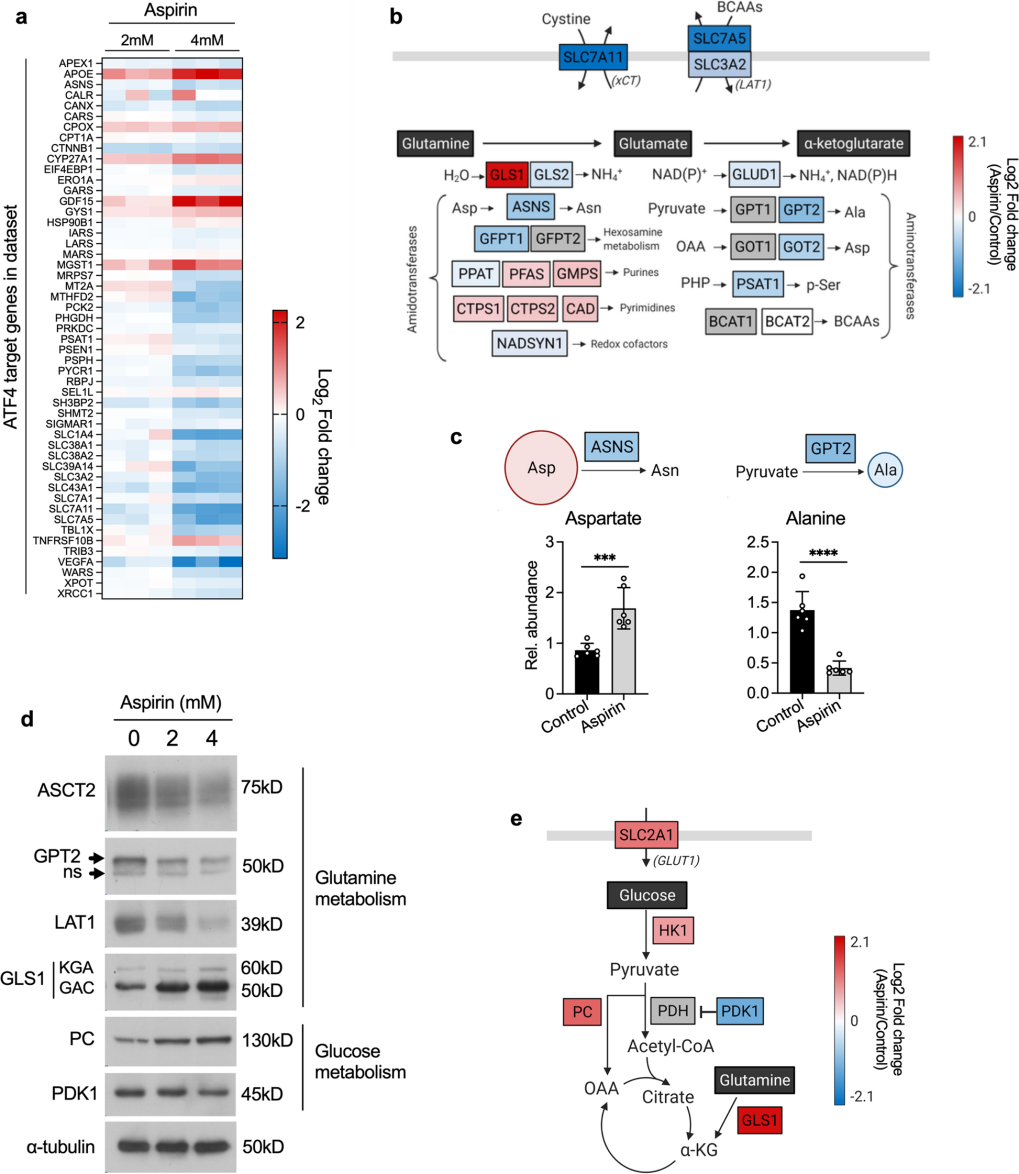
Aspirin regulates proteins involved in central carbon metabolism. **a** ATF4 target genes highlighted in proteomic data by IPA analysis. Fold change of proteins in both long-term (52 weeks) 2-mM and 4-mM aspirin conditions, relative to control (*n* = 3 biological replicates). IPA analysis shows a predicted overall inhibition of ATF4 signalling with long-term 4mM aspirin (*p* = 0.0116, *z* score =  − 2.894). **b** Overview of key enzymes involved in glutaminolysis and their average fold changes with long-term 4-mM aspirin treatment in the proteomic data. BCAAs, branched-chain amino acids; OAA, oxaloacetate, PHP, phosphohydroxypyruvate. Created with BioRender.com. **c** Metabolite abundance relative to cell number of alanine and aspartate in long-term 4-mM aspirin-treated cells compared to control. Error bars represent SD (*n* = 6 technical replicates). Asterisks refer to *p* values obtained using *t* tests ***p < 0.001, ****p < 0.0001. Created with BioRender.com. **d** Immunoblotting for a selection of metabolic enzymes highlighted in proteomic data, with long-term aspirin treatment (ns, non-specific). Representative of at least 3 independent experiments. α-tubulin is used as a loading control. **e** Average fold changes in the proteomic data with long-term 4-mM aspirin compared to control, including central carbon metabolism genes that showed significant regulation (*p* < 0.05, fold change > 1.4) in both long-term 2mM and 4mM aspirin. Created with BioRender.com

Having determined that aspirin impacts cellular metabolism in CRC cells, we sought to identify changes in protein expression that are consistent with the metabolic rewiring we observe. For this, we performed further analysis on the proteomic data in Fig. 1 and found that ingenuity pathway analysis (IPA) predicted inhibition of activating transcription factor 4 (ATF4, an important regulator of cellular metabolism) signalling with aspirin (*p* = 0.0116, z-score = -2.894). Regulation of all ATF4 target genes captured by IPA are shown in Fig. 3a.

ATF4 is involved in the cellular response to amino acid deprivation and has also been shown to regulate glutamine metabolism [34, 35]. Figure 3b shows an overview of proteins involved in glutamine metabolism and transport, several of which are ATF4 target genes. For example, expression of glutamic-pyruvic transaminase 2 (GPT2), which catalyses the reversible transamination between alanine and α-ketoglutarate (α KG) to generate pyruvate and glutamate was downregulated upon aspirin treatment (4mM aspirin vs control; log_2_ fold change =  − 0.95, *p* value = 0.03) and has been previously shown to be regulated by ATF4 [35]. There was also downregulation of ATF4 targets; asparagine synthetase (ASNS; log_2_ fold change =  − 0.75, *p* value = 0.004) and phosphoserine aminotransferase 1 (PSAT1; log_2_ fold change =  − 0.58, *p* value = 0.0008), as well as glutamate dehydrogenase 1 (GLUD1; log_2_ fold change =  − 0.27, *p* value = 0.013) and the aminotransferase glutamic-oxaloacetic transaminase 2 (GOT2; log_2_ fold change =  − 0.62, *p* value = 0.004). Furthermore, two amino acid transporters that impact glutamine metabolism; cystine/glutamate antiporter (xCT, encoded by the *SLC7A11* gene; log_2_ fold change =  − 2.09, *p* value = 0.0009) and the large neutral amino acid transporter LAT1 (encoded by the *SLC7A5* gene; log_2_ fold change =  − 1.89, *p* value = 0.012), also highlighted by the IPA in Fig. 3a, were both downregulated with aspirin. These data are consistent with the reduced levels of glutaminolysis we observed in Fig. 2. Also consistent is an increase in intracellular abundance of aspartate and a decrease in intracellular abundance of alanine upon long-term aspirin exposure, potentially illustrating the functional consequence of reduced expression of ASNS and GPT2, respectively (Fig. 3c).

Consistent with the proteomic data, mRNA levels of *GPT2*, *SLC7A11* and *SLC7A5*, were found to be downregulated upon long-term aspirin exposure, suggesting strong transcriptional regulation (Supplementary Fig. 2a). Contrary to expectation, neither protein nor mRNA levels of ATF4 itself were significantly regulated with long-term aspirin exposure (Supplementary Fig. 2b), suggesting that Aspirin might regulate ATF4 through post-translational modification (post-translational regulation of ATF4 activity has been observed previously [36]). Validation of proteomic data was performed for GPT2 and LAT1 by immunoblotting (Fig. 3d and Supplementary Fig. 2c). The key glutamine transporter ASCT2 (alanine-serine-cysteine transporter 2) was also investigated by immunoblotting and was downregulated with long-term 4mM aspirin (Fig. 3d), although this was not statistically significantly (Supplementary Fig. 2c).

Further analysis of the proteomic data also revealed expression changes consistent with the concomitant increase in glucose metabolism we observed in Fig. 2f. Two proteins involved in regulating the entry of pyruvate into the TCA cycle showed significant regulation—PC was upregulated (log_2_ fold change =  + 1.28, *p* value = 0.00037) and PDK1 was downregulated (log_2_ fold change =  − 1.19, *p* value = 0.002) (illustrated in Fig. 3e). These results were validated by immunoblotting (Fig. 3d and Supplementary Fig. 2d). This is consistent with the increased glucose carbon entry into the TCA cycle that we previously observed (Fig. 2f and h). In addition, glycolysis enzyme hexokinase 1 (HK1; log_2_ fold change =  + 0.79, *p* value = 0.012) and glucose transporter 1 (GLUT1, encoded by the *SLC2A1* gene; log_2_ fold change =  + 1.13, *p* value = 0.00026) were both upregulated in the proteomic data, also consistent with increased glucose utilisation upon aspirin exposure (Fig. 3e). These results were validated by immunoblotting (Supplementary Fig. 2e).

Unexpectedly, GLS1, which catalyses the first step of glutaminolysis (Fig. 3b), showed significant upregulation (Fig. 3d, e and Supplementary Fig. 2d). This is inconsistent with the reduced levels of glutaminolysis we observed in Fig. 2. Both known splice variants of GLS1 (GLS1^KGA^ and GLS1^GAC^) were identified (Fig. 3d), with GLS1^GAC^ being the dominantly expressed isoform in our cells. qPCR analysis of aspirin-treated SW620 cells did not show any significant transcriptional regulation of *GLS1*, *PC* or *PDK1* (Supplementary Fig. 2f), suggesting post-transcriptional regulation of these proteins.

To demonstrate the changes in other CRC cell lines, expression of GLS1, PC and PDK1 was also investigated by immunoblotting in LS174T and HCA7 cells after long-term aspirin exposure (Supplementary Fig. 3a), showing upregulation of GLS1^GAC^ in LS174T, though this is not statistically significant, and significant downregulation of PC and PDK1 in HCA7 cells.

Expression changes were also investigated by immunoblotting following short-term aspirin treatment (72 h) (Supplementary Figs. 3a-b). This showed significant upregulation of GLS1^GAC^ in SW620 and LS174T cells, as well as upregulation of PC in SW620. In addition, downregulation of mRNA expression of the ATF4 targets *GPT2* and *SLC7A5* was also observed in SW620 cells (Supplementary Fig. 3c). Interestingly, these findings suggest short-term treatment is sufficient for the regulatory effect of aspirin on these metabolic enzymes. However, it should be noted that long-term aspirin exposure has a stronger effect.

### Metabolic reprogramming in response to aspirin exposes metabolic vulnerabilities in CRC cells

**Fig. 4:**
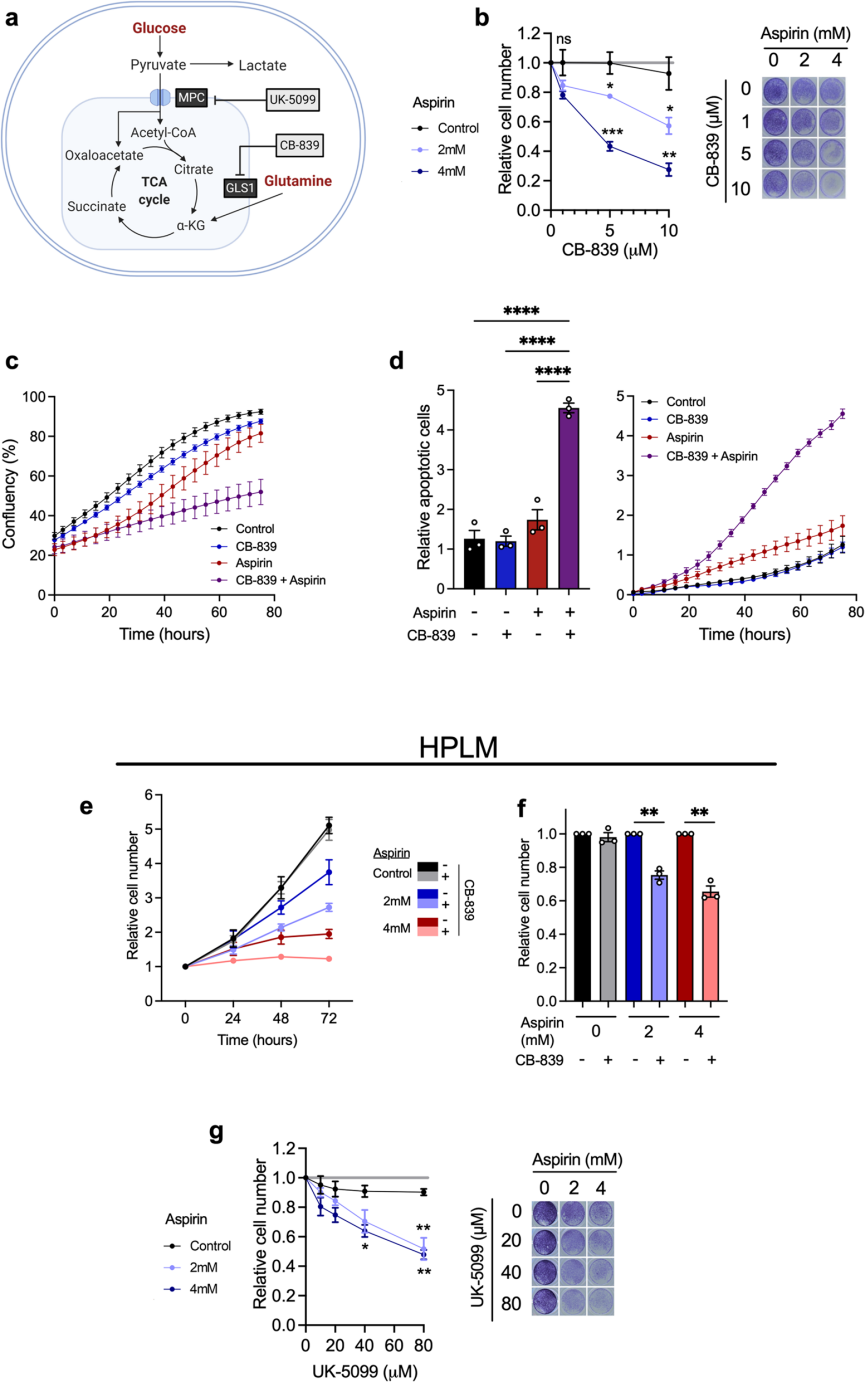
Aspirin treatment sensitises CRC cells to metabolic inhibitors. **a** Schematic showing the mechanism of action of metabolic inhibitors; CB-839 and UK-5099. Created with BioRender.com. **b** Cell proliferation assay of long-term (52 weeks) aspirin-treated SW620 cells with increasing concentration of CB-839. The graph shows the relative cell number in each aspirin condition, measured by crystal violet staining at 72 h compared to vehicle control. Error bars show SEM (*n* = 3 independent experiments). Asterisks refer to *p* values obtained using one-way ANOVAs with Dunnett’s multiple comparisons tests at each CB-839 concentration (*p < 0.05, **p < 0.01, ***p < 0.001). Images show representative wells in each condition at 72 h. **c**, **d** Confluency and relative apoptotic cells in long-term 4-mM aspirin-treated SW620 cells in combination with 5µM CB-839, in comparison to controls and to each drug alone. Error bars show SEM (*n* = 3 distinct passages of cells analysed on the same experimental plate). Relative apoptotic cells were measured by green fluorescent nuclei (indicating cells with activated caspase-3/7) relative to cell confluency. Line graphs show values over time, and the bar graph shows the values at the experiment endpoint (75 h after treatment). Error bars show SEM (*n* = 3 distinct passages of cells analysed on the same experimental plate). Asterisks refer to *p* values obtained using a one-way ANOVA with Tukey’s multiple comparisons test (****p < 0.0001). **e**, **f** Proliferation assay of SW620 cells treated with aspirin and/or CB-839 compared to vehicle control for 72 h, performed in Human Plasma-Like Medium (HPLM). **e** Cell number over time relative to the 0 h time point. **f** Relative cell number at 72 h, 5µM CB-839 treatment condition is shown relative to vehicle control in the same aspirin treatment condition. Error bars show SEM (*n* = 3 independent experiments). Asterisks refer to *p* values obtained using one sample *t* tests, comparing to a hypothetical mean of 1 (**p < 0.01). **g** Cell proliferation assay of long-term aspirin-treated SW620 cells with increasing concentration of UK-5099. The graph shows a relative cell number in each aspirin condition measured by crystal violet staining at 72 h. Error bars show SEM (*n* = 3 independent experiments). Asterisks refer to *p* values obtained using one-way ANOVAs with Dunnett’s multiple comparisons tests at each UK-5099 concentration (*p < 0.05, **p < 0.01, ***p < 0.001). Images show representative wells in each condition at 72 h

While the metabolic impact of aspirin may be insufficient to explain the known detrimental effect on cellular proliferation [30], it could render cells more susceptible to further metabolic perturbation. Despite an overall reduction in glutaminolysis (Fig. 2g), aspirin causes a strong upregulation in GLS1 levels (Fig. 3d), which may be a compensatory mechanism to maximise utilisation of glutamine when levels of other glutaminolysis enzymes are reduced (such as GPT2). Increased expression of GPT2 has been previously shown to compensate for inhibition of GLS1, suggesting that the reverse relationship may also occur [37]. We therefore hypothesised that aspirin-treated cells may be more sensitive to further blockade of glutaminolysis by targeting GLS1. To investigate this, long-term aspirin-exposed cells were incubated with increasing concentrations of CB-839 (a selective GLS1 inhibitor currently in clinical trials [38], also known as Telaglenastat, illustrated in Fig. 4a). SW620 cells showed no sensitivity to CB-839 alone (up to 10µM), consistent with previous findings [39]; however, cells exposed to long-term aspirin showed significantly increased sensitivity in a dose-dependent manner (Fig. 4b). Similar results were obtained using another GLS1 inhibitor, inhibitor-968 (Supplementary Fig. 4a), demonstrating the specificity of the aspirin effect on glutaminolysis. Similar results were obtained in long-term aspirin-treated HCA7 cells and to a lesser extent LS174T. Although both of these cell lines showed minimal sensitivity to CB-839 without aspirin, aspirin significantly increased their response to the drug (Supplementary Fig. 4b-c). This effect was also investigated with short-term aspirin treatment in SW620 cells (Supplementary Fig. 4d). This also showed sensitisation, but to a lesser extent than in long-term aspirin-treated cells.

To further investigate the effect of CB-839 on long-term aspirin-exposed cells, proliferation assays were performed alongside the detection of caspase-3/7 activation to quantify levels of apoptosis using an Incucyte® Live-Cell Analysis System (Fig. 4c, d). These results confirm the inhibitory effect on the proliferation of combined CB-839 and long-term aspirin, as shown by a decrease in confluency (Fig. 4c). These results also show significant induction of apoptosis with the combination of CB-839 and long-term aspirin treatment compared to vehicle control and to either drug alone (Fig. 4d). A positive control for this assay was performed using ABT-737 to induce apoptosis (Supplementary Fig. 4e-f).

Proliferation experiments were also performed using the human plasma-like medium (HPLM), developed by Cantor et al. to be representative of the metabolite composition of human plasma [40]. Similar results were obtained in HPLM to those performed in DMEM (Fig. 4e, f). Both 2mM and 4mM aspirin inhibited proliferation compared to controls. The addition of CB-839 in the absence of aspirin had no effect on proliferation, whereas it significantly reduced cell number with both 2mM and 4mM aspirin. This demonstrates that the effect of aspirin on sensitising cells to CB-839 is present in physiologically relevant metabolic conditions.

Upon long-term aspirin exposure, we have shown that glutaminolysis is reduced (Fig. 2j), leading to a potentially compensatory increase in (Fig. 3b and d), and dependence on, GLS1 (Fig. 4b–f). We hypothesised that the increase in glucose utilisation we observed in Fig. 2f is another compensatory response to impaired glutaminolysis in order to maintain TCA cycle activity. We reasoned this could leave cells vulnerable to inhibition of glucose utilisation and specifically to pyruvate import into the mitochondria. We investigated this by treating cells exposed to long-term aspirin with increasing concentrations of an inhibitor of the mitochondrial pyruvate carrier 1 (MPC1), UK-5099. UK-5099 inhibits the entry of glucose-derived pyruvate into the TCA cycle (illustrated in Fig. 4a). SW620 cells showed little or no sensitivity to UK-5099 alone, but sensitivity was significantly increased in cells exposed to both 2mM and 4mM aspirin (Fig. 4g). HCA7 cells showed a similar effect to SW620 cells; however, LS174T cells did not show significantly increased sensitivity to UK-5099 with long-term aspirin (Supplementary Fig. 4b-c), suggesting some cell-line specificity in this response. This effect was also investigated upon short-term aspirin treatment in SW620 cells (Supplementary Fig. 4d), which also increased sensitivity to UK-5099.

**Fig. 5:**
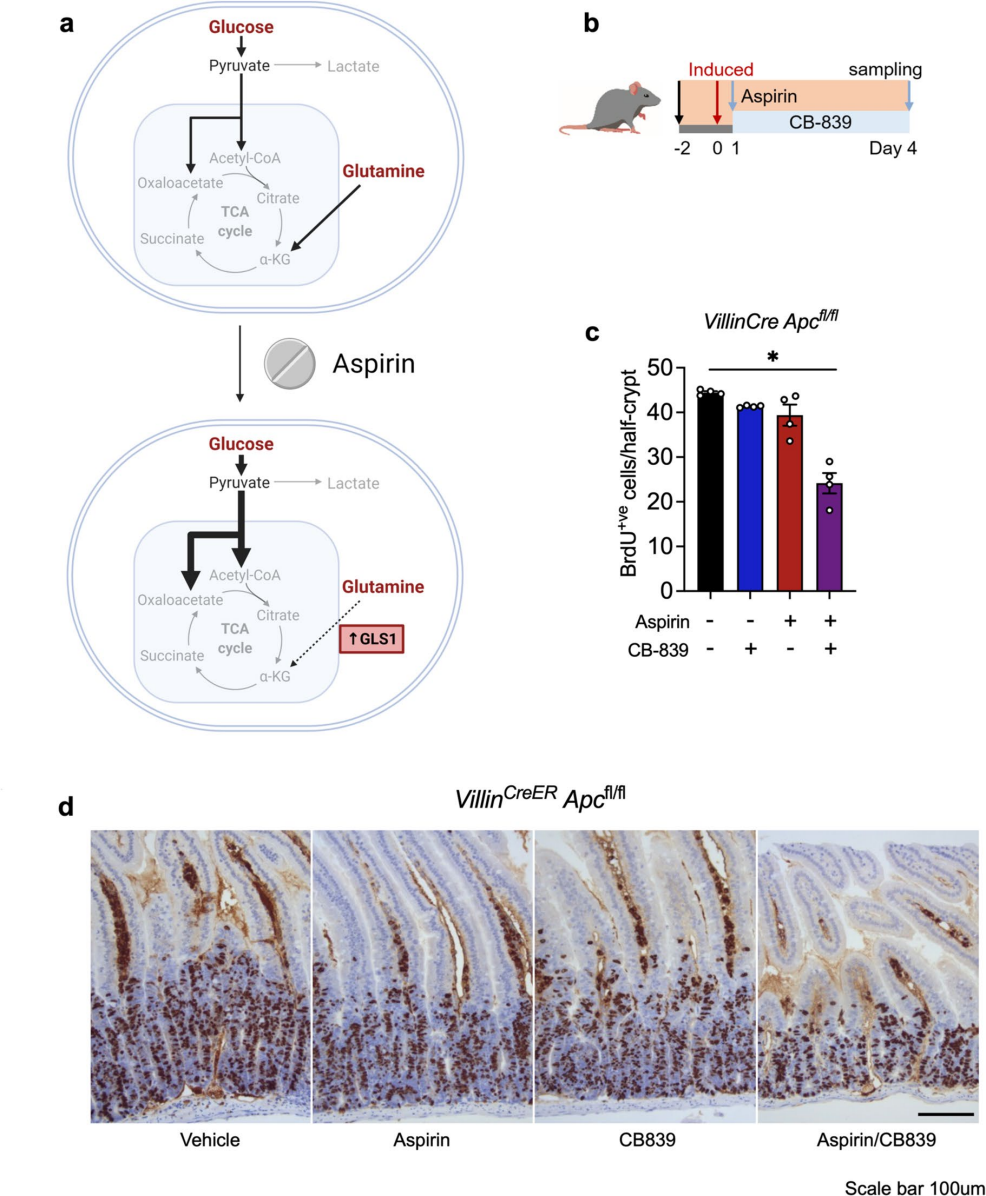
Aspirin and CB-389 in combination reduce colon crypt proliferation in vivo*. ***a** Schematic summarising the effects of aspirin treatment on metabolic reprogramming of CRC cells. Created with BioRender.com. **b** Schematic showing the timeline of treatment, induction with tamoxifen (2 mg) and sampling for in vivo aspirin (2.6mg/ml in drinking water) and CB-839 (200mg/kg) combination experiments using the *Villin*^*CreER*^* Apc*^fl/fl^ mouse model.** c**, **d** Quantification of BrdU and representative images of BrdU staining in the small intestine of *Villin*^*CreER*^* Apc*.^fl/fl^ mice treated with vehicle, CB-839 (200mg/kg) and/or aspirin (2.6mg/ml in drinking water). Scale bar 100μm. Error bars show SEM (*n* = 4 mice per experimental arm). Each dot represents the average number of Brd-positive cells per half crypt for each mouse. Asterisks refer to *p* values obtained from one-tailed Mann–Whitney tests (*p < 0.05; **p < 0.01)

These findings support the hypothesis that when treated with aspirin, cells reprogramme their metabolism in order to maintain proliferation (summarised in Fig. 5a), leaving them vulnerable to further metabolic manipulation. While they have sufficient metabolic plasticity to prevent an impact on ATP production and complete inhibition of proliferation in the presence of aspirin alone, the cells become more reliant on particular metabolic pathways and are left vulnerable to their targeting, leading to further impaired proliferation and cell death.

### Aspirin and CB-389 in combination reduce colon crypt proliferation in vivo

We next sought to investigate the efficacy of combining aspirin and CB-839 in vivo. To achieve this, we used the well-characterized VillinCreER Apcfl/fl mouse model. The mice were induced with tamoxifen (2mg on two consecutive days) to conditionally delete Apc throughout the intestinal epithelium. The mice were treated with either vehicle or aspirin (2.6mg/ml) or CB-839 (200mg/kg) alone or in combination (aspirin + CB-839), and the effect on intestinal epithelial cell proliferation was investigated (treatments summarised in Fig. 5b). Apc loss leads to a characteristic crypt hyperproliferation in this model as assessed by BrdU incorporation and quantification of the number of stained BrdU + cells in each crypt (Fig. 5c, d). Strikingly, the combination of aspirin (2.6mg/ml) and CB-839 (200mg/kg) led to a significant suppression of crypt hyperproliferation in the small intestine as indicated by BrdU-stained cells, while neither aspirin nor CB-839 alone impacted proliferation (Fig. 5c, d). Villi length was unchanged across conditions (Supplementary Fig. 5). These results support our in vitro findings showing that aspirin induces sensitivity to CB-839 and support the potential for clinical utility of this approach.

## Discussion

Further understanding of the anti-cancer cellular mechanisms of aspirin will be beneficial to inform patient stratification and maximise its benefits. Although best known as a cyclooxygenase (COX) inhibitor, aspirin is a highly pleiotropic drug with many cellular targets. Despite the importance of COX/PGE2 (prostaglandin E2) signalling in cancer [41], this mechanism is not sufficient to fully explain the anti-cancer effects of aspirin [42]. Studies have shown aspirin treatment impacts many other pathways including Wnt [43], NF-κB [44, 45], AMPK (adenosine monophosphate-activated protein kinase) and mTORC1 (mammalian target of rapamycin complex 1) [46, 47], as well as causing epigenetic alterations such as histone methylation [48, 49]; however, relatively little work has focused on the impact of aspirin treatment on cancer cell metabolism.

Here, we used proteomics with the aim to identify novel cellular mechanisms of aspirin that may contribute to its anti-cancer effect. This analysis highlighted a potential effect on cellular metabolism which was subsequently confirmed; using a combination of extracellular flux analysis and SIL, we comprehensively characterised the effect of aspirin on CRC cell metabolic pathway activity. Our results show that although there is no overall impact of aspirin on ATP production, long-term aspirin exposure leads to significant reprogramming of nutrient utilisation, leading to a reduction in glutaminolysis and increased glucose entry into the TCA cycle in three CRC cell lines (summarised in Fig. 5a).

We also show regulation of proteins involved in central carbon metabolism that is consistent with the effects on pathway activity, including increased expression of PC and decreased expression of PDK1 and regulation of several glutaminolysis enzymes. Surprisingly, there was a strong increase in the expression of GLS1, which is contradictory to the observed decrease in net glutaminolysis. We suggest that this is a compensatory response to glutaminolysis being otherwise impaired. Increased glucose utilisation is also a likely compensatory mechanism to reduced glutaminolysis, as both nutrients provide important carbon sources for the TCA cycle. This information was used to infer potential metabolic vulnerabilities induced by aspirin; we show that aspirin-treated cells are more sensitive to the GLS1 inhibitor CB-839 and MPC1 inhibitor UK-5099. Importantly, the combination of aspirin and CB-839 was found to be effective in reducing cell proliferation both in vitro and in vivo; the treatment inhibited proliferation and induced apoptosis in CRC cell lines and inhibited proliferation of colonic epithelial cells in *VillinCre*; *Apc*^*fl/fl*^ mice.

A small number of recent studies have linked aspirin’s anti-cancer effects to metabolism; aspirin has been found to inhibit glucose metabolism through the regulation of PDK1 in breast cancer cells [50] and through the regulation of GLUT1 in hepatoma cells [51]. In support of our study, Boku et al. show that combining GLS1 inhibition with aspirin is effective at reducing the colony-forming efficiency of CRC cells in vitro [52]. The same study suggested that aspirin treatment mimics the effects of glutamine deficiency in CRC cells and showed increased expression of glutaminolysis genes with aspirin treatment [52]. The authors concluded that glutaminolysis is therefore likely upregulated upon aspirin treatment; however, this was not directly measured. Interestingly, our functional studies of glutaminolysis using SIL show a reduction in glutaminolysis with aspirin. We conclude that the upregulation of GLS1 is a likely compensatory mechanism for reduced glutaminolysis. Despite these apparent differences, both studies highlight the possibility that aspirin has potential to be a simple and cost-effective drug to increase the efficacy of CB-839 in CRC patients. This is important as despite the attractiveness of targeting glutamine metabolism for cancer therapy [53], CB-839 has achieved varying success in previous studies, particularly when studied in vivo [54, 55]. Indeed, in our study, CB-839 alone was almost completely ineffective at inhibiting cell proliferation and had no effect on apoptosis in vitro or crypt proliferation in vivo. Therefore, our findings have exciting implications for clinical translation, as both drugs are already used clinically and have known safety profiles in humans.

An increasing number of studies highlight the value of combinatory approaches when applying metabolic interventions. One comprehensive study highlights several combinations of metabolic inhibition that overcome the ability of cancer cells to adapt their metabolism in response to singular perturbations—known as metabolic flexibility or plasticity [32]. A recent study showed that combining CB-839 with an inhibitor of ASCT2 was effective in liver cancer cells [56]. Several studies have also highlighted success when combining metabolic interventions with chemotherapies [57–59] and immunotherapies [60]. There is also increasing interest in the impact of diet on tumour metabolism and how this may interact with metabolic therapies [61]. Our findings support the value of combining multiple complementary interventions when targeting tumour metabolism. By highlighting metabolic vulnerabilities caused by the exposure of CRC to aspirin alone, we were able to exploit these to maximise the inhibition of CRC cell proliferation with metabolic inhibitors. The use of aspirin to increase the efficacy of metabolic inhibitors is particularly valuable due to the longstanding ubiquitous use of aspirin in clinical practice. The efficacy of aspirin and CB-839 in vivo shown here suggest that this combination warrants further clinical studies and could potentially provide a novel therapeutic option for CRC patients alongside traditional chemotherapies. An important next step should involve further in vivo experiments assessing the efficacy of the combination of aspirin and CB-839 on overall tumour growth, as the short-term proliferation defect we observe may not necessarily translate to reduced tumour growth in mouse models of colorectal cancer.

## Conclusions

Aspirin leads to significant reprogramming of glucose and glutamine metabolism in CRC cells. While this has no effect on overall ATP production, it renders cells vulnerable to further metabolic perturbation. Aspirin-exposed cells show increased sensitivity to inhibition of GLS1 by CB-839 and glucose utilisation by UK-5099. The combination of aspirin and CB-839 was also effective at reducing cell proliferation in vivo, therefore having exciting implications for clinical translation: CB-839 is currently under investigation in the clinic to treat various tumour types, and this study suggests that aspirin may significantly increase the efficacy of this drug in CRC. Further investigation into this combination is warranted to determine whether it could provide an effective and safe treatment option for CRC.

### Supplementary Information

**Additional file 1: Figure S1.** Aspirin treatment reprogrammes nutrient utilisation in CRC cells. Proportion of ^13^C labelling in downstream metabolites after 8 h incubation with either U-[^13^C]-Glc or U-[^13^C]-Q in LS174T and HCA7 cells with long-term 4mM aspirin treatment compared to control cells. Error bars represent SD (*n* = 3 technical replicates). Asterisks refer to adjusted p-values obtained from multiple t tests (* = *p* < 0.05, ** = *p* < 0.01, *** = *p* < 0.001, **** = *p* < 0.0001). **Figure S2.** Long-term aspirin treatment regulates metabolic enzyme expression in SW620 cells. a-e) qPCR analysis and quantification of immunoblot of metabolic gene expression in long-term (52 week) aspirin exposed SW620 cells. Error bars represent SEM (n≥3 independent experiments). Asterisks refer to p-values obtained from one-sample t tests, comparing to a hypothetical mean of 1 (* = *p* < 0.05, ** = *p* < 0.01, *** = *p* < 0.001). a) qPCR analysis of ATF4 target gene expression. b) qPCR and quantified immunoblot analysis of ATF4 expression. Immunoblot image shows a representative blot of 3 independent experiments, α-tubulin is used as a loading control. c) Quantification of immunoblots for proteins involved in glutamine metabolism. d) Quantification of immunoblot for proteins involved in central carbon metabolism. GLS1^GAC^ = GAC splice isoform of glutaminase 1. e) Representation immunoblot image of 3 independent experiments and quantified immunoblot analysis of HK1 and GLUT1 expression, α-tubulin is used as a loading control. f) Relative mRNA levels, determined by qPCR of genes involved in central carbon metabolism. **Figure S3.** Long-term and short-term aspirin treatment regulates metabolic enzyme expression in three CRC cell lines. a-b) Graphs show quantification of immunoblotting, error bars represent SEM (*n* = 3 independent experiments). Asterisks refer to p-values obtained from one-sample t tests, comparing to a hypothetical mean of 1 (* = *p* < 0.05, *** = *p* < 0.001) Immunoblot images are representative of three independent experiments, α-tubulin is used as a loading control. GLS1^GAC^ = GAC splice isoform of glutaminase 1. a) Short-term (72 h) and long-term aspirin (52 week) treated LS174T and HCA7 cells. b) Short-term (72 h) aspirin treated SW620 cells. c) qPCR analysis of ATF4 target genes in short-term (72 h) aspirin treated SW620 cells. Error bars represent SEM (*n* = 3 independent experiments), asterisks refer to p-values obtained from one-sample t tests, comparing to a hypothetical mean of 1 (* = *p* < 0.05, ** = *p* < 0.01). **Figure S4.** Aspirin sensitises CRC cells to metabolic inhibitors. a-d) Cell proliferation assays of long-term (52 week) aspirin treated SW620 (a) LS174T (b) and HCA7 cells (c) and short-term (72 h) aspirin treated SW620 cells (d) with increasing concentrations of either inhibitor 968, CB-839 or UK-5099. Graphs show relative cell number in each aspirin condition as measured by crystal violet staining at 72 h of drug treatment compared to vehicle control. Error bars show SEM (*n* = 5 independent experiments for HCA7 with CB-839, *n* = 3 independent experiments for all other experiments). Asterisks refer to p-values obtained using one-way ANOVAs with Dunnett’s multiple comparisons tests at each drug concentration (* = *p* < 0.05, ** = *p* < 0.01, *** = *p* < 0.001). Images show representative wells in each condition after 72 h of treatment. e) Quantification of green fluorescent nuclei indicating apoptotic SW620 cells with activated caspase-3/7, with 2µM ABT-737 treatment compared to control, as a positive control for apoptosis. Error bars represent SD (n = 3 technical replicates). f) Representative images of 2µM ABT-737 treated and control SW620 cells at the end of the assay (~ 96 h). Scale bar represents 300µm. **Figure S5.** Aspirin and CB-389 in combination reduce colon crypt proliferation in vivo*.* Quantification of the villi length in small intestine of *Villin*^*CreER*^* Apc*^fl/fl^ mice treated with CB-839 (200mg/kg) and/or aspirin (2.6mg/ml in drinking water). Error bars show SEM (n = 4 mice per experimental arm). Each dot represents the average length of villi for each mouse. Statistical analysis (ns, *p* > 0.05), one-way ANOVA with Tukey or Bonferroni post hoc test.

**Additional file 2: Table S1.** Metabolomics Data. **Table S2.** Proteomics Data. **Table S3.** Uncropped western scans

## Data Availability

Raw metabolomics and proteomics data generated and analysed during this study are included in this published article (Supplementary Data File S1 and S2, respectively). All other raw datasets generated and analysed during the current study are available from the corresponding author on reasonable request.

## Abbreviations

αKG: α-Ketoglutarate
AMPK: Adenosine monophosphate-activated protein kinase
ASNS: Asparagine synthetase
ASCT2: Alanine-serine-cysteine transporter 2
ATF4: Activating transcription factor 4
ATP: Adenosine triphosphate
COX: Cyclooxygenase
CRC: Colorectal cancer
DMEM: Dulbecco’s modified Eagle’s medium
DMSO: Dimethyl sulfoxide
ECAR: Extracellular acidification rate
(d)FBS: (Dialysed) Foetal bovine serum
FDR: False discovery rate
GCMS: Gas chromatography mass spectrometry
GLS1: Glutaminase 1
GLUD1: Glutamate dehydrogenase 1
GLUT1: Glucose transporter 1
GO: Gene ontology
GOT2: Glutamic-oxaloacetic transaminase 2
GPT2: Glutamic-pyruvic transaminase 2
H&E: Haematoxylin and eosin
HK1: Hexokinase 1
HPLM: Human plasma-like serum
IHC: Immunohistochemistry
IPA: Ingenuity pathway analysis
KEGG: Kyoto Encyclopedia of Genes and Genomes
LAT1: Large neutral amino acid transporter
LCMS: Liquid chromatography mass spectrometry
MID: Mass isotopomer distribution
MPC1: Mitochondrial pyruvate carrier 1
MTBSTFA: *N*-(Tert-butyldimethylsilyl)-*N*-methyltrifluoroacetamide
mTORC1: Mammalian target of rapamycin complex 1
NICE: National Institute for Health and Care Excellence
NSAID: Non-steroidal anti-inflammatory drug
OCR: Oxygen consumption rate
Oxphos: Oxidative phosphorylation
PC: Pyruvate carboxylase
PDK1: Pyruvate dehydrogenase kinase 1
PGE2: Prostaglandin E2
PSAT1: Phosphoserine aminotransferase 1
qPCR: Quantitative polymerase chain reaction
SIL: Stable isotope labelling
TCA: Tricarboxylic acid cycle
TMT: Tandem mass tag
xCT: Cystine/glutamate antiporter
