# Supplementary material for: Aspirin reprogrammes colorectal cancer cell metabolism and sensitises to glutaminase inhibition
Source: Cancer Metab. 2023 Oct 19;11:18. doi: 10.1186/s40170-023-00318-y (PMC10588174; doi:10.1186/s40170-023-00318-y)
Supplement: Supplementary file 1 — Additional file 1: Figure S1. Aspirin treatment reprogrammes nutrient utilisation in CRC cells. Proportion of 13C labelling in downstream metabolites after 8 h incubation with either U-[13C]-Glc or U-[13C]-Q in LS174T and HCA7 cells with long-term 4mM aspirin treatment compared to control cells. Error bars represent SD (n = 3 technical replicates). Asterisks refer to adjusted p-values obtained from multiple t tests (* = p < 0.05, ** = p < 0.01, *** = p < 0.001, **** = p < 0.0001). Figure S2. Long-term aspirin treatment regulates metabolic enzyme expression in SW620 cells. a-e) qPCR analysis and quantification of immunoblot of metabolic gene expression in long-term (52 week) aspirin exposed SW620 cells. Error bars represent SEM (n≥3 independent experiments). Asterisks refer to p-values obtained from one-sample t tests, comparing to a hypothetical mean of 1 (* = p < 0.05, ** = p < 0.01, *** = p < 0.001). a) qPCR analysis of ATF4 target gene expression. b) qPCR and quantified immunoblot analysis of ATF4 expression. Immunoblot image shows a representative blot of 3 independent experiments, α-tubulin is used as a loading control. c) Quantification of immunoblots for proteins involved in glutamine metabolism. d) Quantification of immunoblot for proteins involved in central carbon metabolism. GLS1GAC = GAC splice isoform of glutaminase 1. e) Representation immunoblot image of 3 independent experiments and quantified immunoblot analysis of HK1 and GLUT1 expression, α-tubulin is used as a loading control. f) Relative mRNA levels, determined by qPCR of genes involved in central carbon metabolism. Figure S3. Long-term and short-term aspirin treatment regulates metabolic enzyme expression in three CRC cell lines. a-b) Graphs show quantification of immunoblotting, error bars represent SEM (n = 3 independent experiments). Asterisks refer to p-values obtained from one-sample t tests, comparing to a hypothetical mean of 1 (* = p < 0.05, *** = p < 0.001) Immunoblot images are repres [file 40170_2023_318_MOESM1_ESM.docx]

**Supplementary material**

**Aspirin reprogrammes colorectal cancer cell metabolism and sensitises to glutaminase inhibition**

Amy K. Holt^1^, Arafath K. Najumudeen^2,3^, Tracey J. Collard^1^, Hao Li^3^, Laura M. Millett^2^, Ashley J. Hoskin^1^, Danny N. Legge^4^, Eleanor M.H. Mortensson^1^, Dustin J. Flanagan^2^, Nicholas Jones^5^, Madhu Kollareddy^1^, Penny Timms^1^, Matthew D Hitchings^5^, James Cronin^5^, Owen J. Sansom^2,6^, Ann C. Williams^1*^ & Emma E. Vincent^3,7*^

^1^School of Cellular and Molecular Medicine, Biomedical Sciences Building, University of Bristol, BS8 1TW, UK

^2^Cancer Research UK Beatson Institute, Glasgow, G61 1BD, UK

^3^Institute of Biotechnology, HiLIFE, University of Helsinki, Helsinki, Finland

^4^School of Translational Health Sciences, Dorothy Hodgkin Building, University of Bristol, BS1 3NY, UK

^5^Institute of Life Science, Swansea University Medical School, Swansea University, SA2 8PP, UK

^6^Institute of Cancer Sciences, University of Glasgow, Glasgow, G61 1QH, UK

^7^MRC Integrative Epidemiology Unit, Oakfield House, University of Bristol, Bristol, BS8 2BN, UK

*Joint senior authors

**Corresponding author:**

Emma E. Vincent, emma.vincent@bristol.ac.uk (E.E.V.)

**
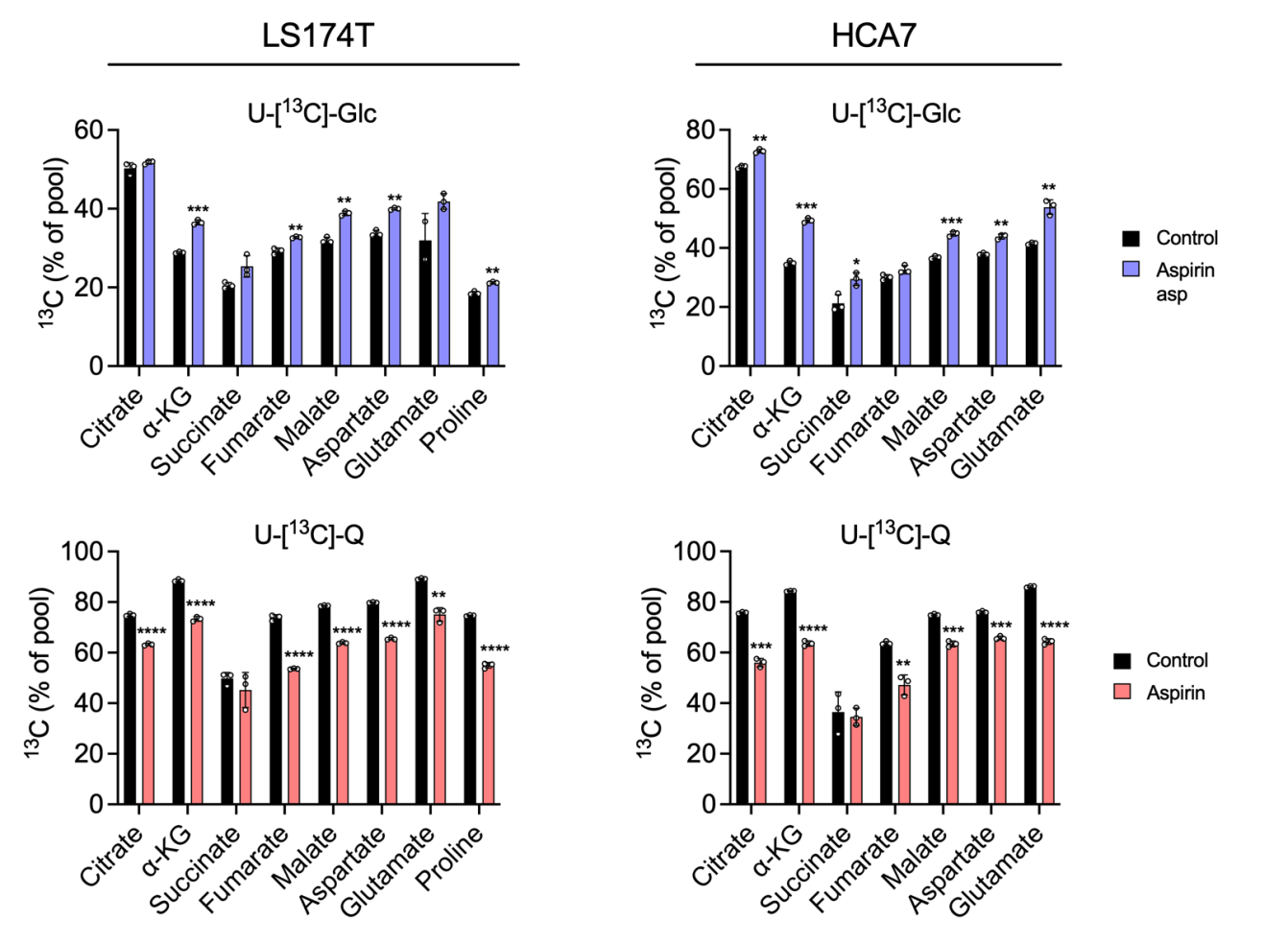
**

**Supplementary Figure 1. Aspirin treatment reprogrammes nutrient utilisation in CRC cells.** Proportion of ^13^C labelling in downstream metabolites after 8 hours incubation with either U-[^13^C]-Glc or U-[^13^C]-Q in LS174T and HCA7 cells with long-term 4mM aspirin treatment compared to control cells. Error bars represent SD (n=3 technical replicates). Asterisks refer to adjusted p-values obtained from multiple t tests (*=p<0.05, **=p<0.01, ***=p<0.001, ****=p<0.0001).

**
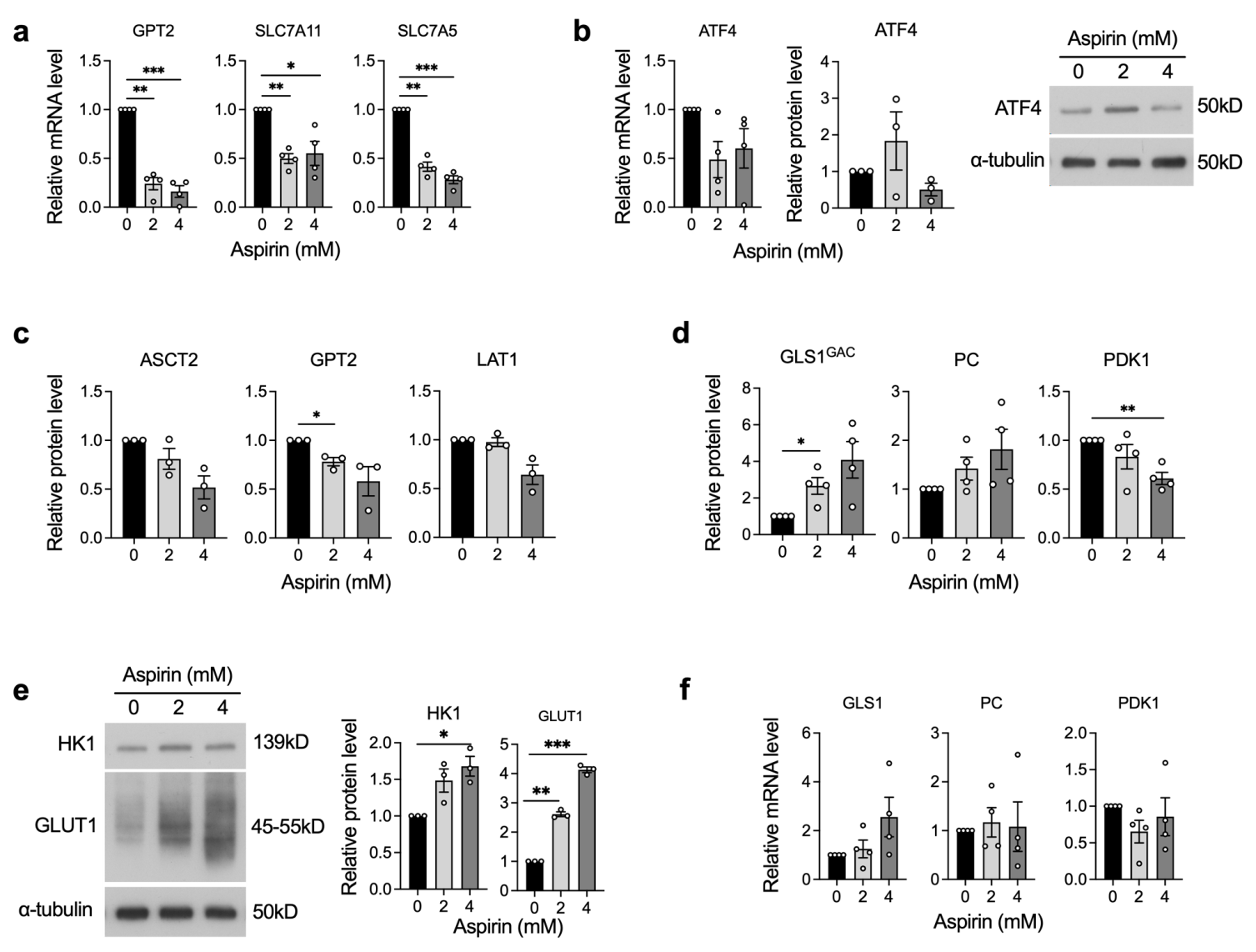
**

**Supplementary Figure 2. Long-term aspirin treatment regulates metabolic enzyme expression in SW620 cells. a-e)** qPCR analysis and quantification of immunoblot of metabolic gene expression in long-term (52 week) aspirin exposed SW620 cells. Error bars represent SEM (n≥3 independent experiments). Asterisks refer to p-values obtained from one-sample t tests, comparing to a hypothetical mean of 1 (*=p<0.05, **=p<0.01, ***=p<0.001). **a**) qPCR analysis of ATF4 target gene expression. **b)** qPCR and quantified immunoblot analysis of ATF4 expression. Immunoblot image shows a representative blot of 3 independent experiments, α-tubulin is used as a loading control. **c)** Quantification of immunoblots for proteins involved in glutamine metabolism. **d)** Quantification of immunoblot for proteins involved in central carbon metabolism. GLS1^GAC^ = GAC splice isoform of glutaminase 1. **e)** Representation immunoblot image of 3 independent experiments and quantified immunoblot analysis of HK1 and GLUT1 expression, α-tubulin is used as a loading control. **f)** Relative mRNA levels, determined by qPCR of genes involved in central carbon metabolism.

**
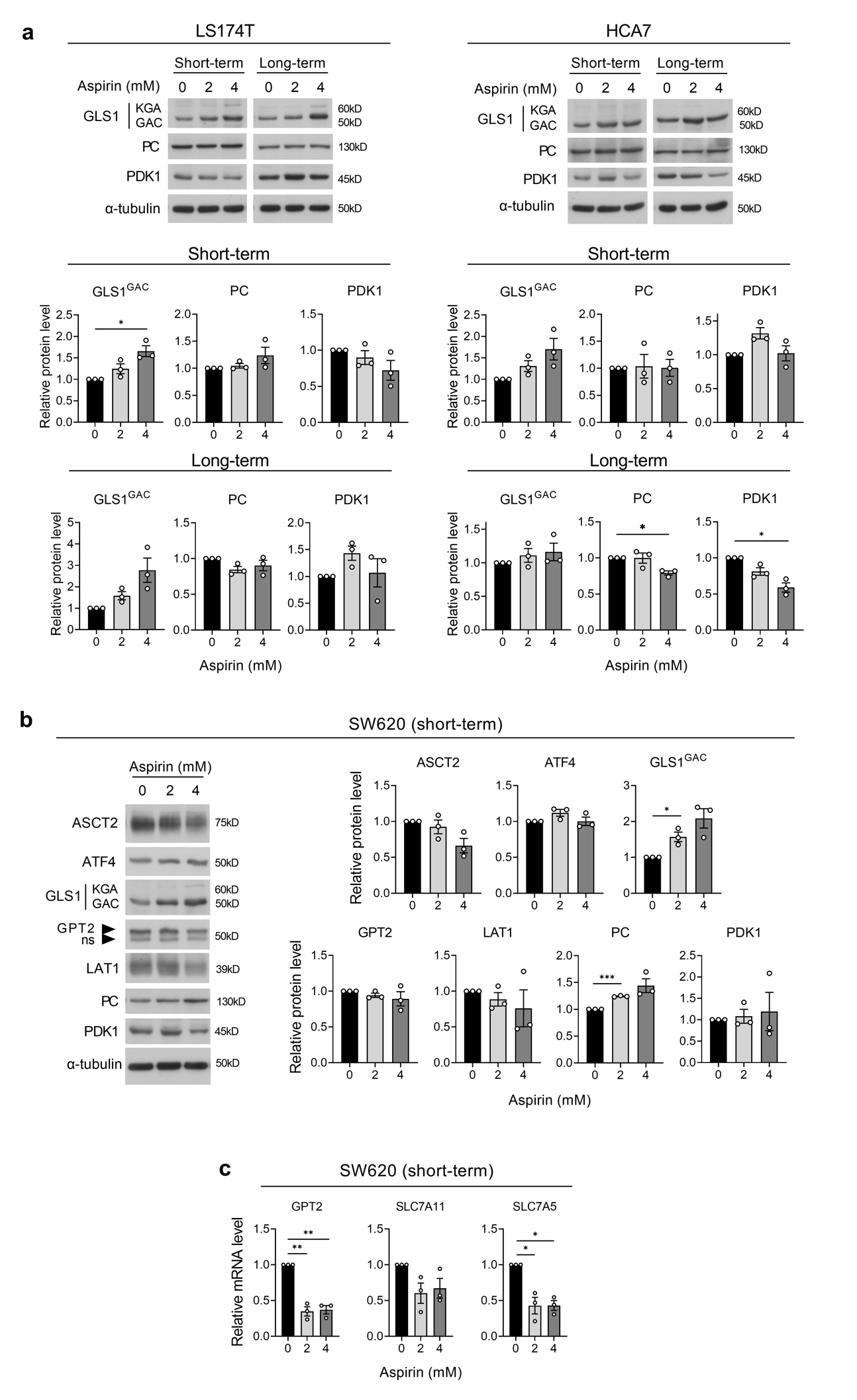
**

**Supplementary Figure 3. Long-term and short-term aspirin treatment regulates metabolic enzyme expression in three CRC cell lines. a-b)** Graphs show quantification of immunoblotting, error bars represent SEM (n=3 independent experiments). Asterisks refer to p-values obtained from one-sample t tests, comparing to a hypothetical mean of 1 (*=p<0.05, ***=p<0.001) Immunoblot images are representative of three independent experiments, α-tubulin is used as a loading control. GLS1^GAC^ = GAC splice isoform of glutaminase 1. **a)** Short-term (72 hour) and long-term aspirin (52 week) treated LS174T and HCA7 cells. **b)** Short-term (72 hour) aspirin treated SW620 cells. **c)** qPCR analysis of ATF4 target genes in short-term (72 hour) aspirin treated SW620 cells. Error bars represent SEM (n=3 independent experiments), asterisks refer to p-values obtained from one-sample t tests, comparing to a hypothetical mean of 1 (*=p<0.05, **=p<0.01).

**
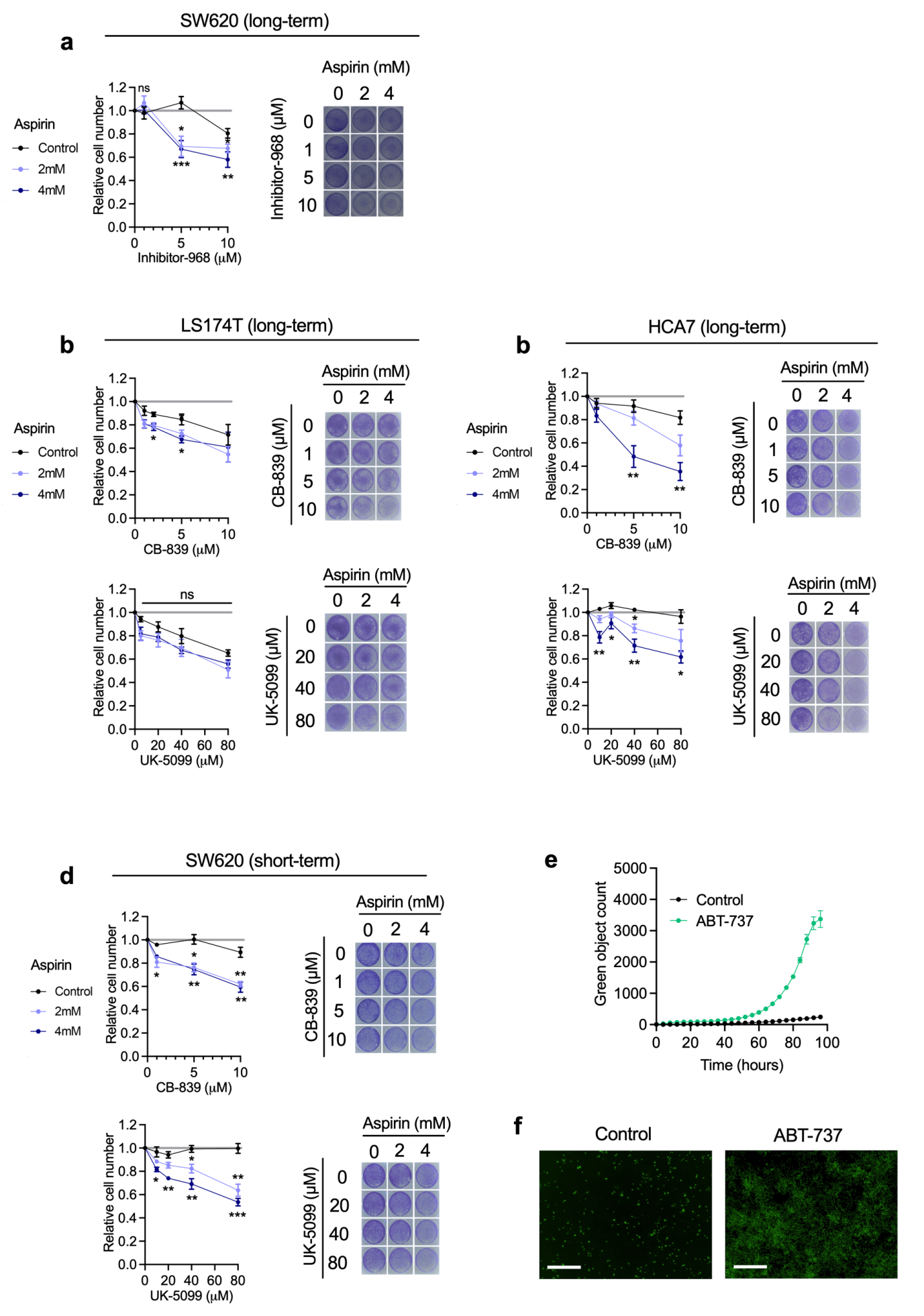
**

**Supplementary Figure 4. Aspirin sensitises CRC cells to metabolic inhibitors. a-d)** Cell proliferation assays of long-term (52 week) aspirin treated SW620 **(a)** LS174T **(b)** and HCA7 cells **(c)** and short-term (72 hour) aspirin treated SW620 cells **(d)** with increasing concentrations of either inhibitor 968, CB-839 or UK-5099. Graphs show relative cell number in each aspirin condition as measured by crystal violet staining at 72 hours of drug treatment compared to vehicle control. Error bars show SEM (n=5 independent experiments for HCA7 with CB-839, n=3 independent experiments for all other experiments). Asterisks refer to p-values obtained using one-way ANOVAs with Dunnett’s multiple comparisons tests at each drug concentration (*=p<0.05, **=p<0.01, ***=p<0.001). Images show representative wells in each condition after 72 hours of treatment. **e)** Quantification of green fluorescent nuclei indicating apoptotic SW620 cells with activated caspase-3/7, with 2µM ABT-737 treatment compared to control, as a positive control for apoptosis. Error bars represent SD (n=3 technical replicates). **f)** Representative images of 2µM ABT-737 treated and control SW620 cells at the end of the assay (~96 hours). Scale bar represents 300µm.


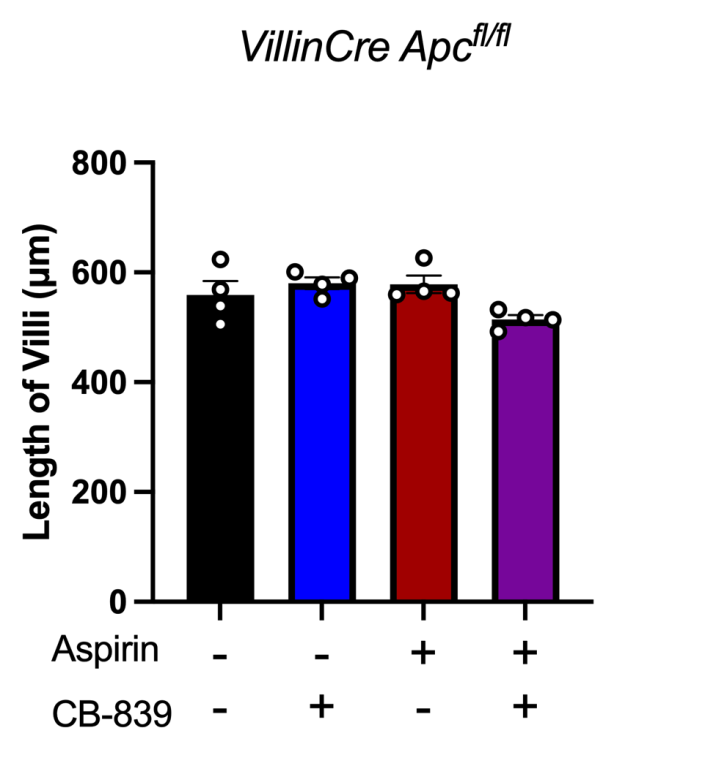


**Supplementary Figure 5. Aspirin and CB-389 in combination reduce colon crypt proliferation *in vivo.*** Quantification of the villi length in small intestine of *Villin^CreER^ Apc*^fl/fl^ mice treated with CB-839 (200mg/kg) and/or aspirin (2.6mg/ml in drinking water). Error bars show SEM (n=4 mice per experimental arm). Each dot represents the average length of villi for each mouse. Statistical analysis (ns, p > 0.05), one-way ANOVA with Tukey or Bonferroni post hoc test.
