# Supplementary material for: Aspirin reprogrammes colorectal cancer cell metabolism and sensitises to glutaminase inhibition
Source: Cancer Metab. 2023 Oct 19;11:18. doi: 10.1186/s40170-023-00318-y (PMC10588174; doi:10.1186/s40170-023-00318-y)
Supplement: Supplementary file 2 — Additional file 2: Table S1. Metabolomics Data. Table S2. Proteomics Data. Table S3. Uncropped western scans [file 40170_2023_318_MOESM2_ESM.zip › Holt et al_Uncropped western scans.pptx]

## Slide 1
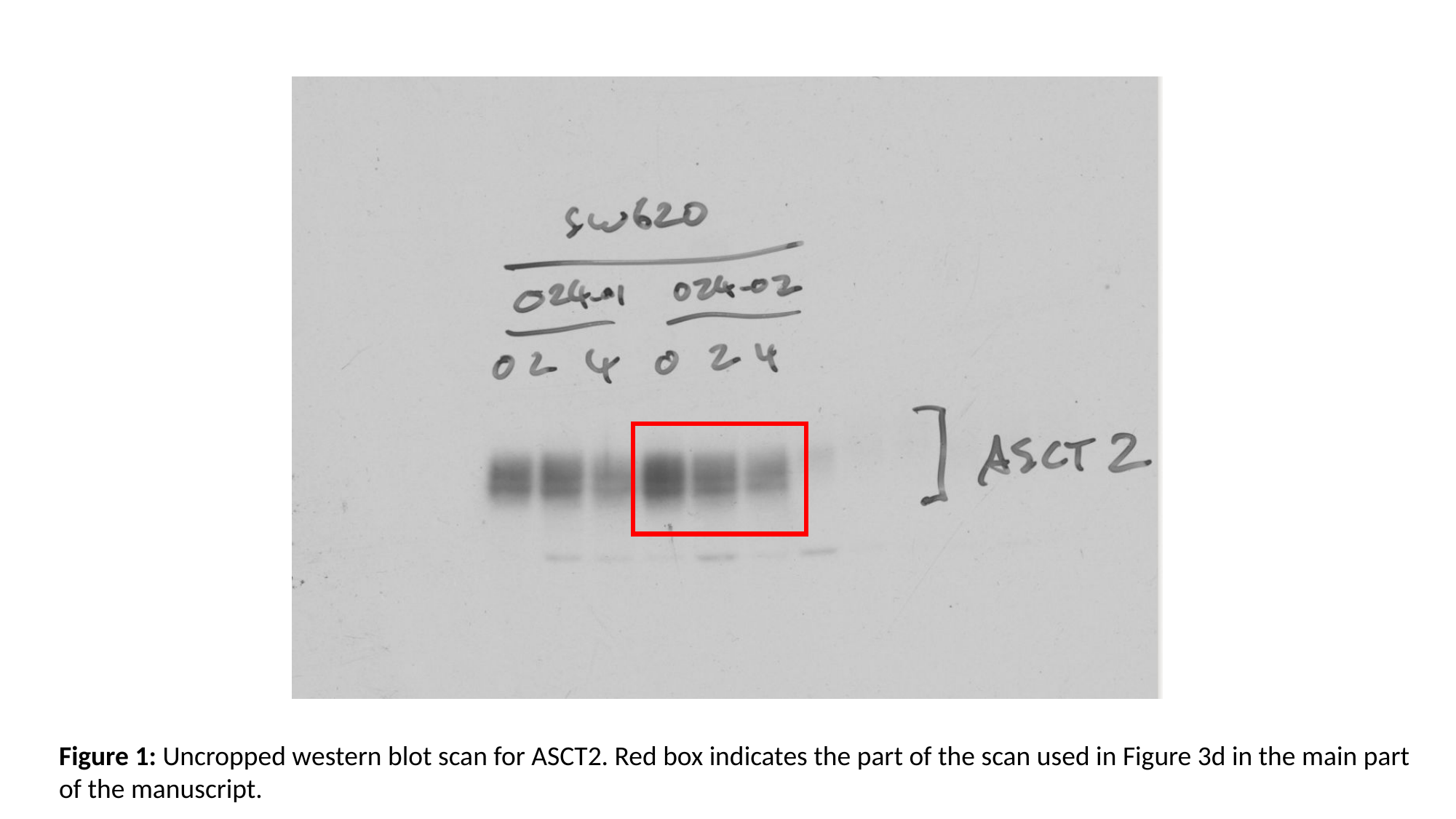

Figure 1: Uncropped western blot scan for ASCT2. Red box indicates the part of the scan used in Figure 3d in the main part of the manuscript.

## Slide 2
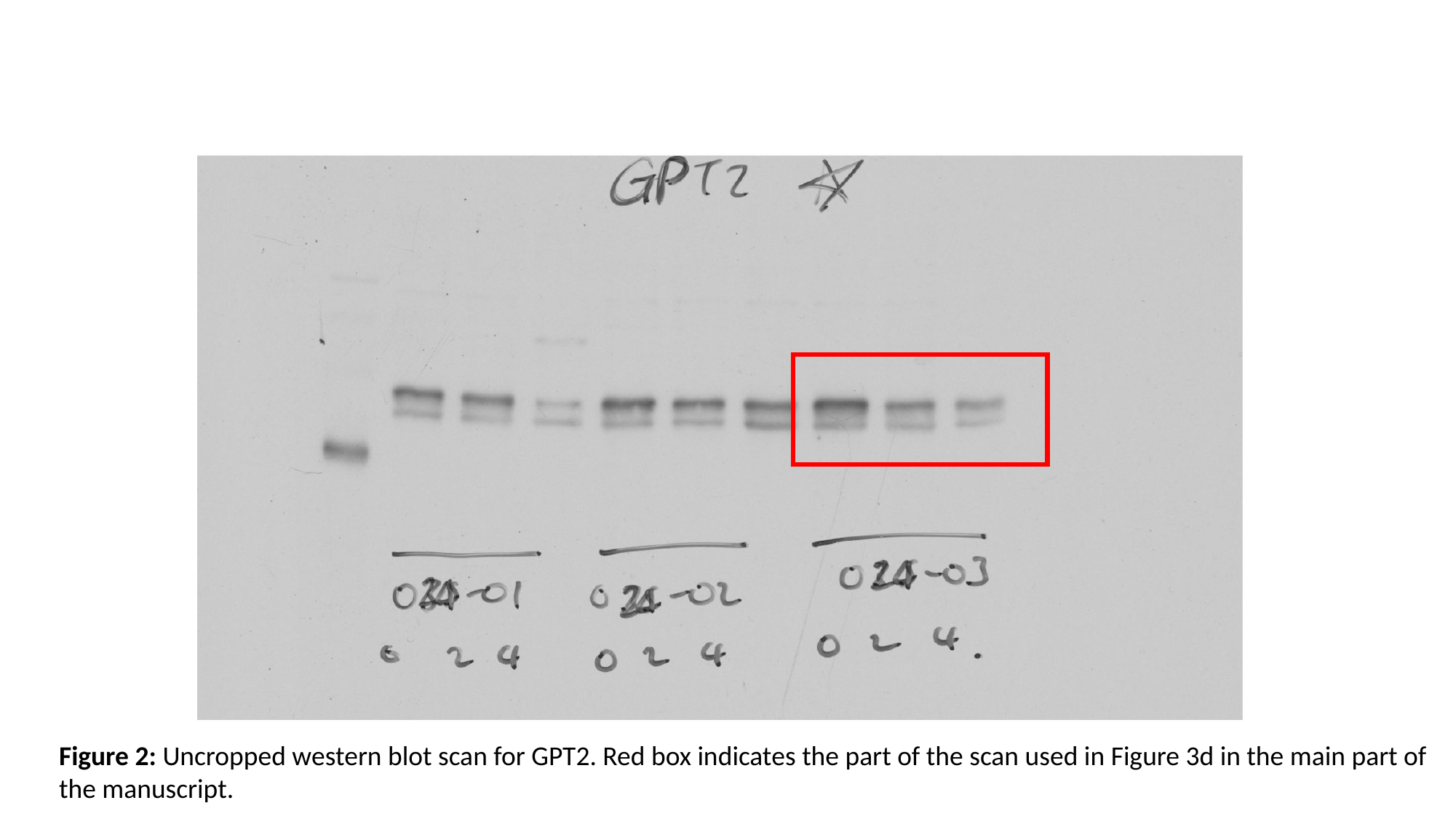

Figure 2: Uncropped western blot scan for GPT2. Red box indicates the part of the scan used in Figure 3d in the main part of the manuscript.

## Slide 3
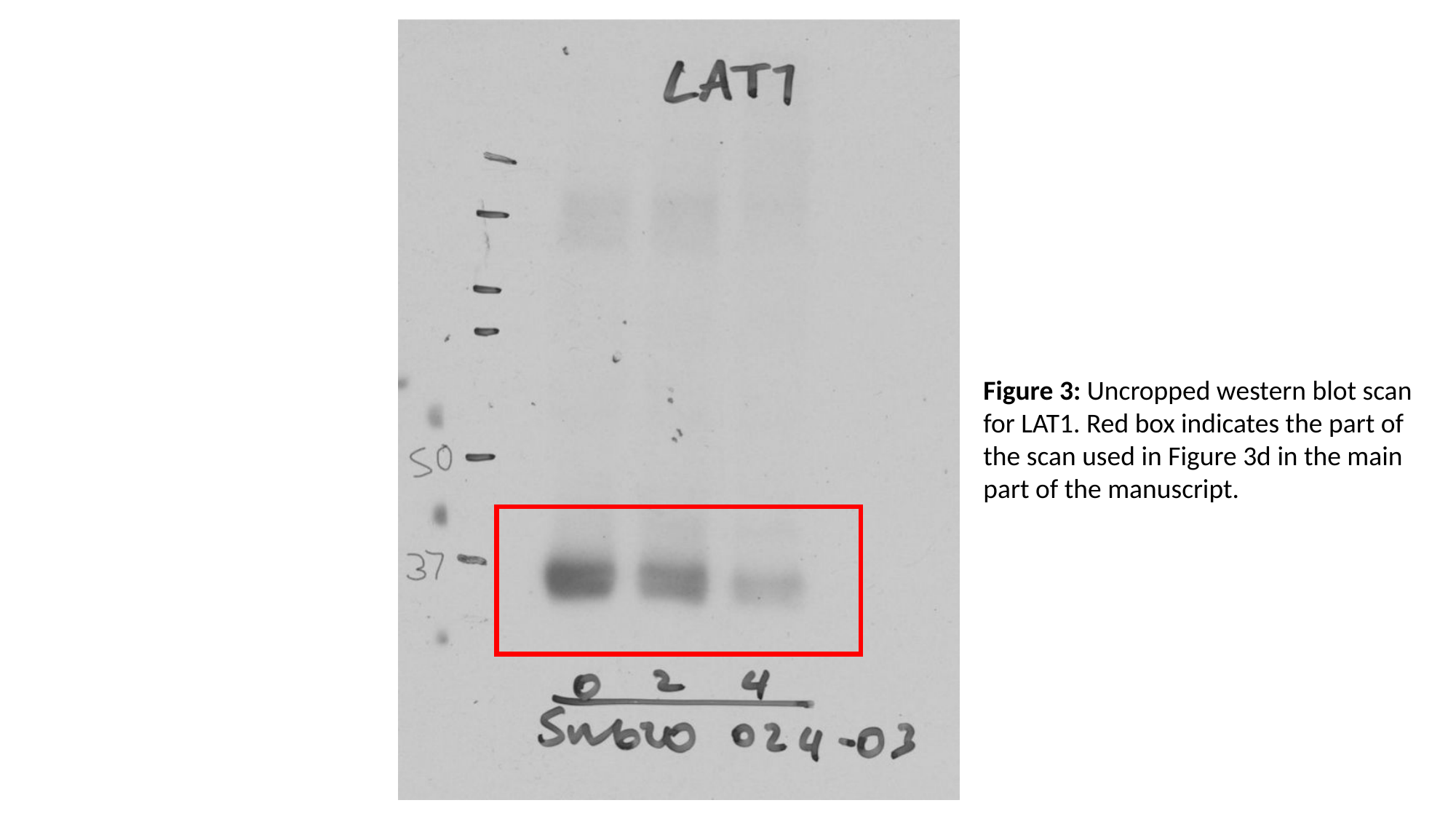

Figure 3: Uncropped western blot scan for LAT1. Red box indicates the part of the scan used in Figure 3d in the main part of the manuscript.

## Slide 4
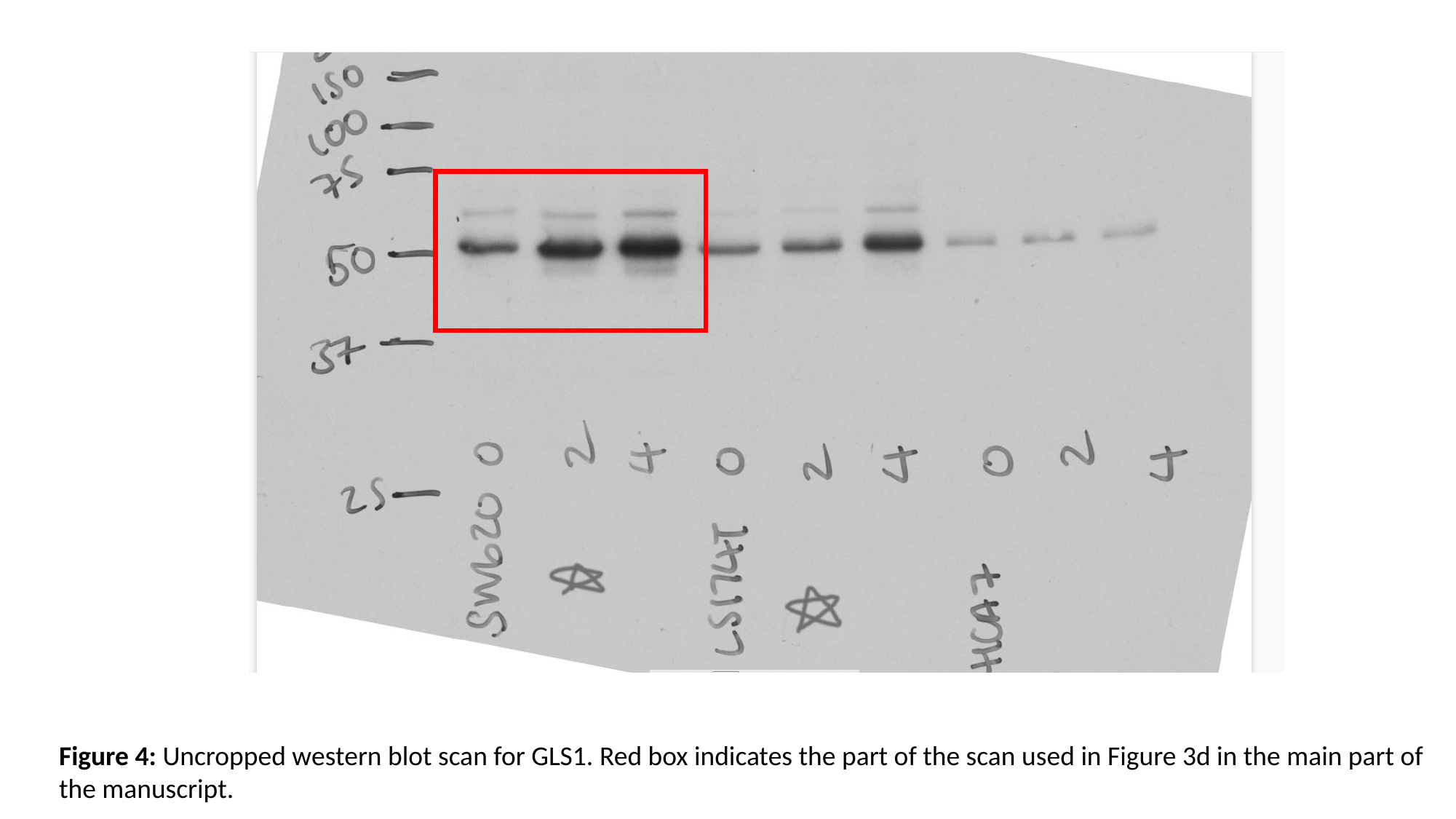

Figure 4: Uncropped western blot scan for GLS1. Red box indicates the part of the scan used in Figure 3d in the main part of the manuscript.

## Slide 5
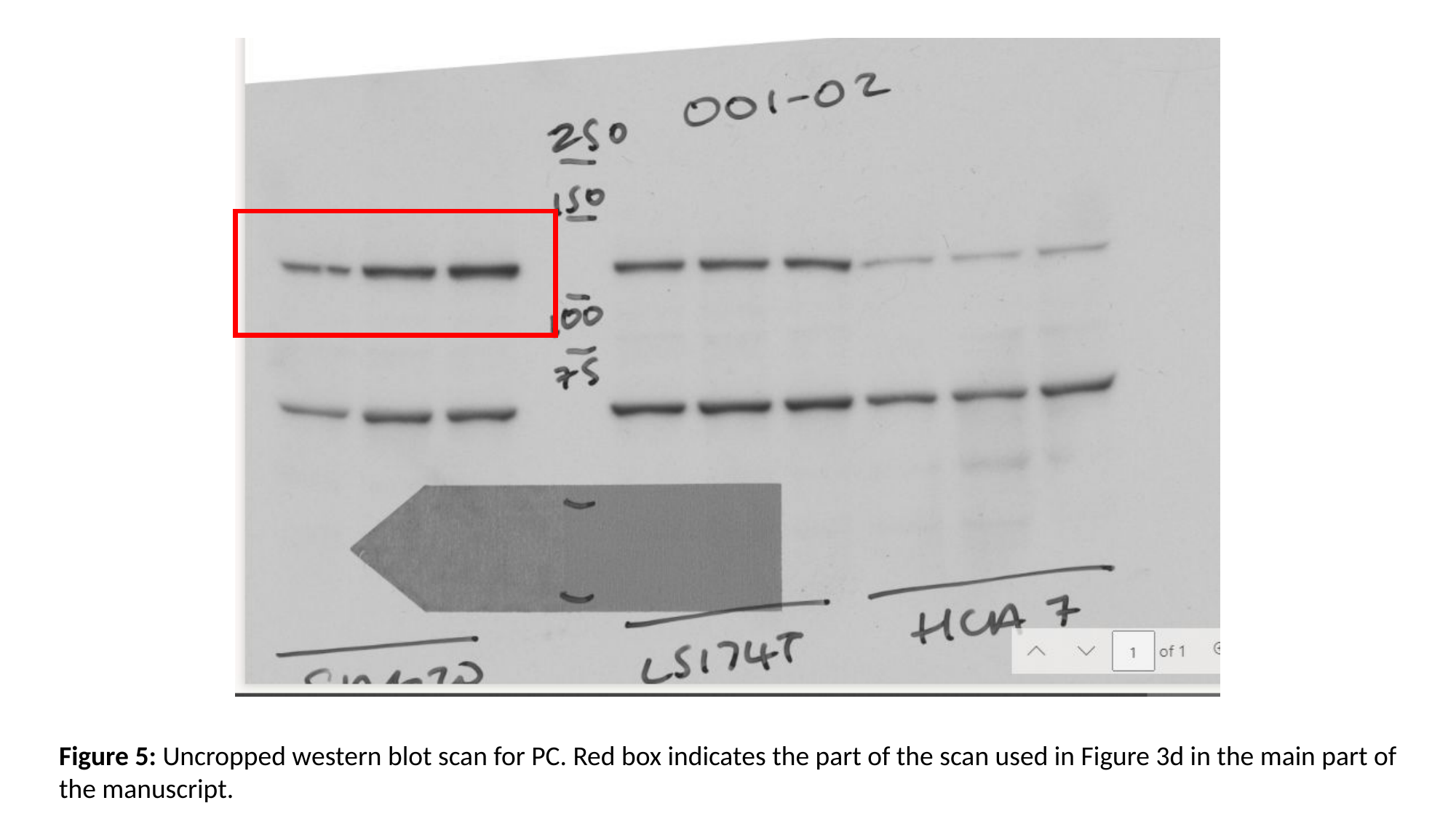

Figure 5: Uncropped western blot scan for PC. Red box indicates the part of the scan used in Figure 3d in the main part of the manuscript.

## Slide 6
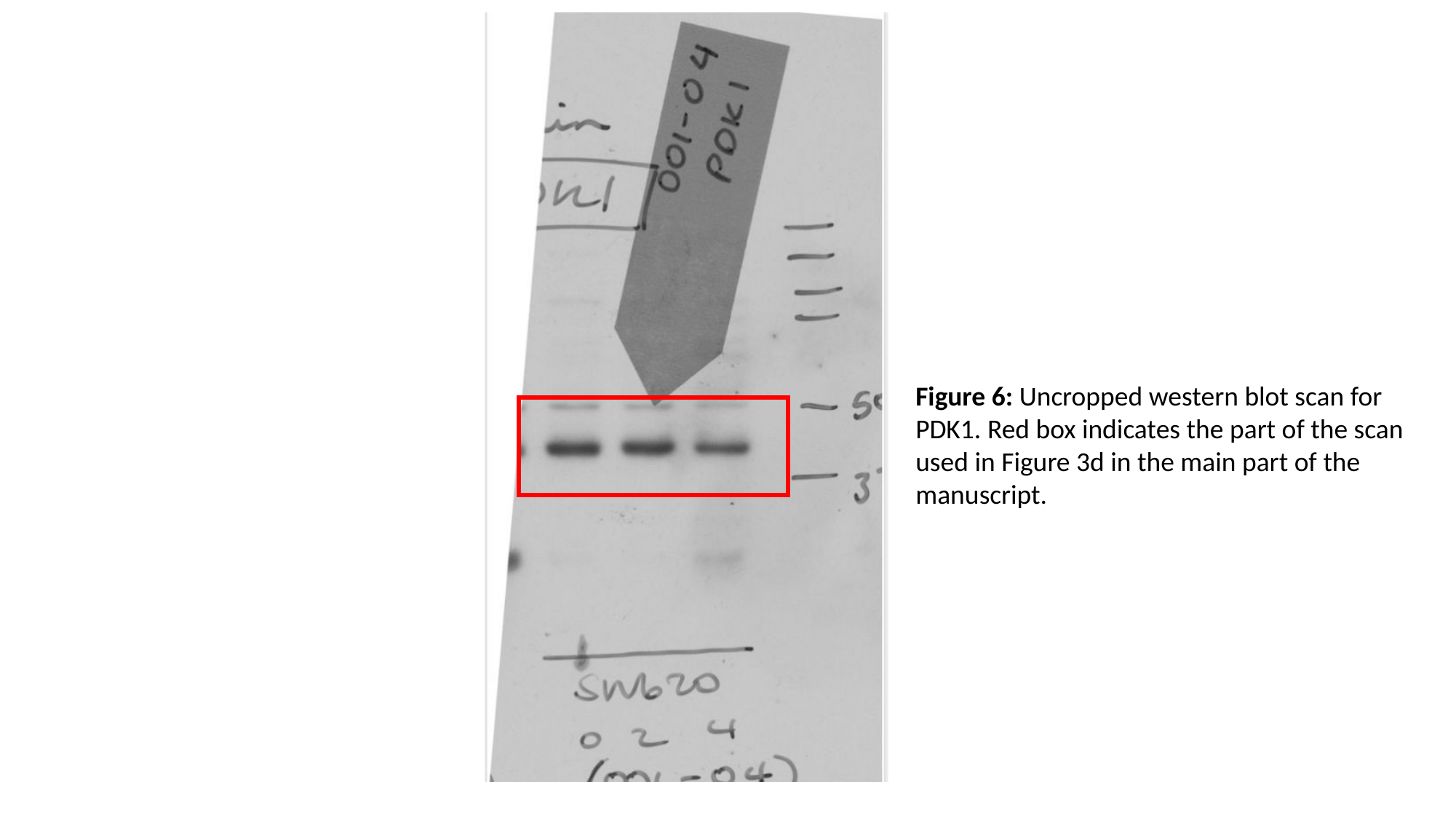

Figure 6: Uncropped western blot scan for PDK1. Red box indicates the part of the scan used in Figure 3d in the main part of the manuscript.

## Slide 7
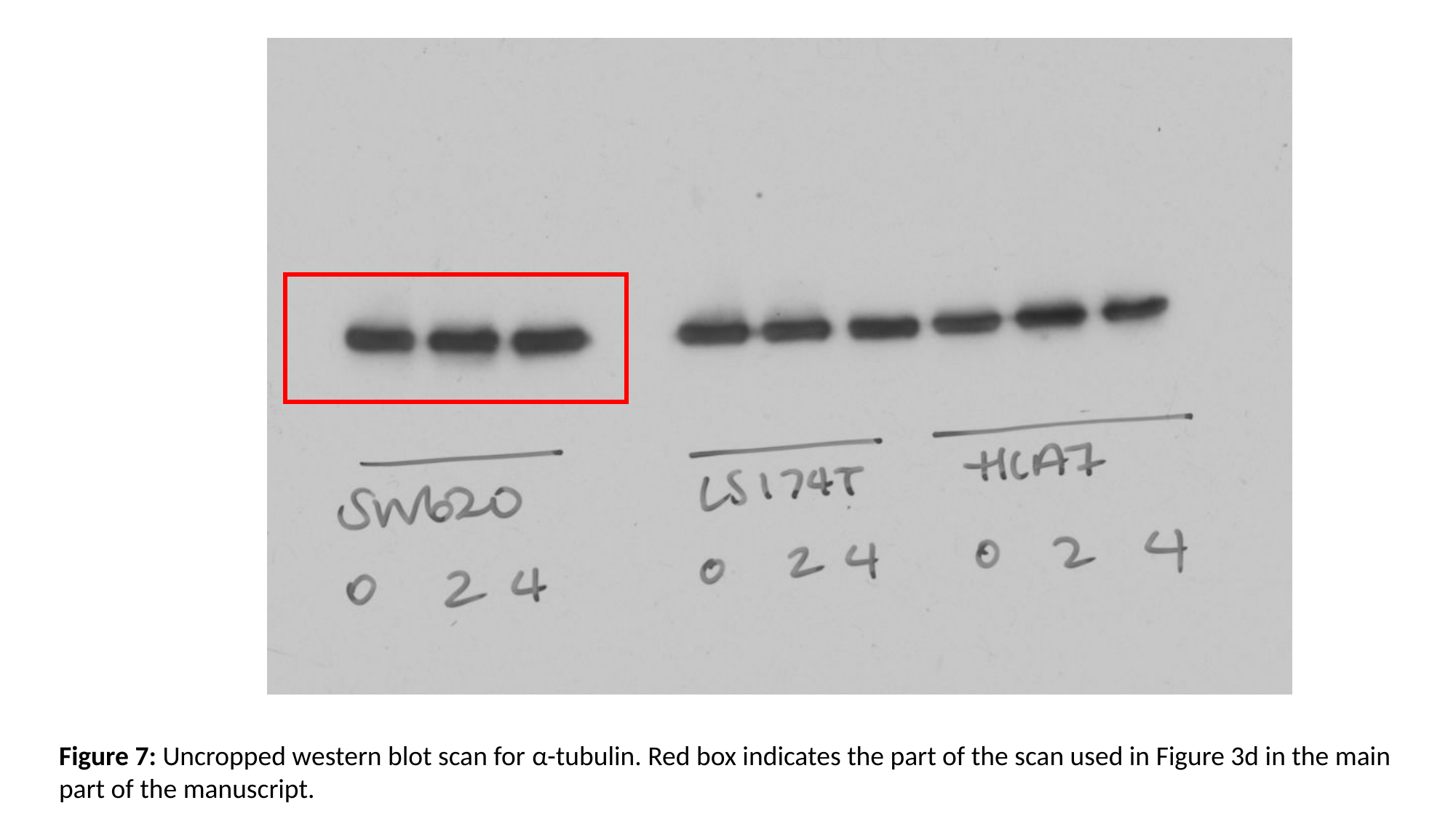

Figure 7: Uncropped western blot scan for α-tubulin. Red box indicates the part of the scan used in Figure 3d in the main part of the manuscript.

## Slide 8
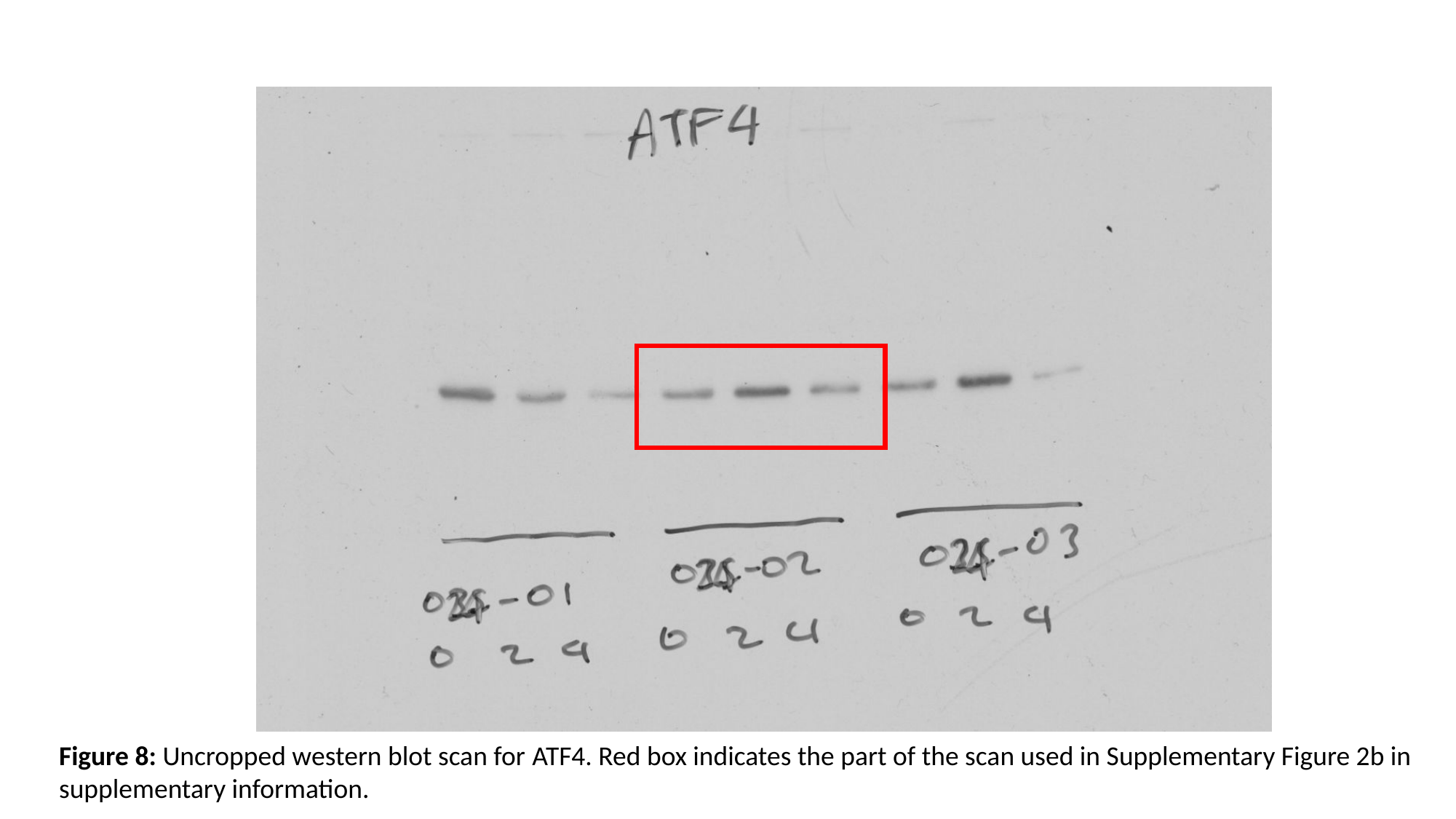

Figure 8: Uncropped western blot scan for ATF4. Red box indicates the part of the scan used in Supplementary Figure 2b in supplementary information.

## Slide 9
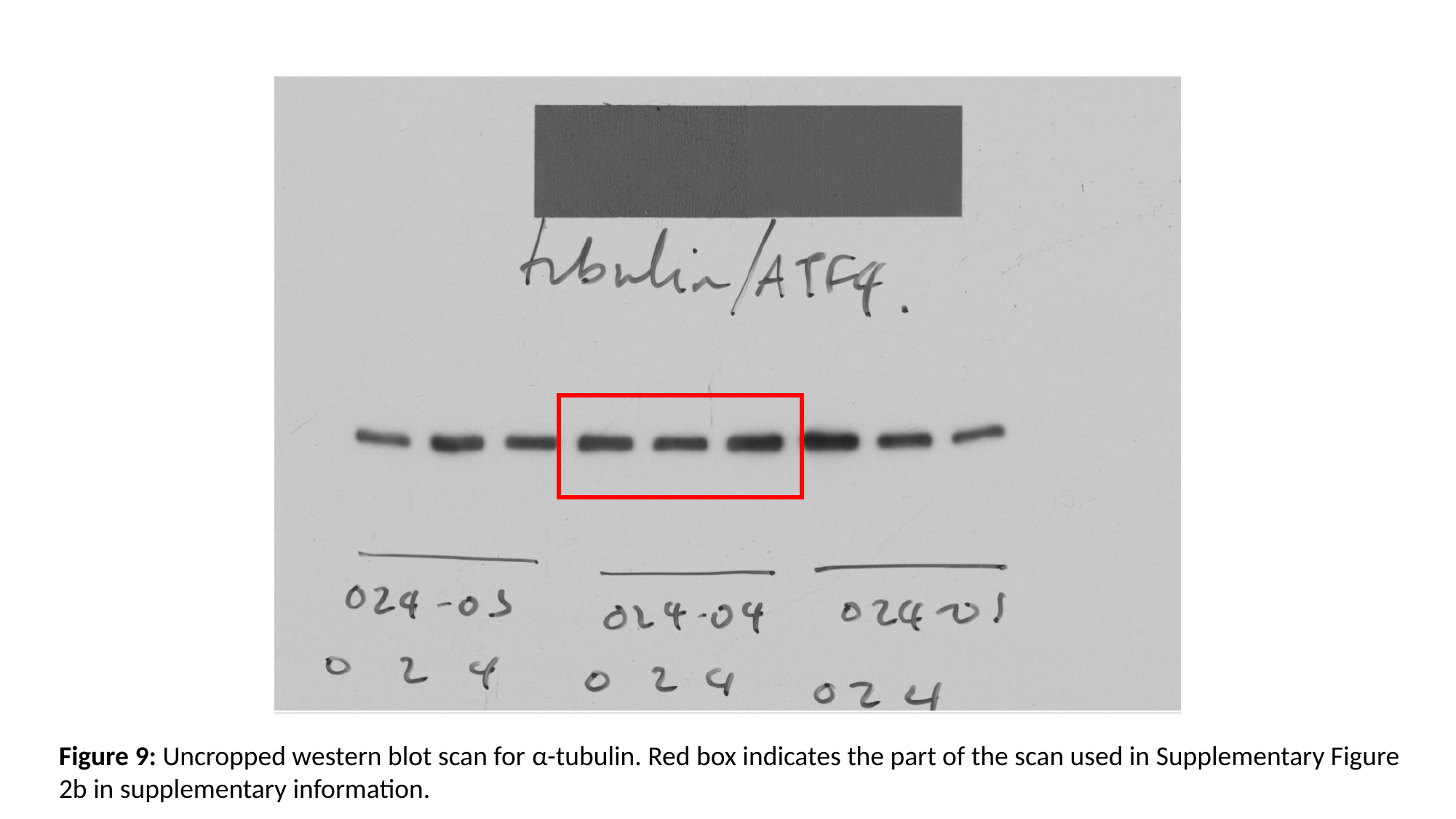

Figure 9: Uncropped western blot scan for α-tubulin. Red box indicates the part of the scan used in Supplementary Figure 2b in supplementary information.

## Slide 10
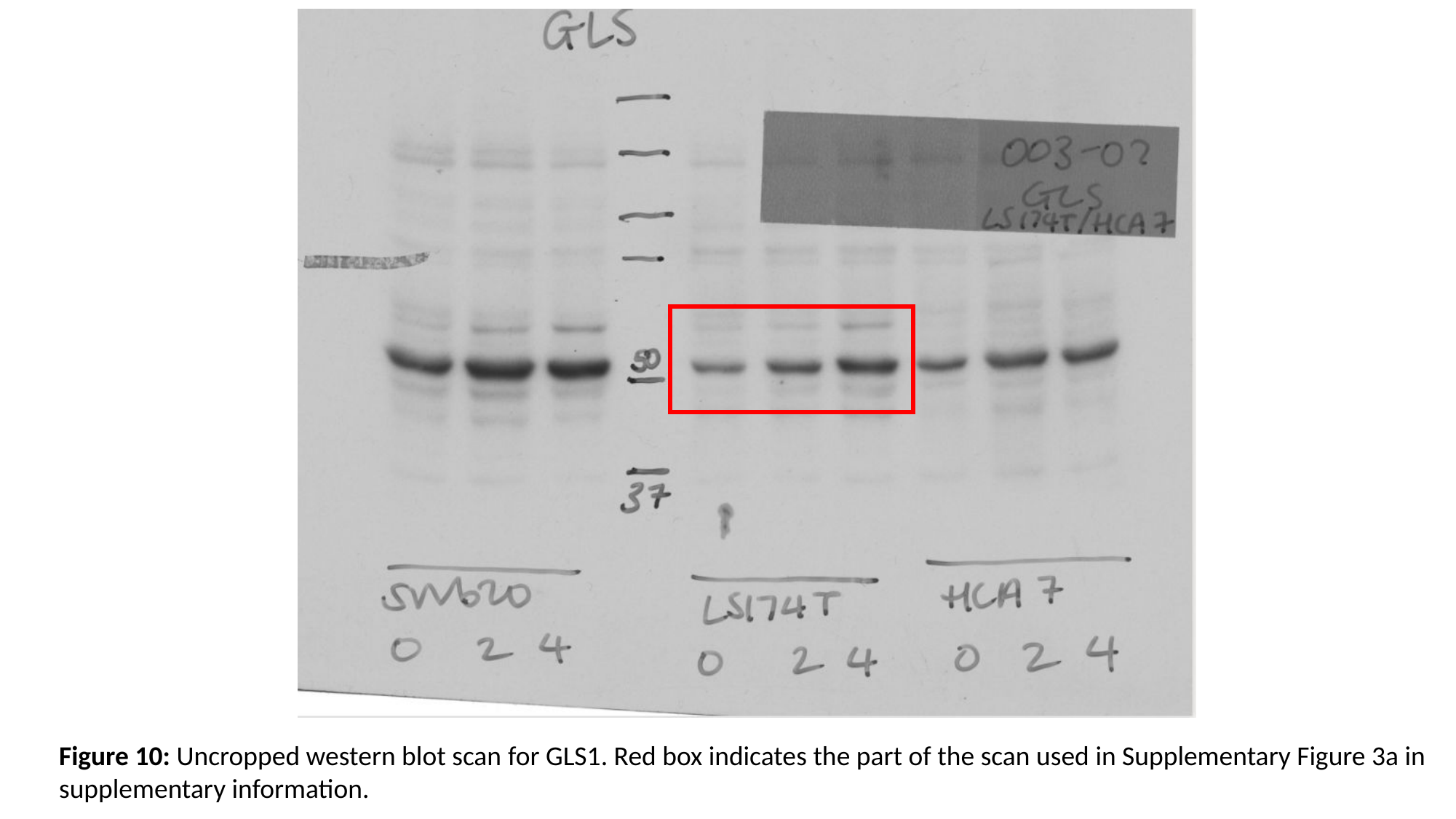

Figure 10: Uncropped western blot scan for GLS1. Red box indicates the part of the scan used in Supplementary Figure 3a in supplementary information.

## Slide 11
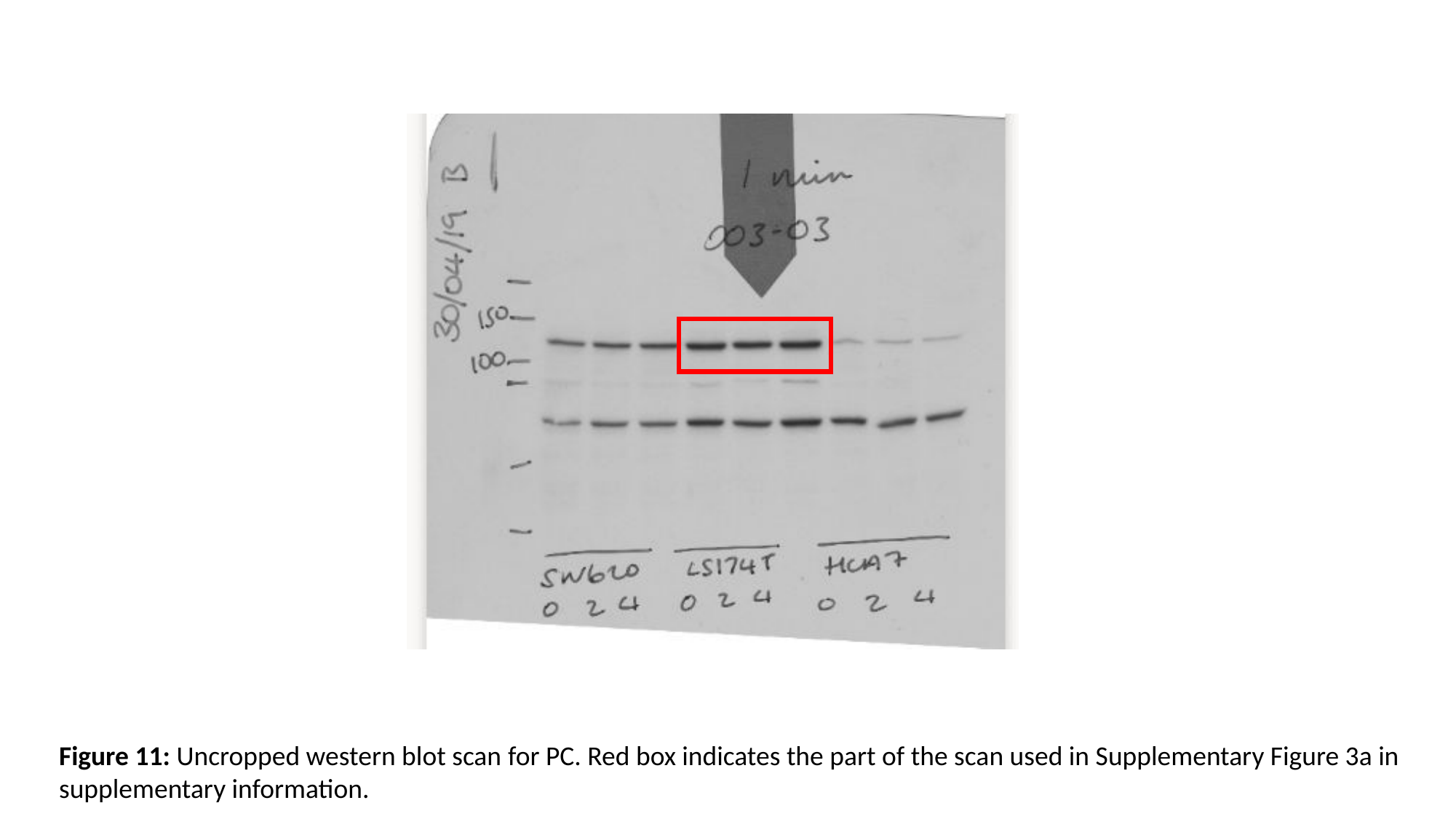

Figure 11: Uncropped western blot scan for PC. Red box indicates the part of the scan used in Supplementary Figure 3a in supplementary information.

## Slide 12
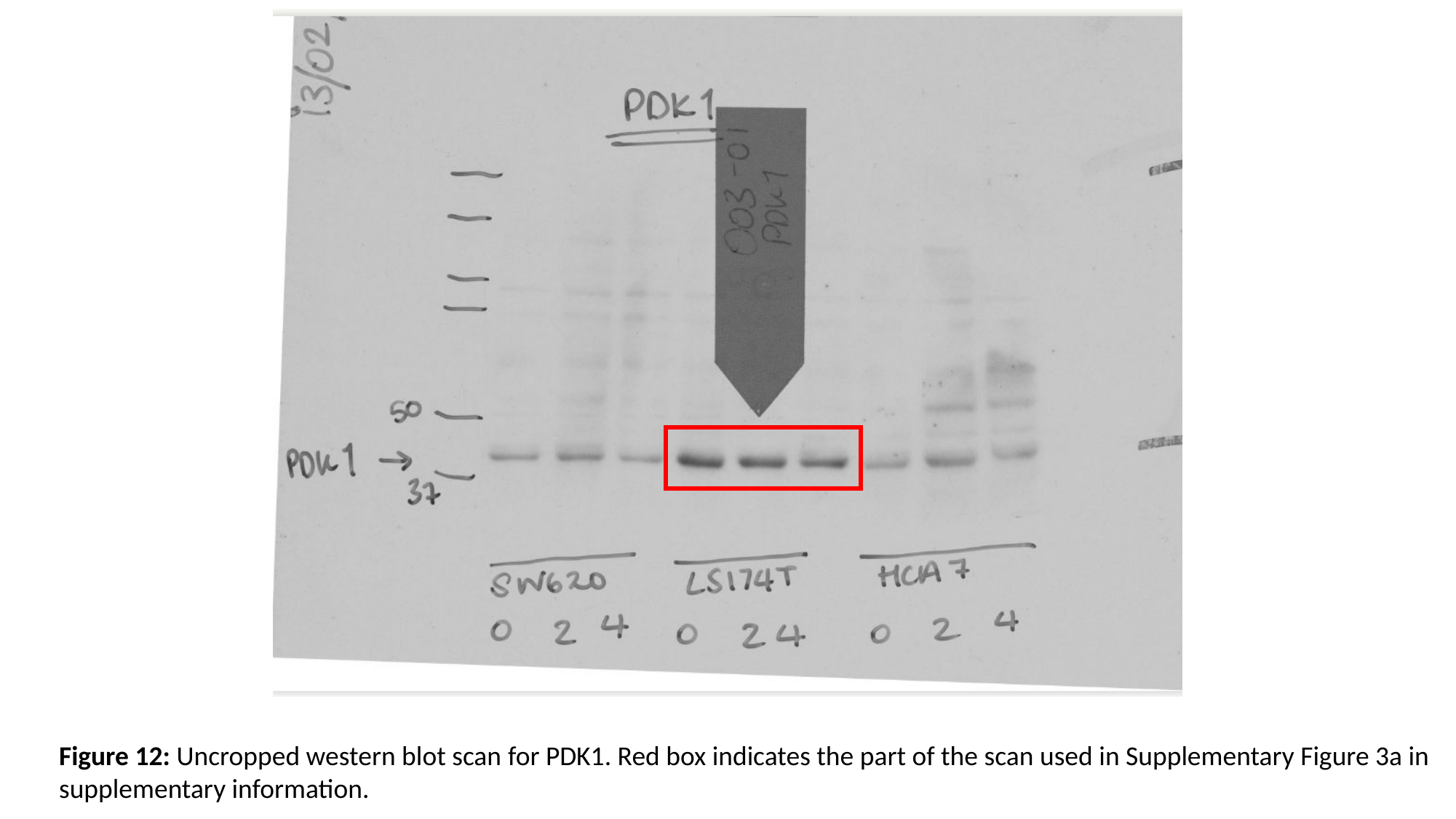

Figure 12: Uncropped western blot scan for PDK1. Red box indicates the part of the scan used in Supplementary Figure 3a in supplementary information.

## Slide 13
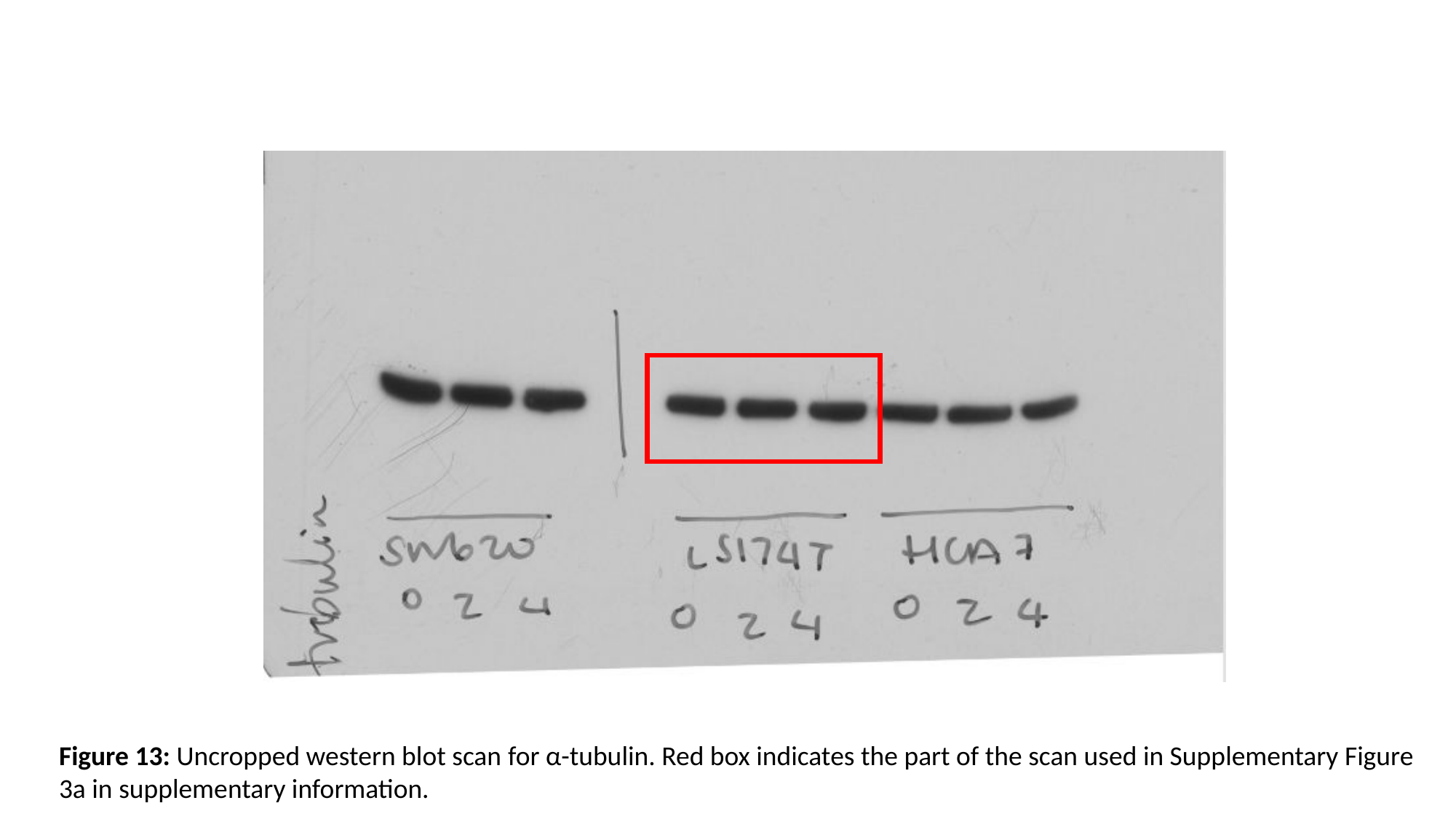

Figure 13: Uncropped western blot scan for α-tubulin. Red box indicates the part of the scan used in Supplementary Figure 3a in supplementary information.

## Slide 14
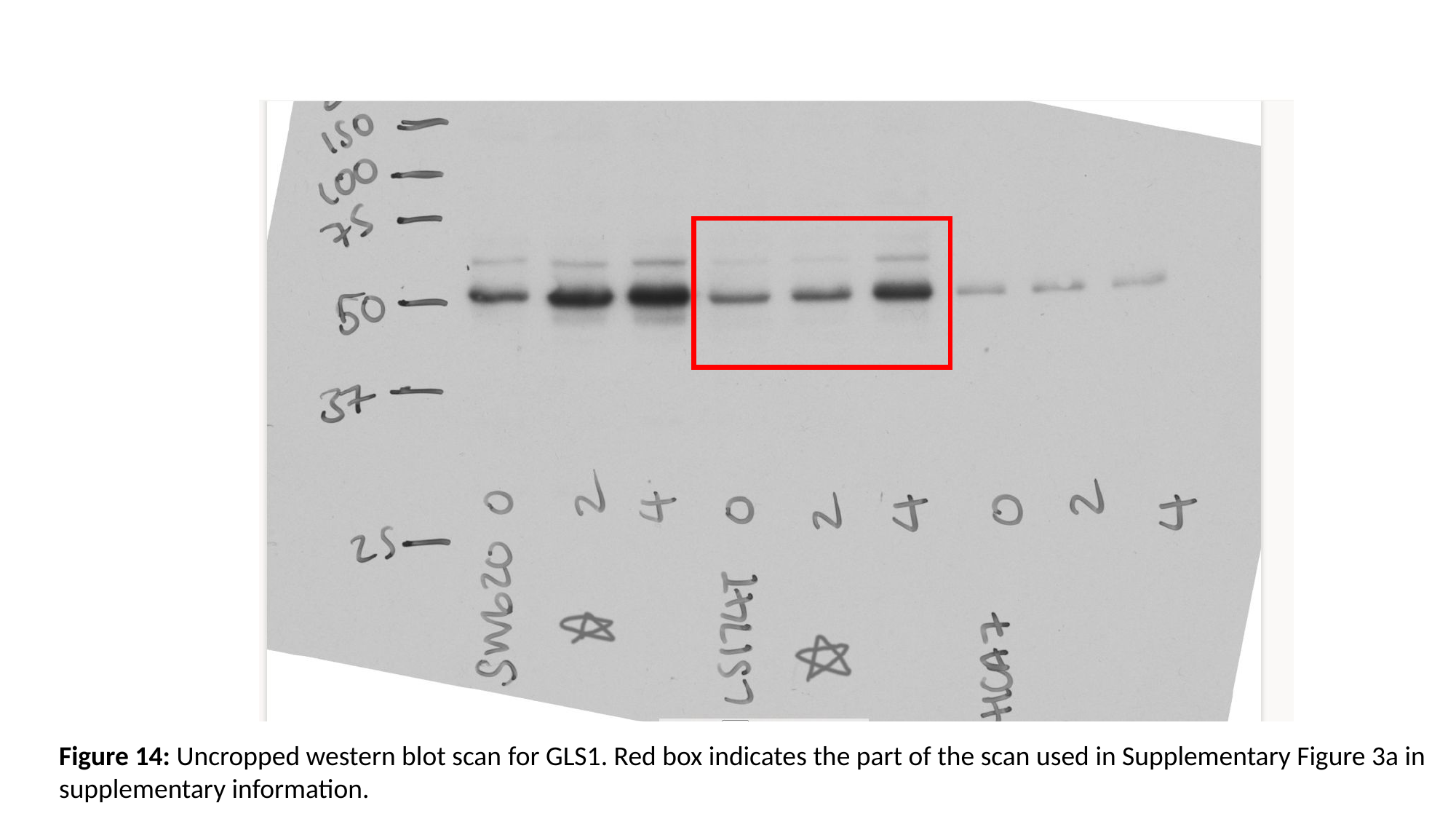

Figure 14: Uncropped western blot scan for GLS1. Red box indicates the part of the scan used in Supplementary Figure 3a in supplementary information.

## Slide 15
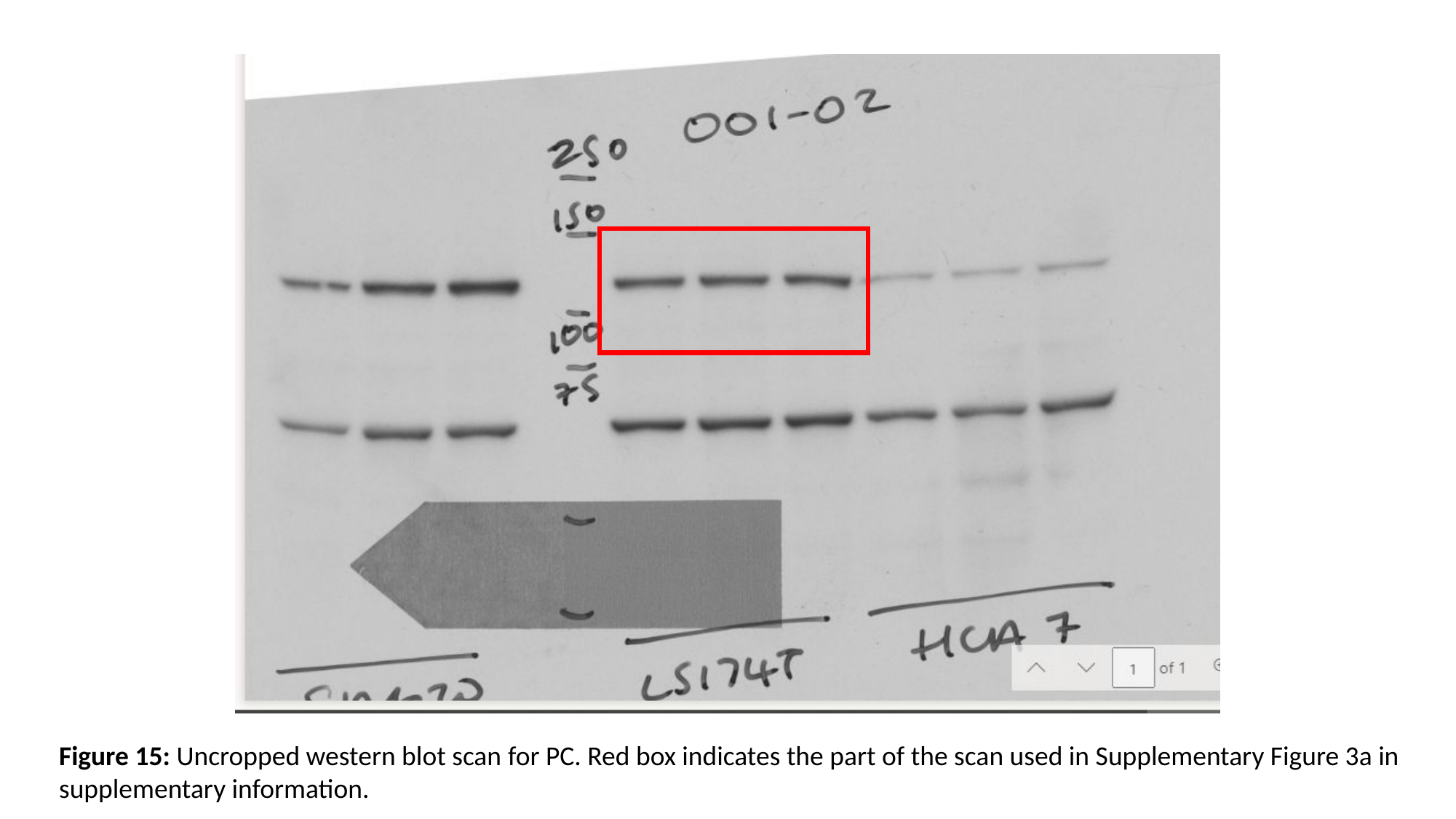

Figure 15: Uncropped western blot scan for PC. Red box indicates the part of the scan used in Supplementary Figure 3a in supplementary information.

## Slide 16
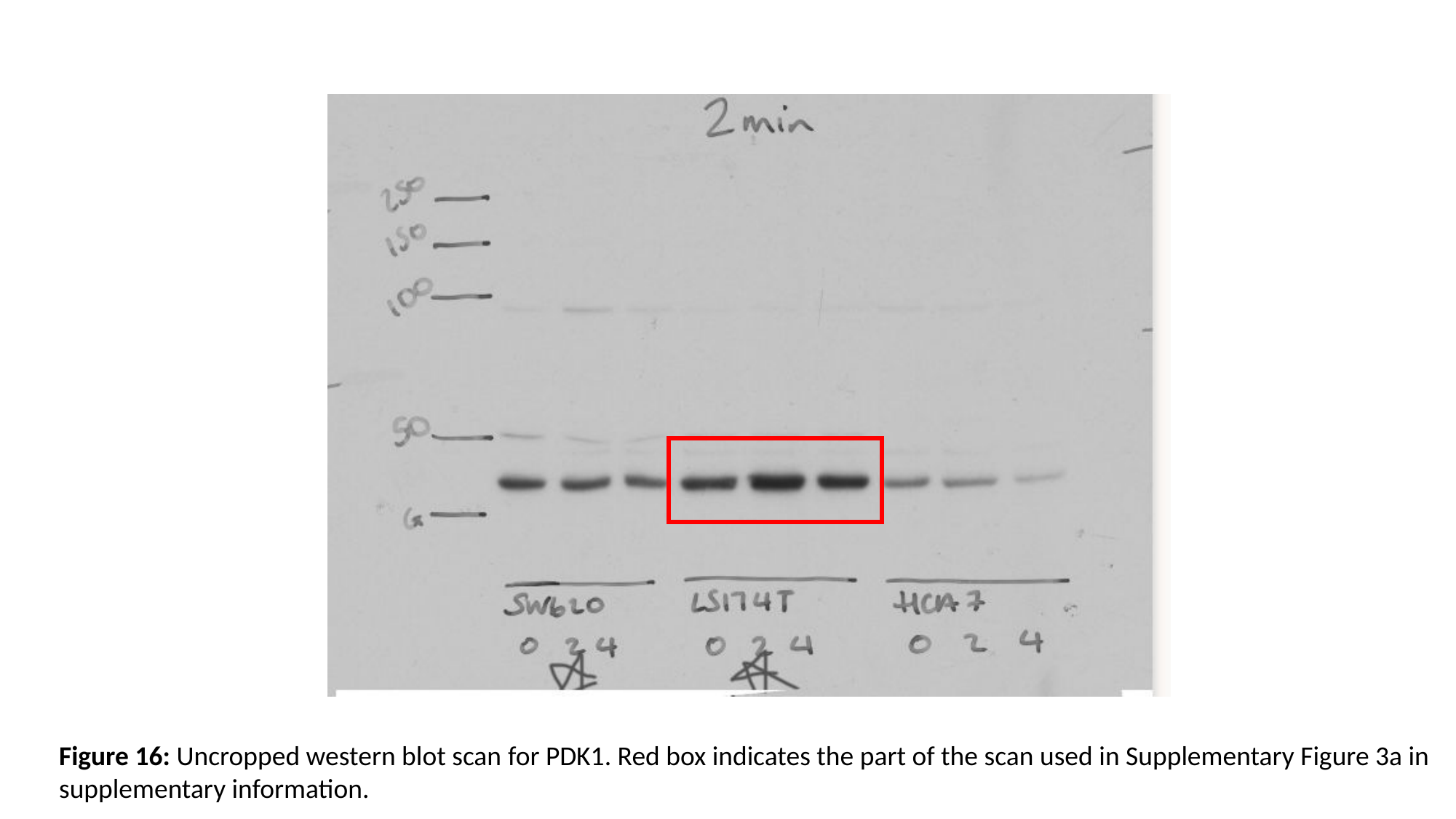

Figure 16: Uncropped western blot scan for PDK1. Red box indicates the part of the scan used in Supplementary Figure 3a in supplementary information.

## Slide 17
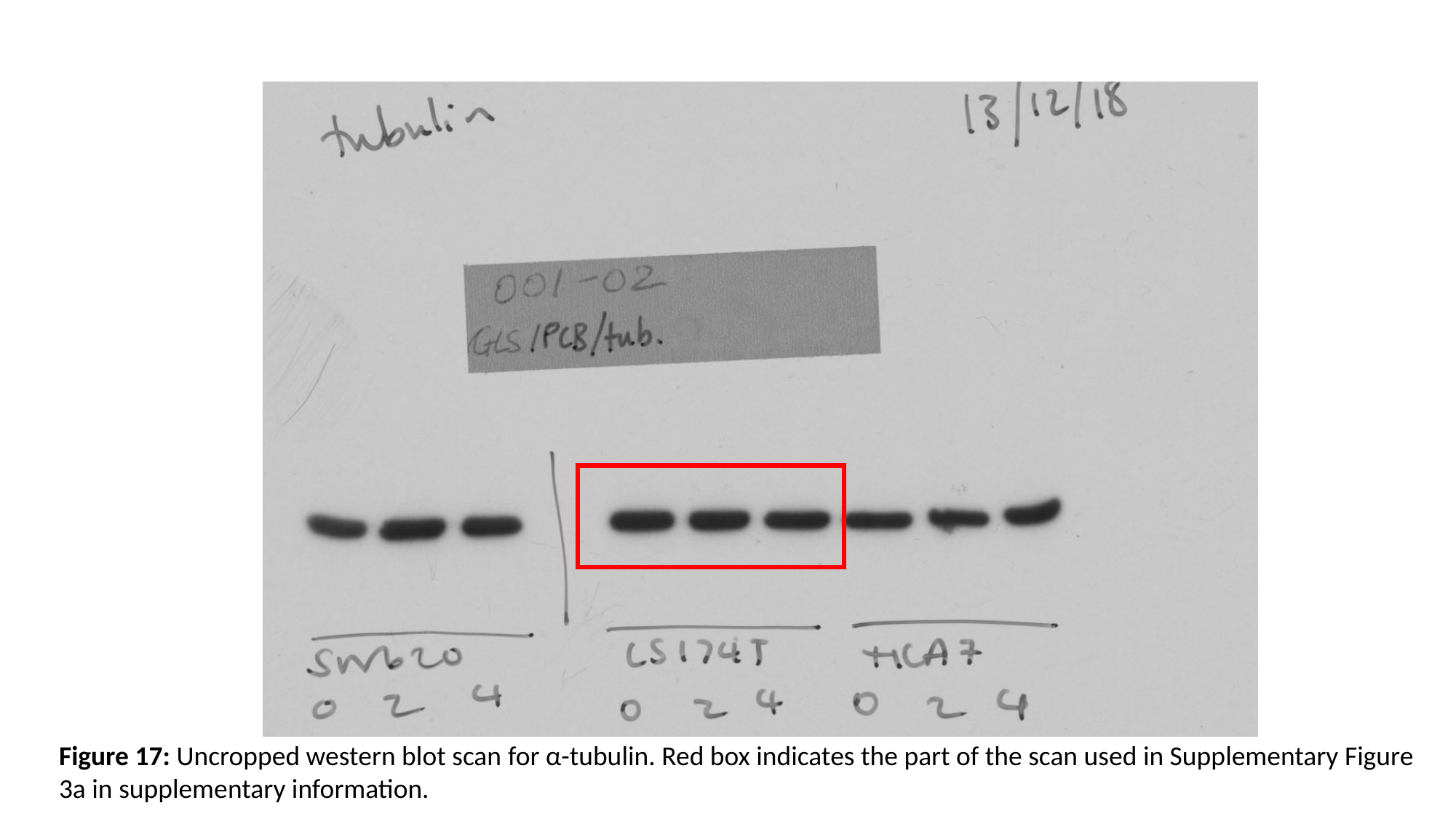

Figure 17: Uncropped western blot scan for α-tubulin. Red box indicates the part of the scan used in Supplementary Figure 3a in supplementary information.

## Slide 18
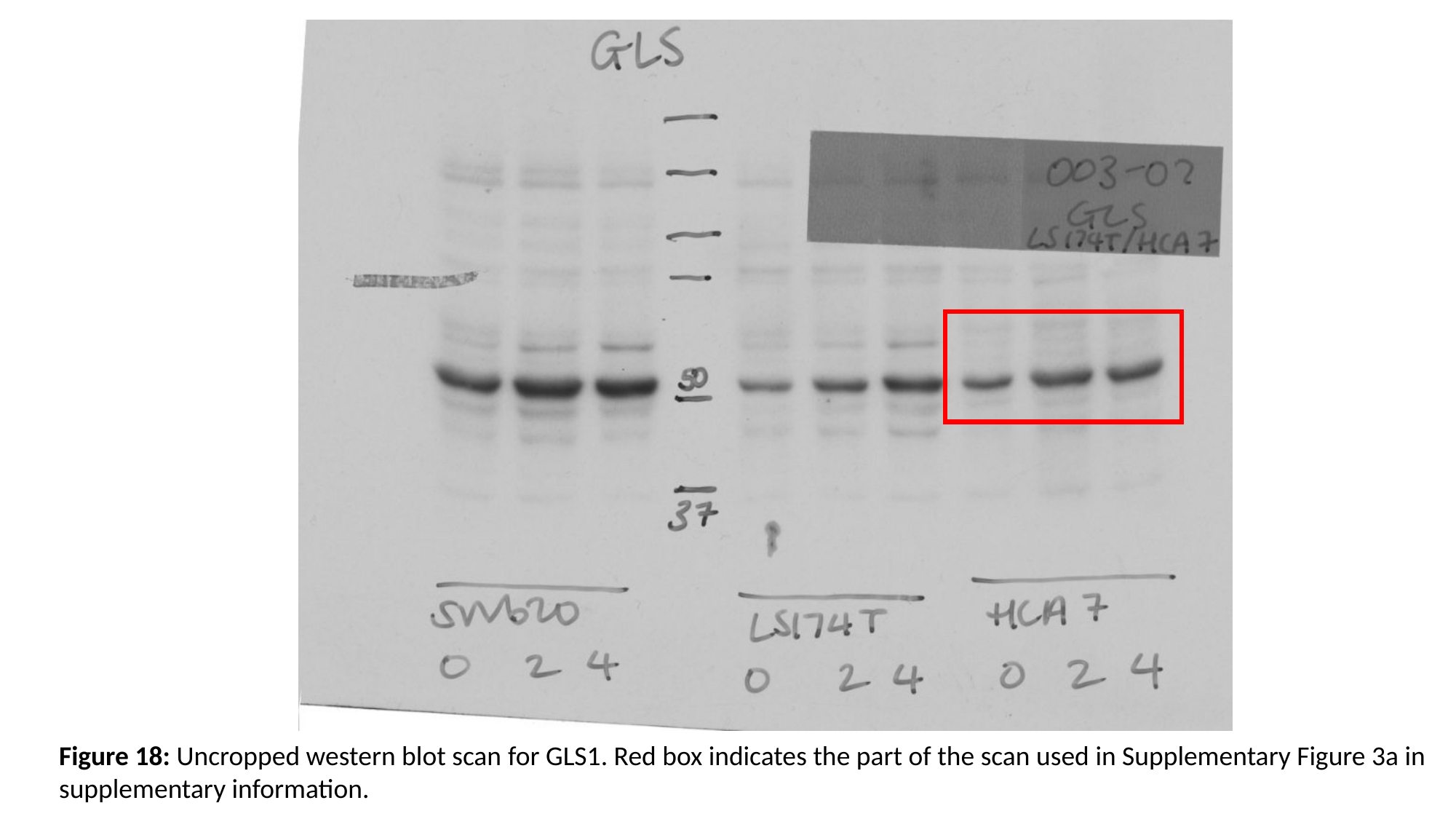

Figure 18: Uncropped western blot scan for GLS1. Red box indicates the part of the scan used in Supplementary Figure 3a in supplementary information.

## Slide 19
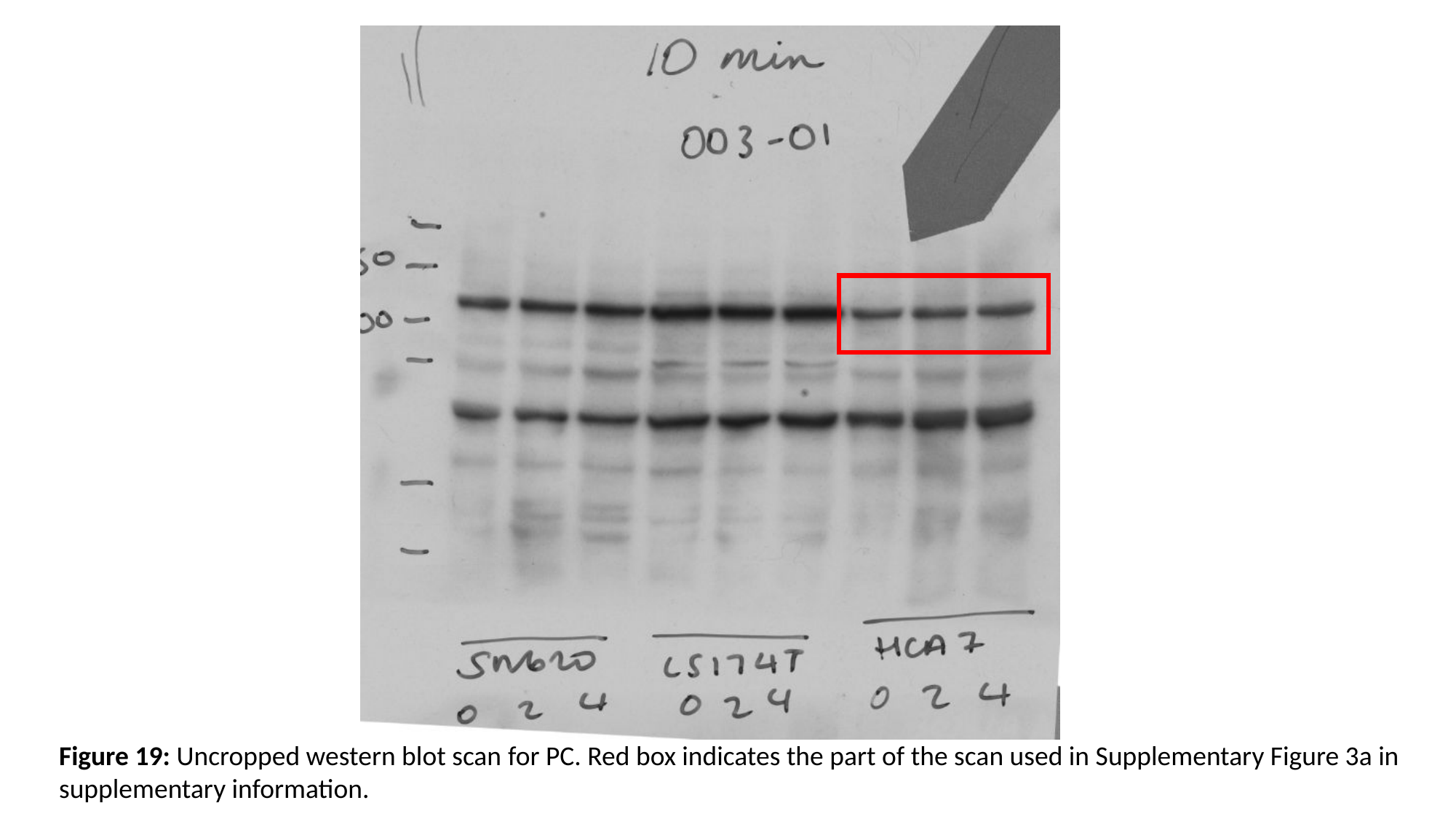

Figure 19: Uncropped western blot scan for PC. Red box indicates the part of the scan used in Supplementary Figure 3a in supplementary information.

## Slide 20
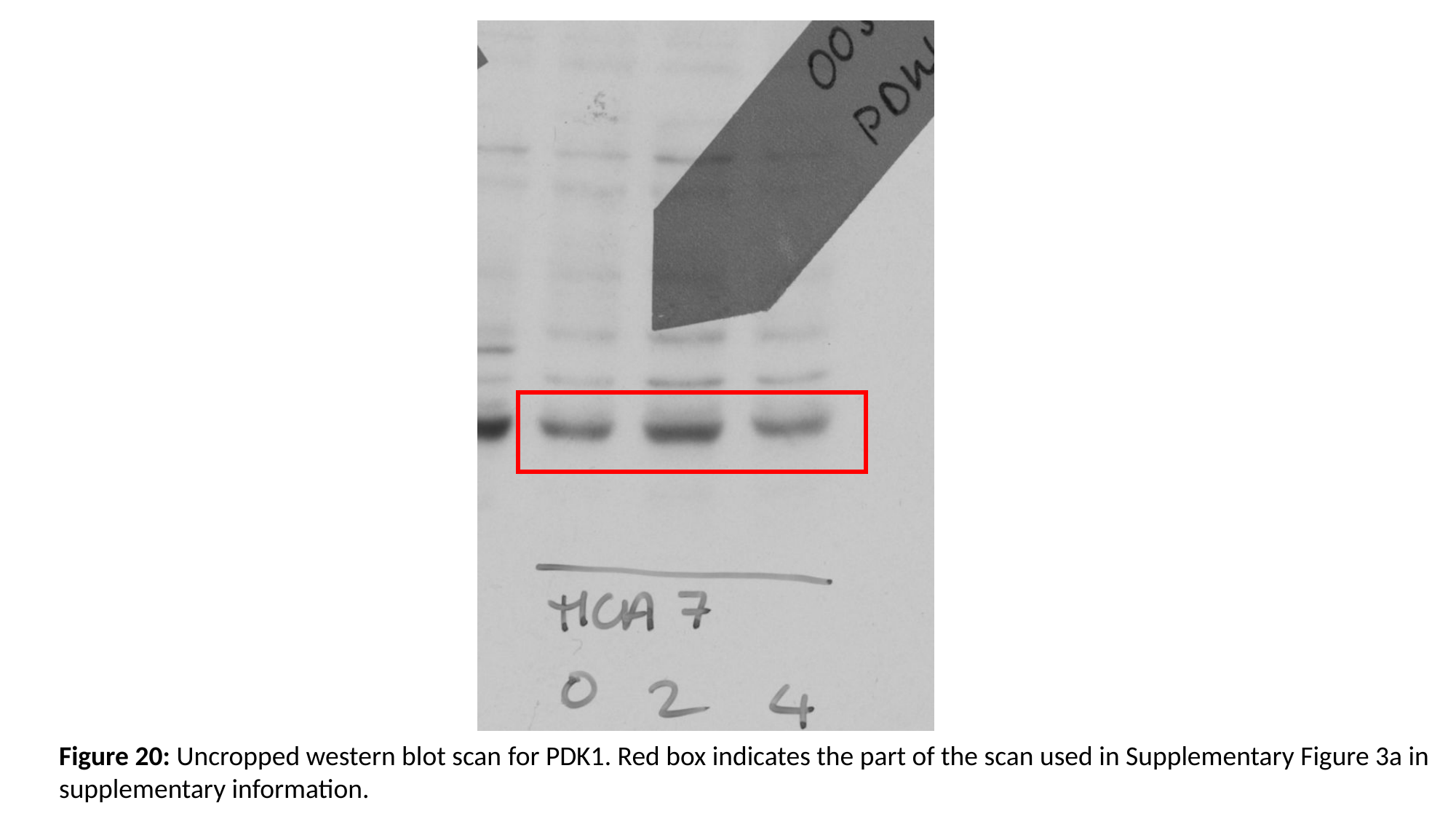

Figure 20: Uncropped western blot scan for PDK1. Red box indicates the part of the scan used in Supplementary Figure 3a in supplementary information.

## Slide 21
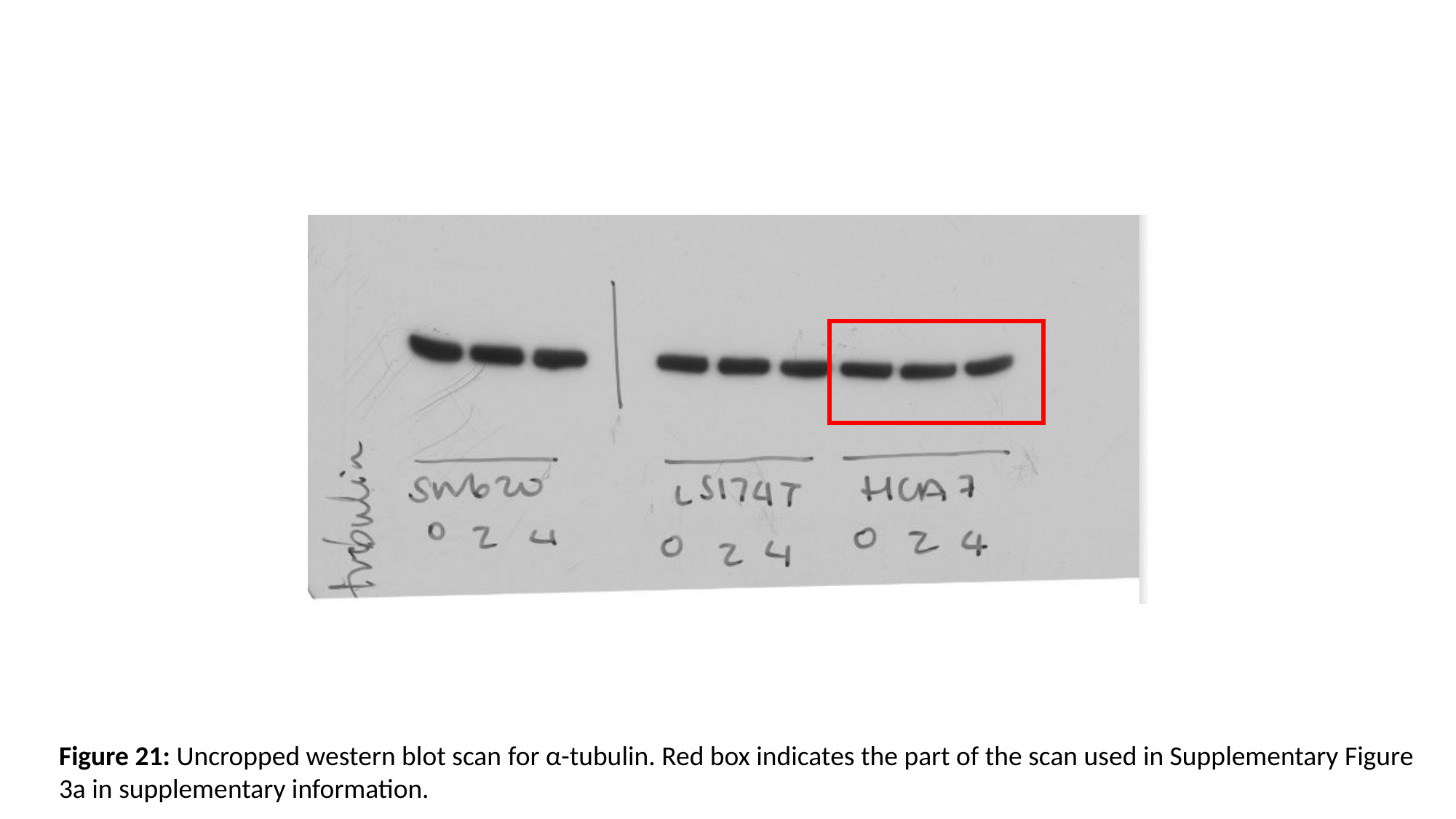

Figure 21: Uncropped western blot scan for α-tubulin. Red box indicates the part of the scan used in Supplementary Figure 3a in supplementary information.

## Slide 22
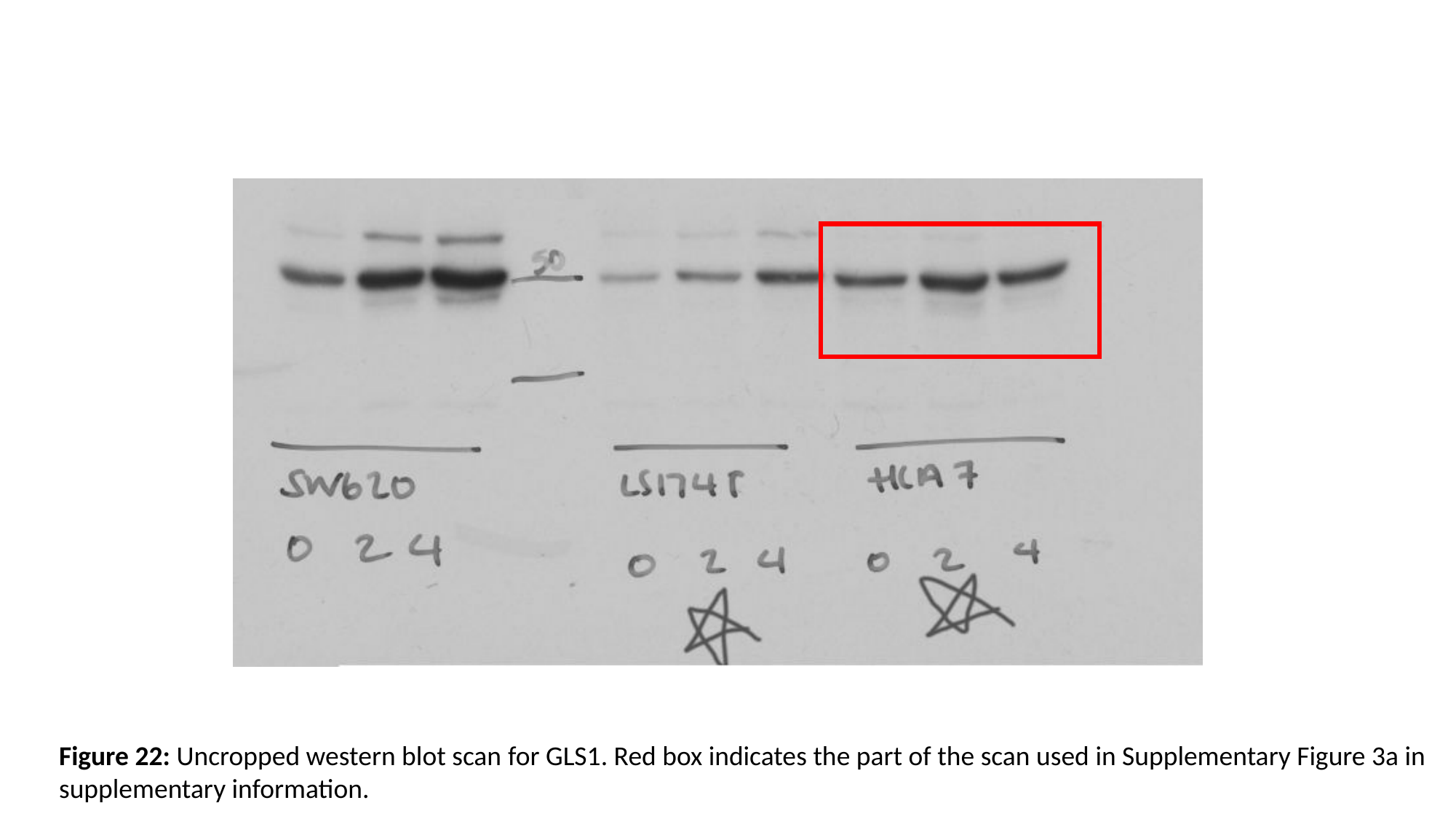

Figure 22: Uncropped western blot scan for GLS1. Red box indicates the part of the scan used in Supplementary Figure 3a in supplementary information.

## Slide 23
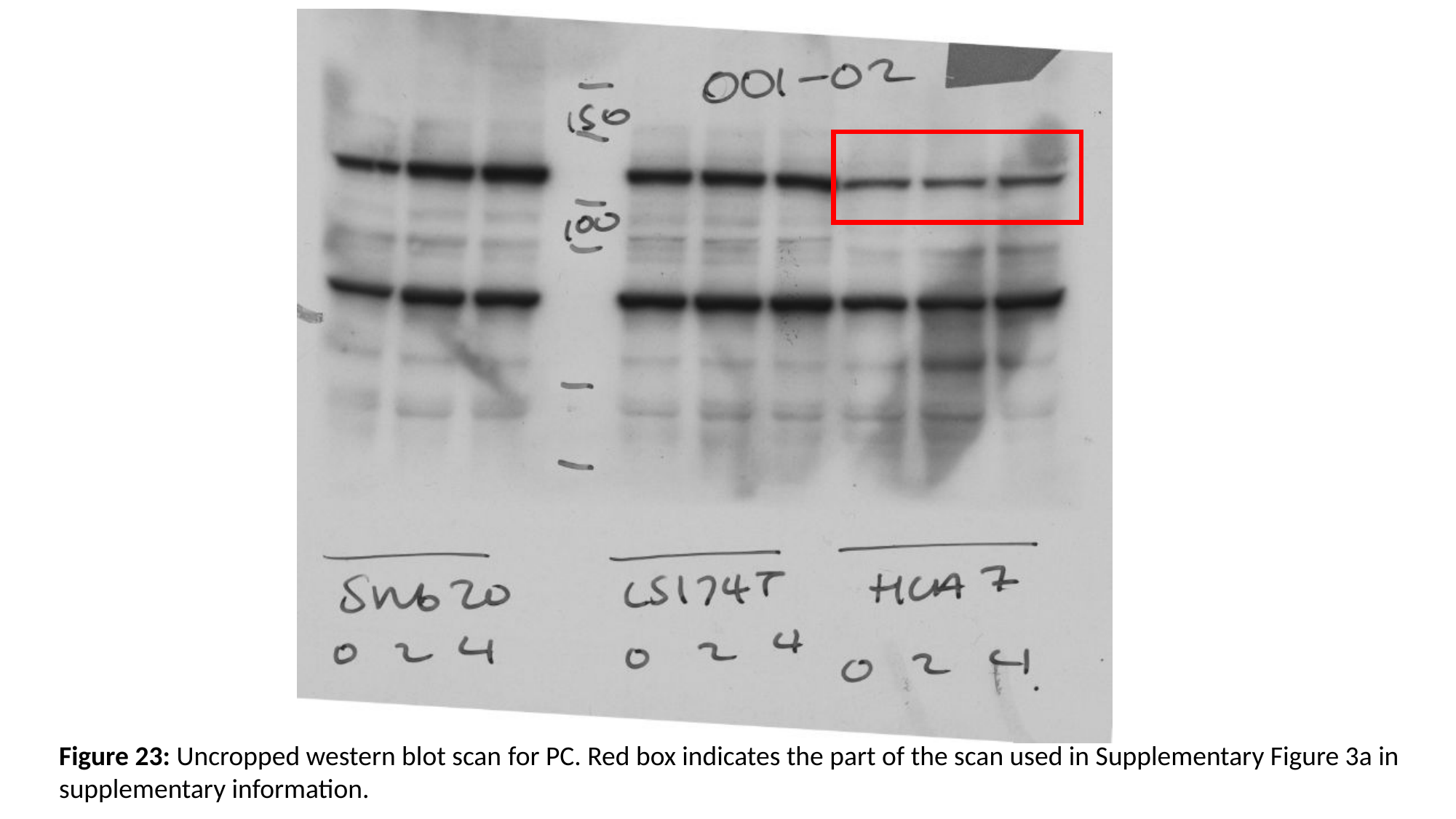

Figure 23: Uncropped western blot scan for PC. Red box indicates the part of the scan used in Supplementary Figure 3a in supplementary information.

## Slide 24
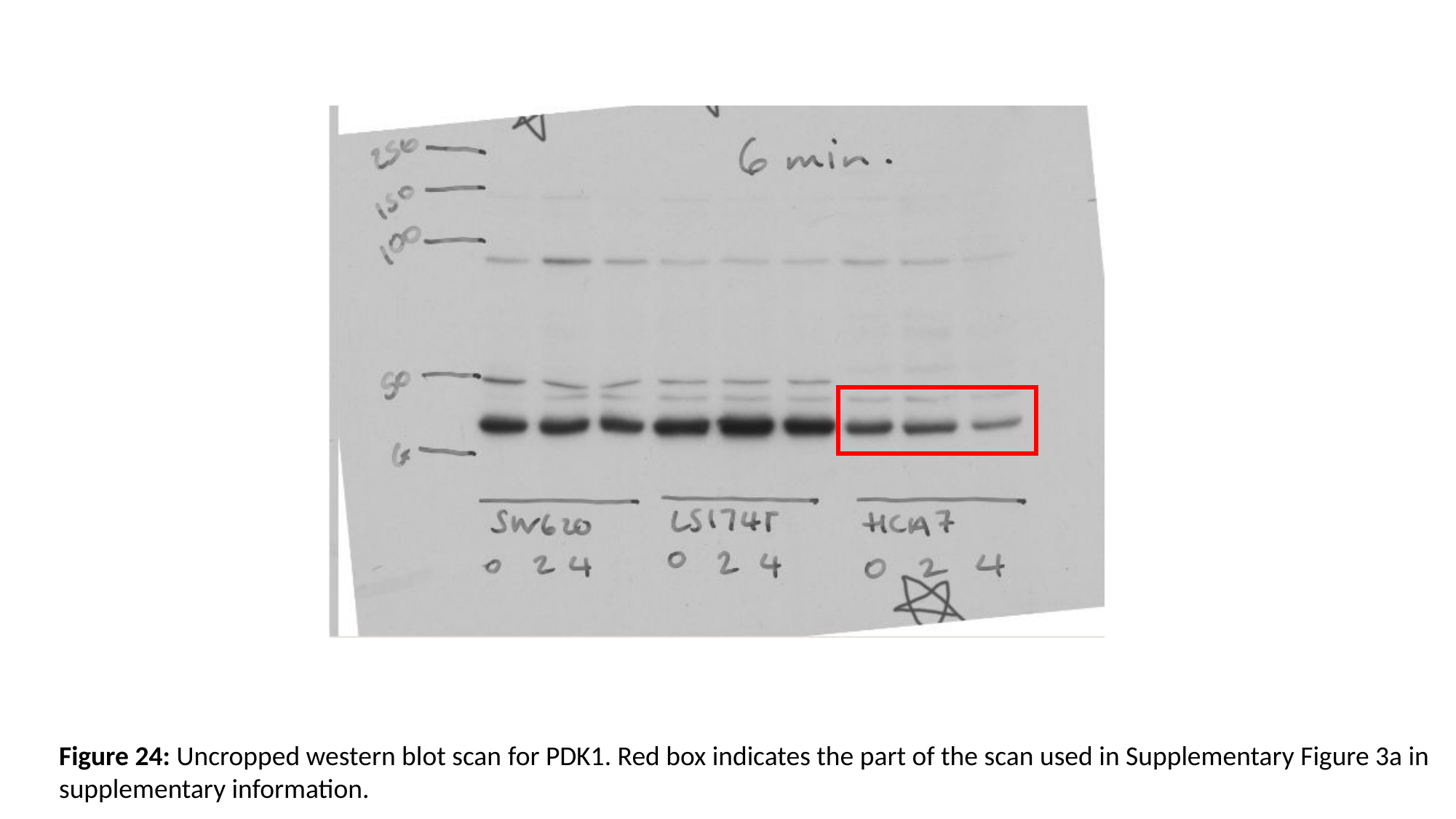

Figure 24: Uncropped western blot scan for PDK1. Red box indicates the part of the scan used in Supplementary Figure 3a in supplementary information.

## Slide 25
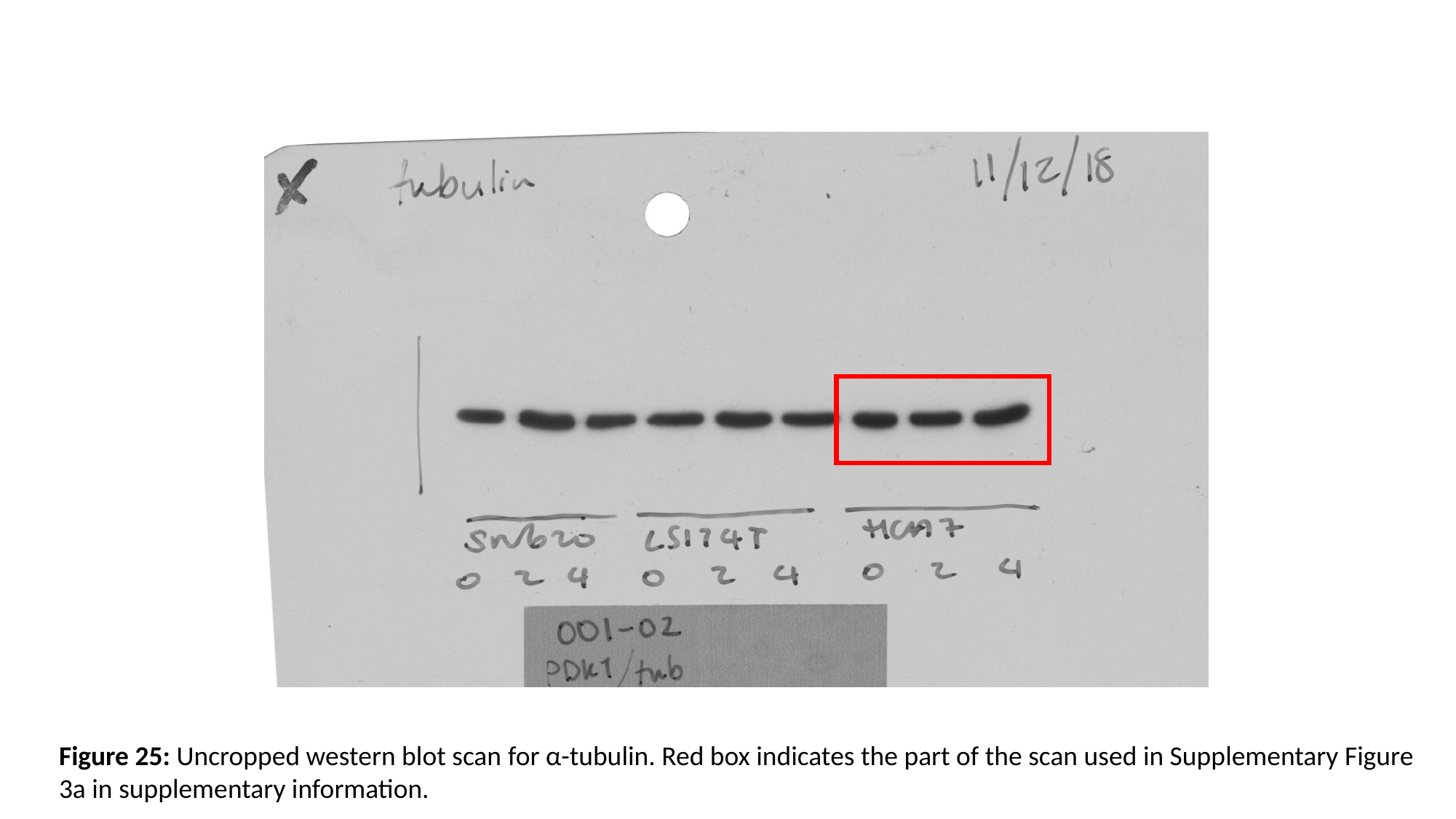

Figure 25: Uncropped western blot scan for α-tubulin. Red box indicates the part of the scan used in Supplementary Figure 3a in supplementary information.

## Slide 26
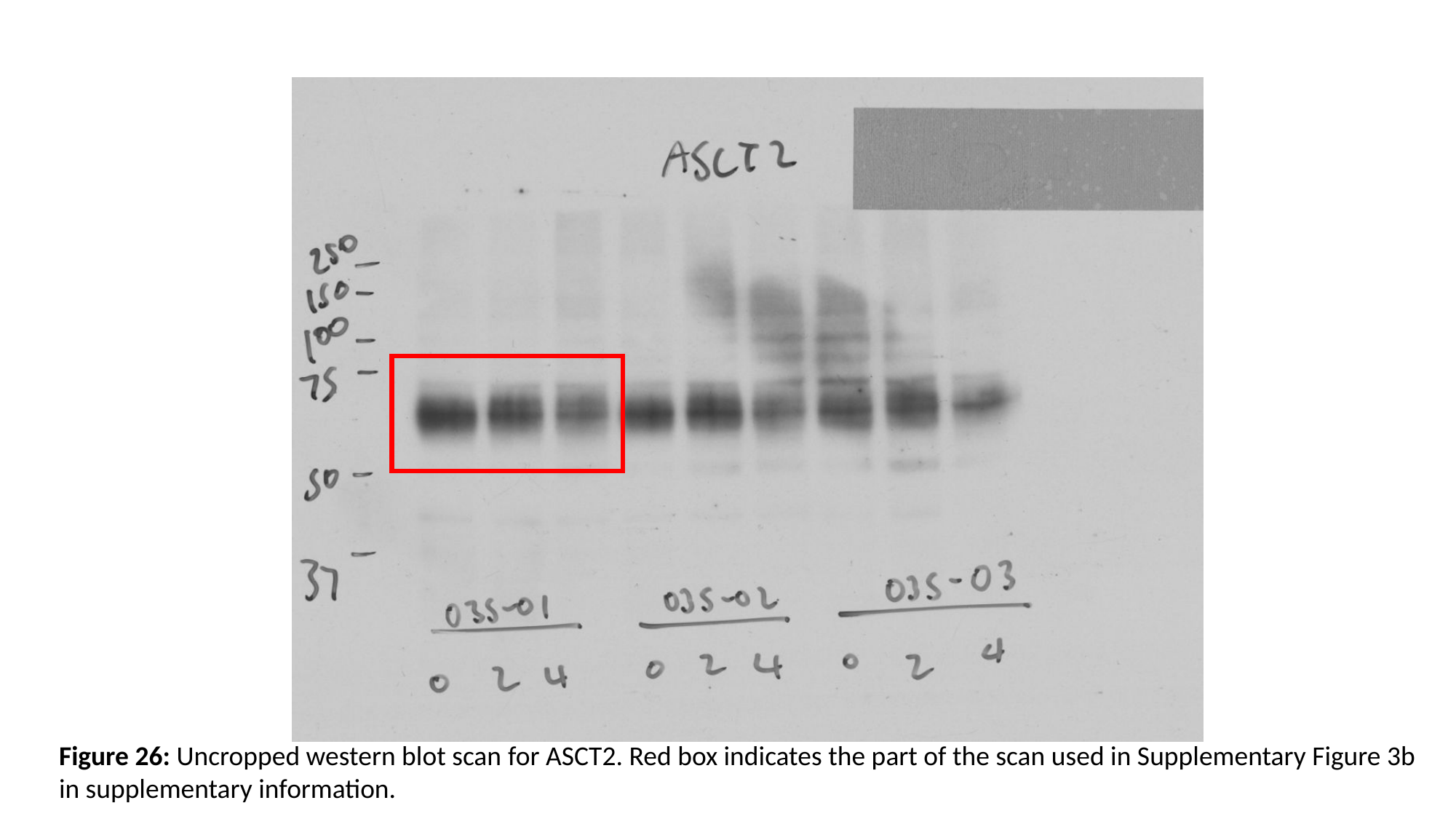

Figure 26: Uncropped western blot scan for ASCT2. Red box indicates the part of the scan used in Supplementary Figure 3b in supplementary information.

## Slide 27
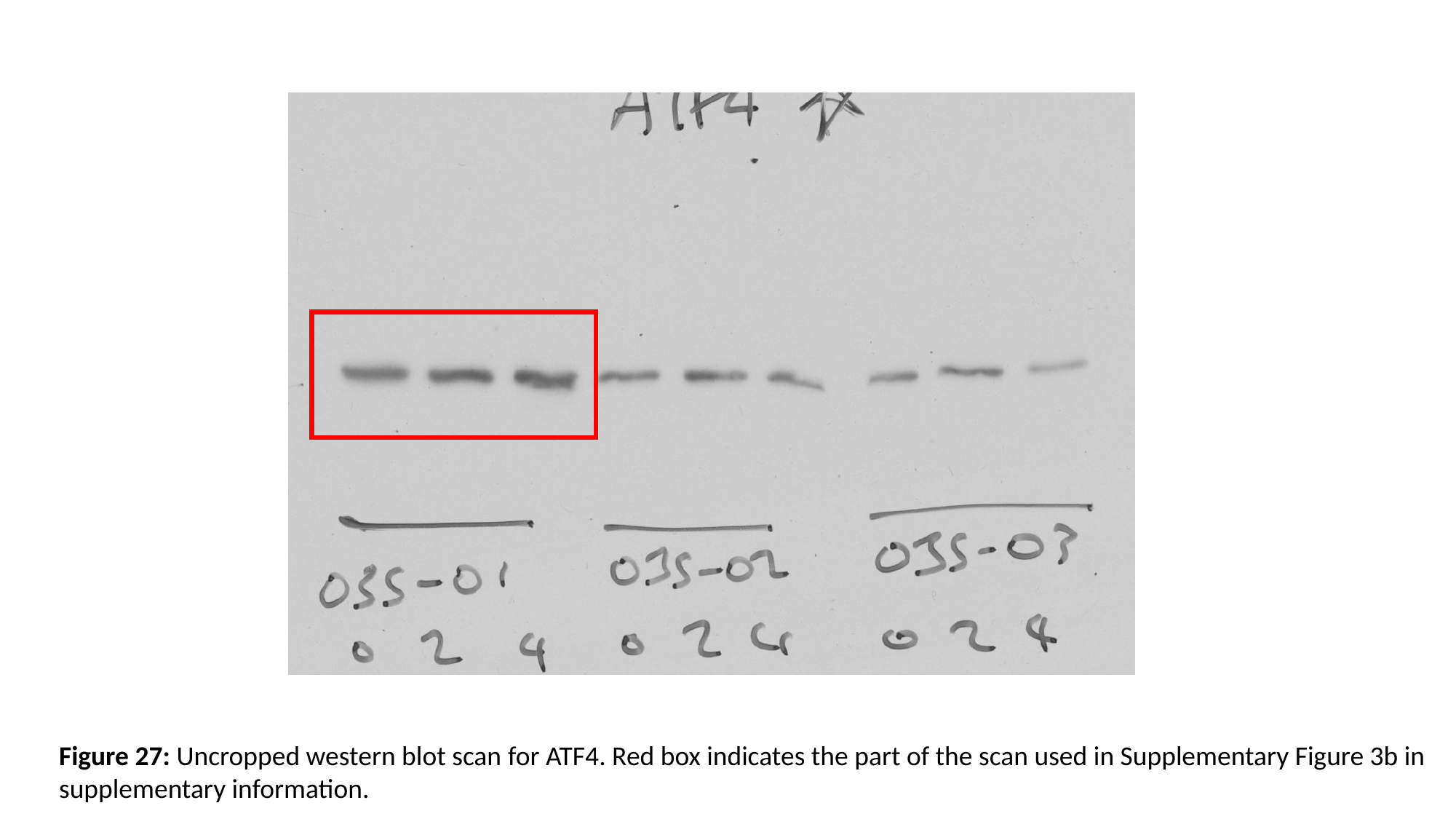

Figure 27: Uncropped western blot scan for ATF4. Red box indicates the part of the scan used in Supplementary Figure 3b in supplementary information.

## Slide 28
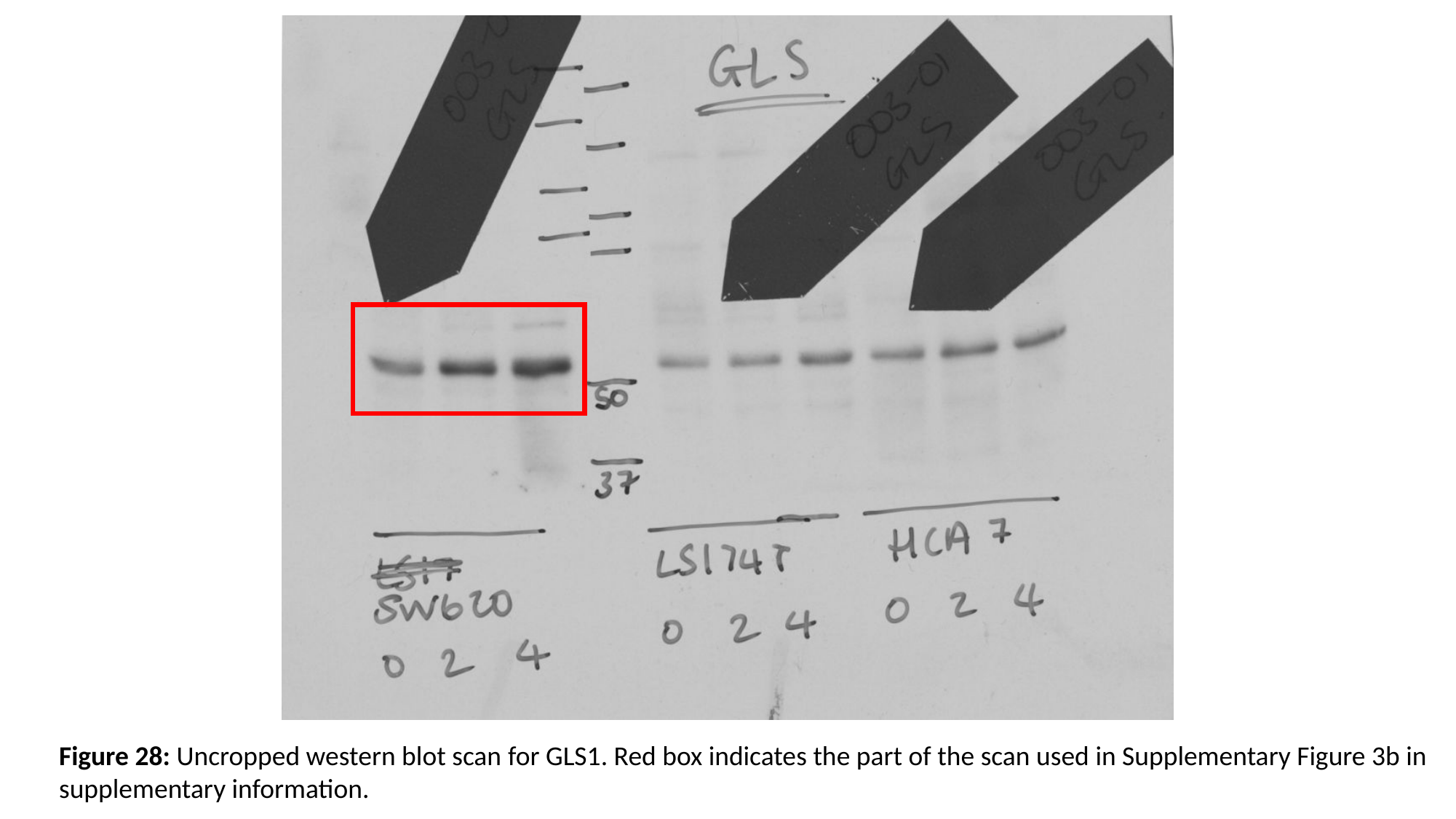

Figure 28: Uncropped western blot scan for GLS1. Red box indicates the part of the scan used in Supplementary Figure 3b in supplementary information.

## Slide 29
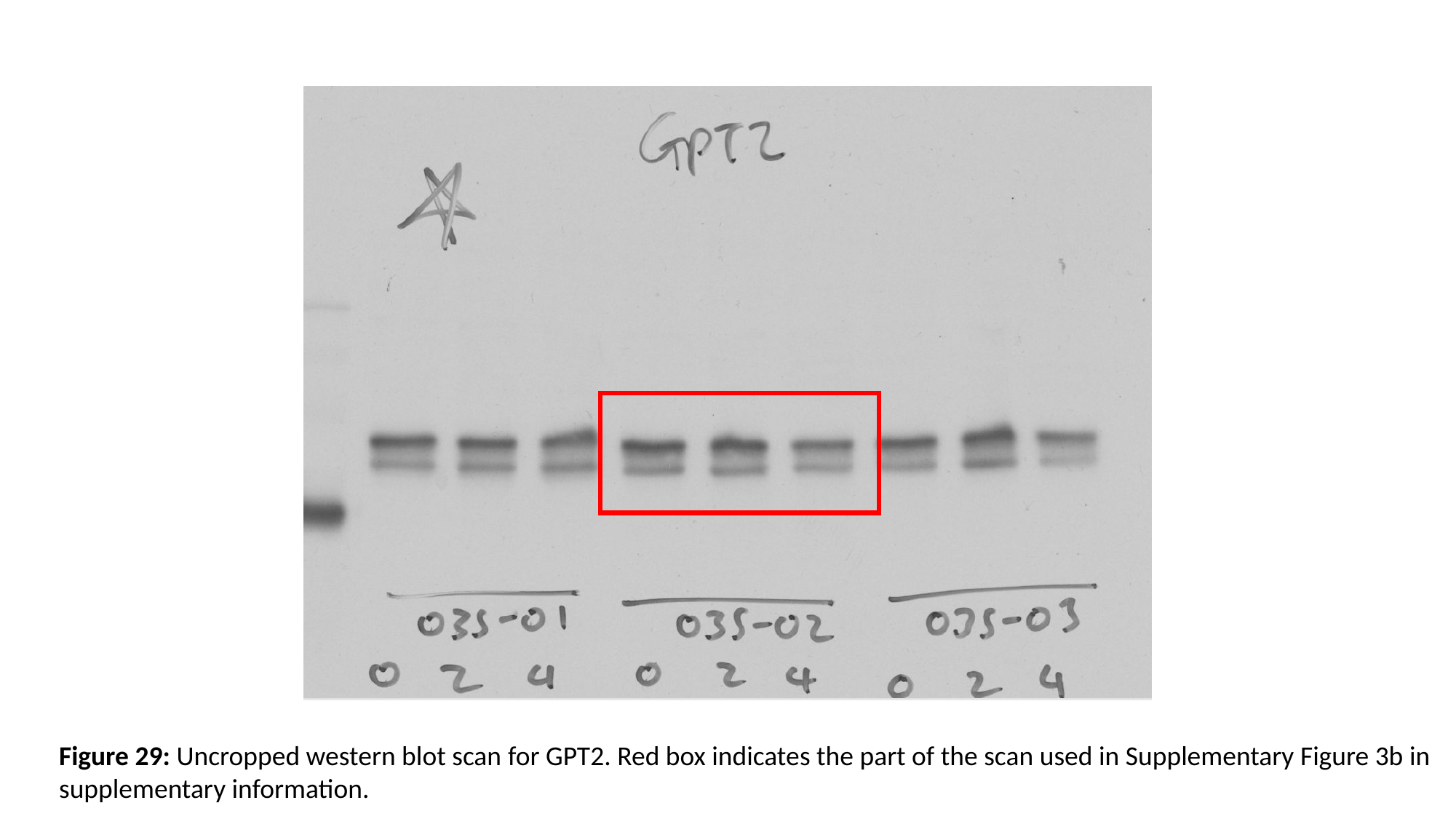

Figure 29: Uncropped western blot scan for GPT2. Red box indicates the part of the scan used in Supplementary Figure 3b in supplementary information.

## Slide 30
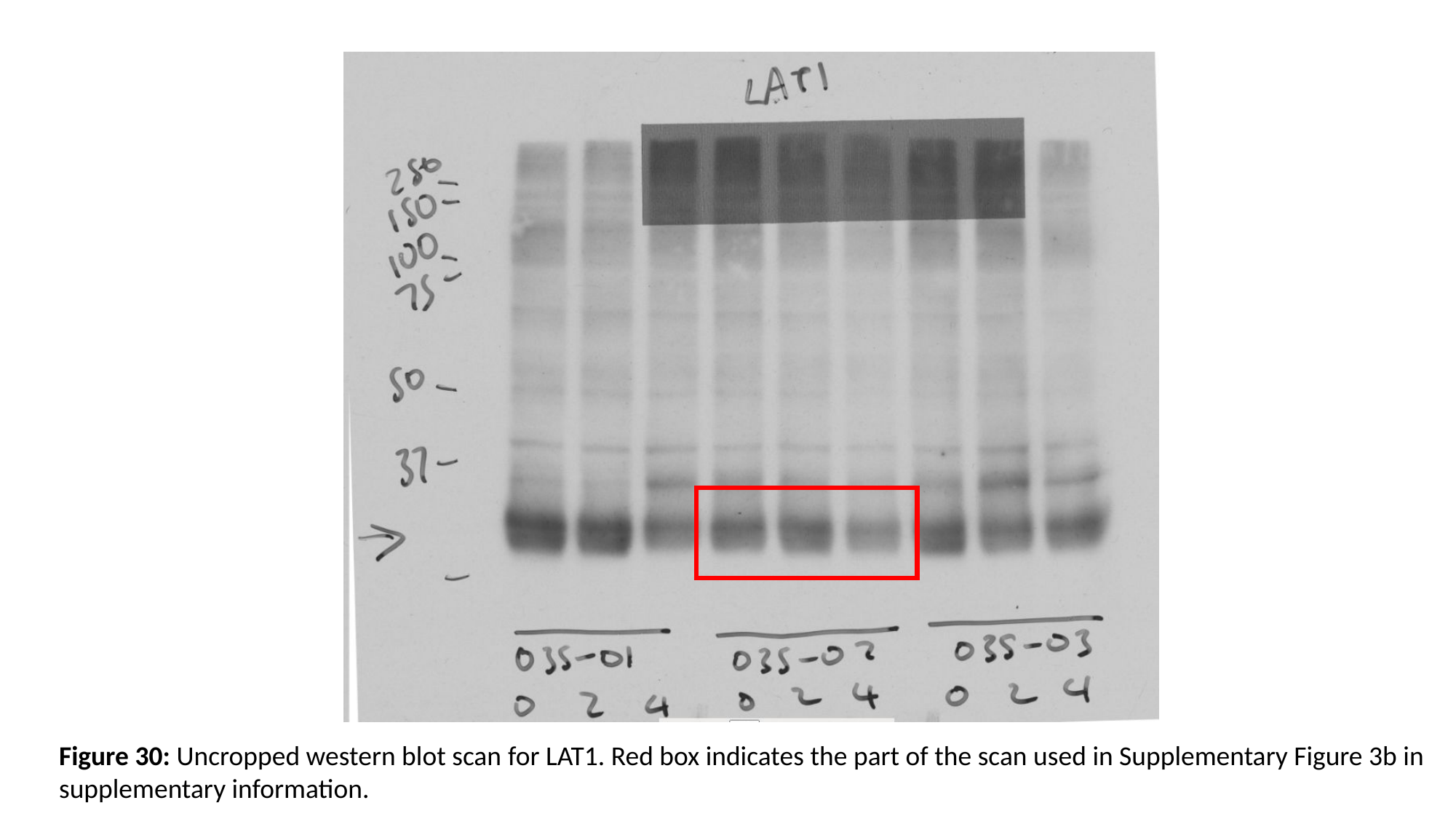

Figure 30: Uncropped western blot scan for LAT1. Red box indicates the part of the scan used in Supplementary Figure 3b in supplementary information.

## Slide 31
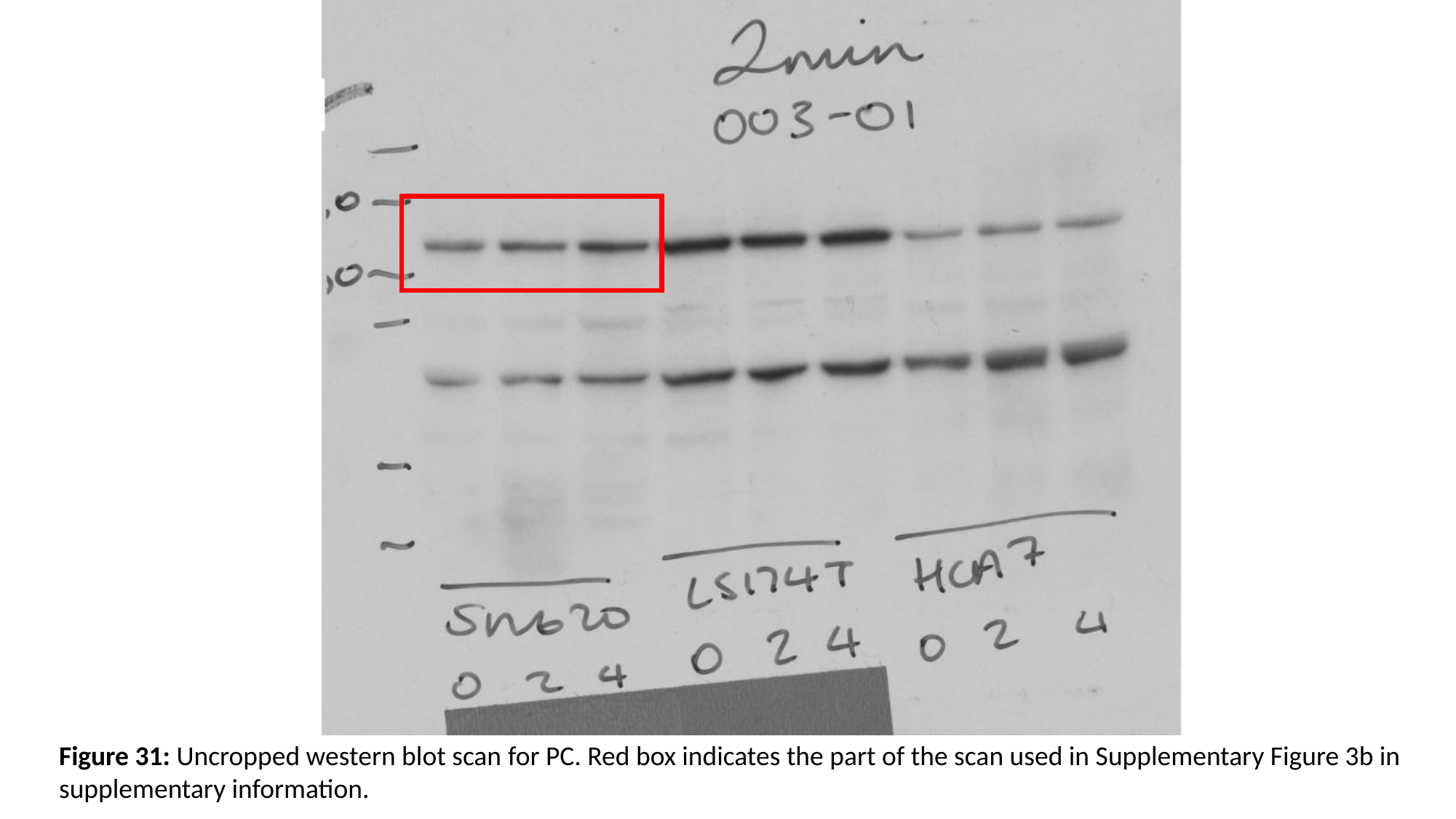

Figure 31: Uncropped western blot scan for PC. Red box indicates the part of the scan used in Supplementary Figure 3b in supplementary information.

## Slide 32
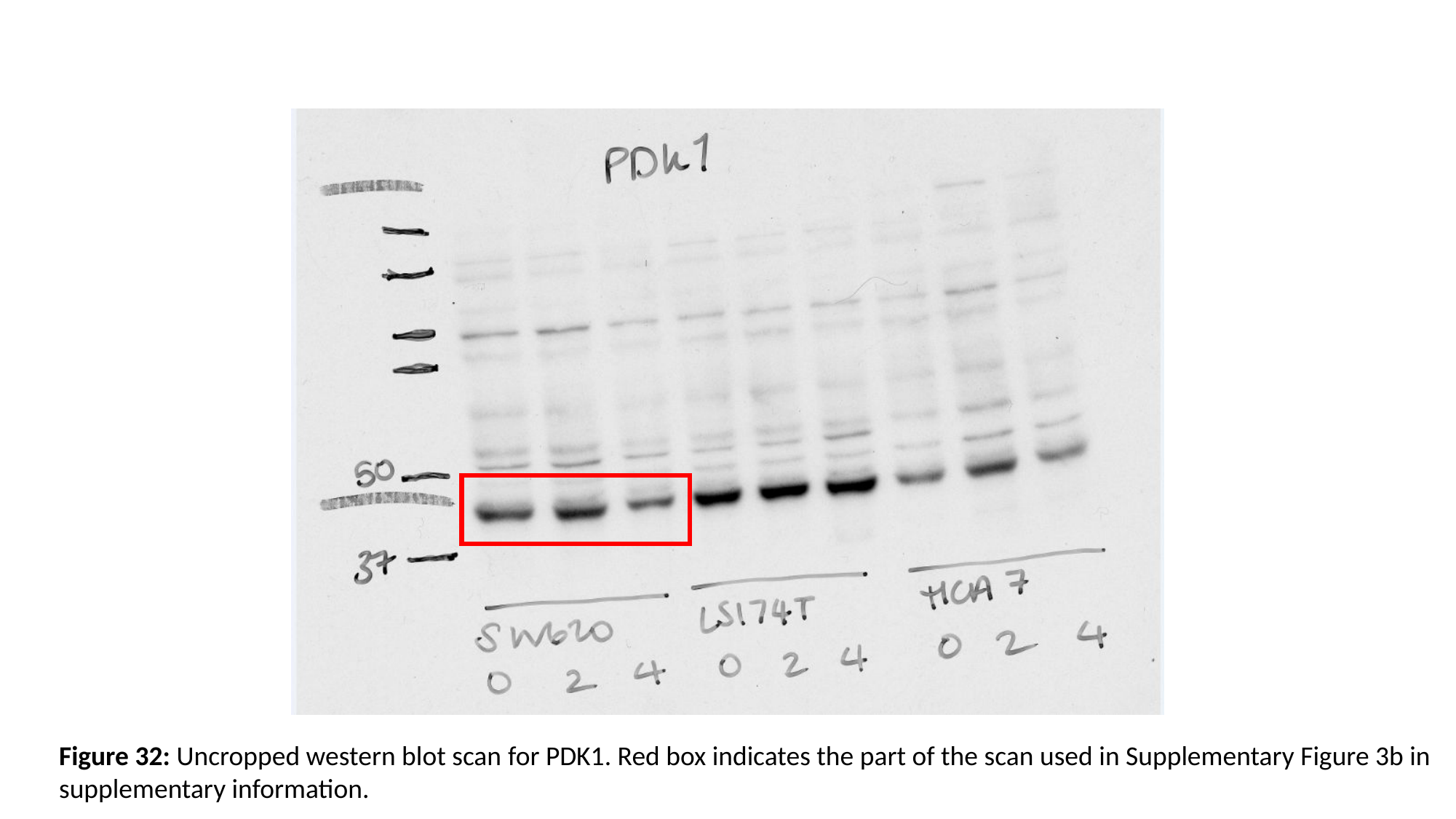

Figure 32: Uncropped western blot scan for PDK1. Red box indicates the part of the scan used in Supplementary Figure 3b in supplementary information.

## Slide 33
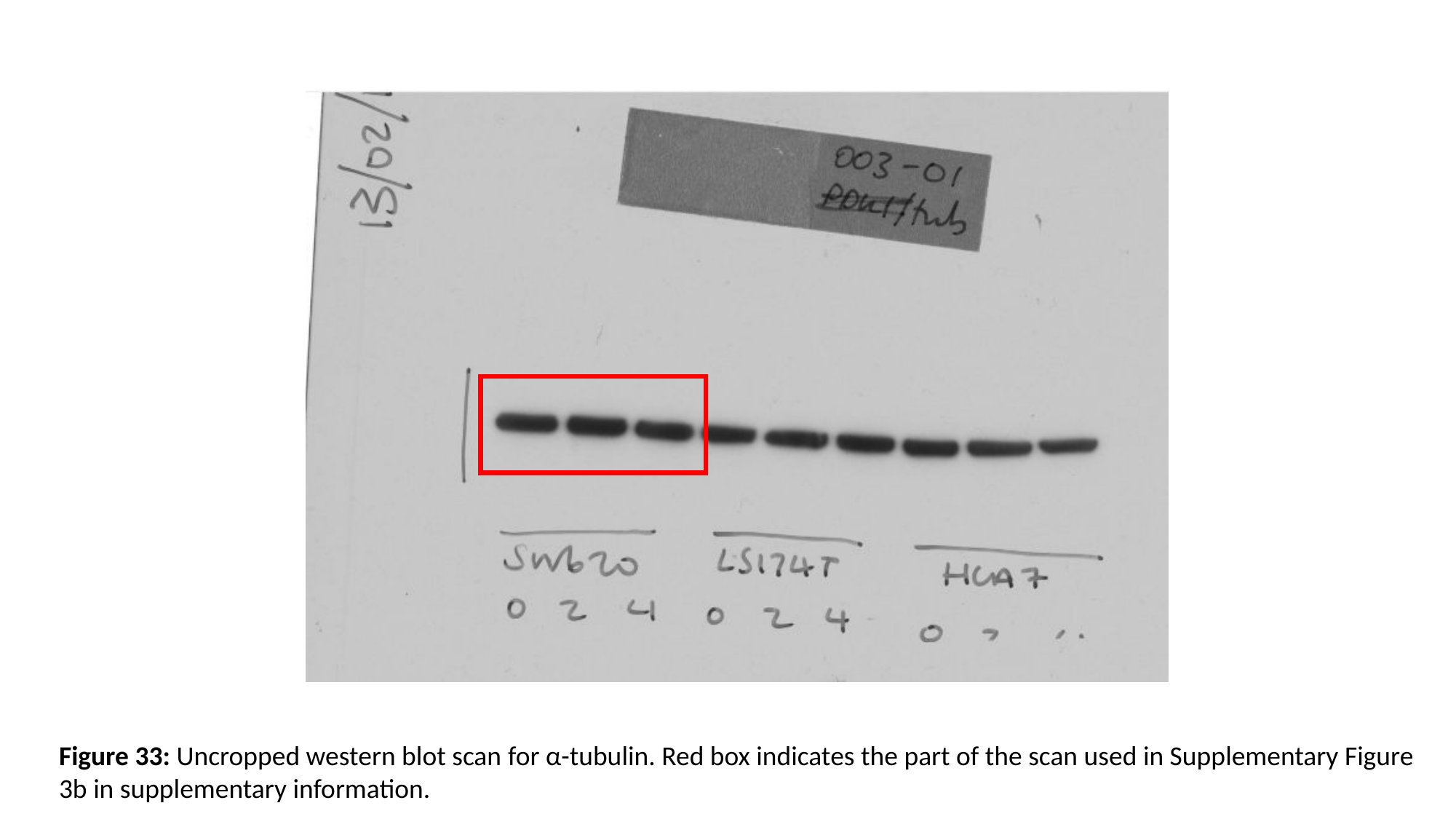

Figure 33: Uncropped western blot scan for α-tubulin. Red box indicates the part of the scan used in Supplementary Figure 3b in supplementary information.

## Slide 34
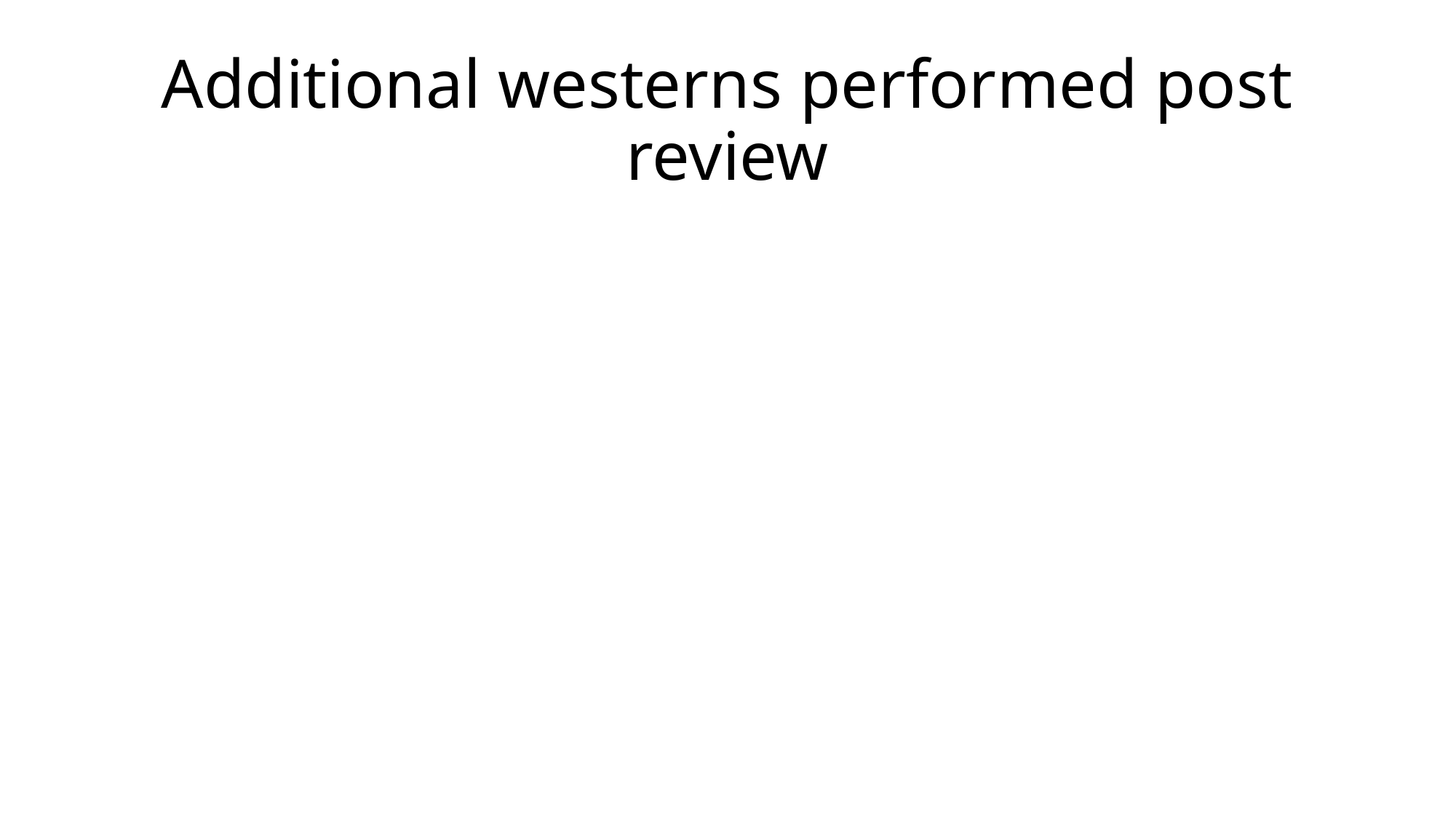

# Additional westerns performed post review

## Slide 35
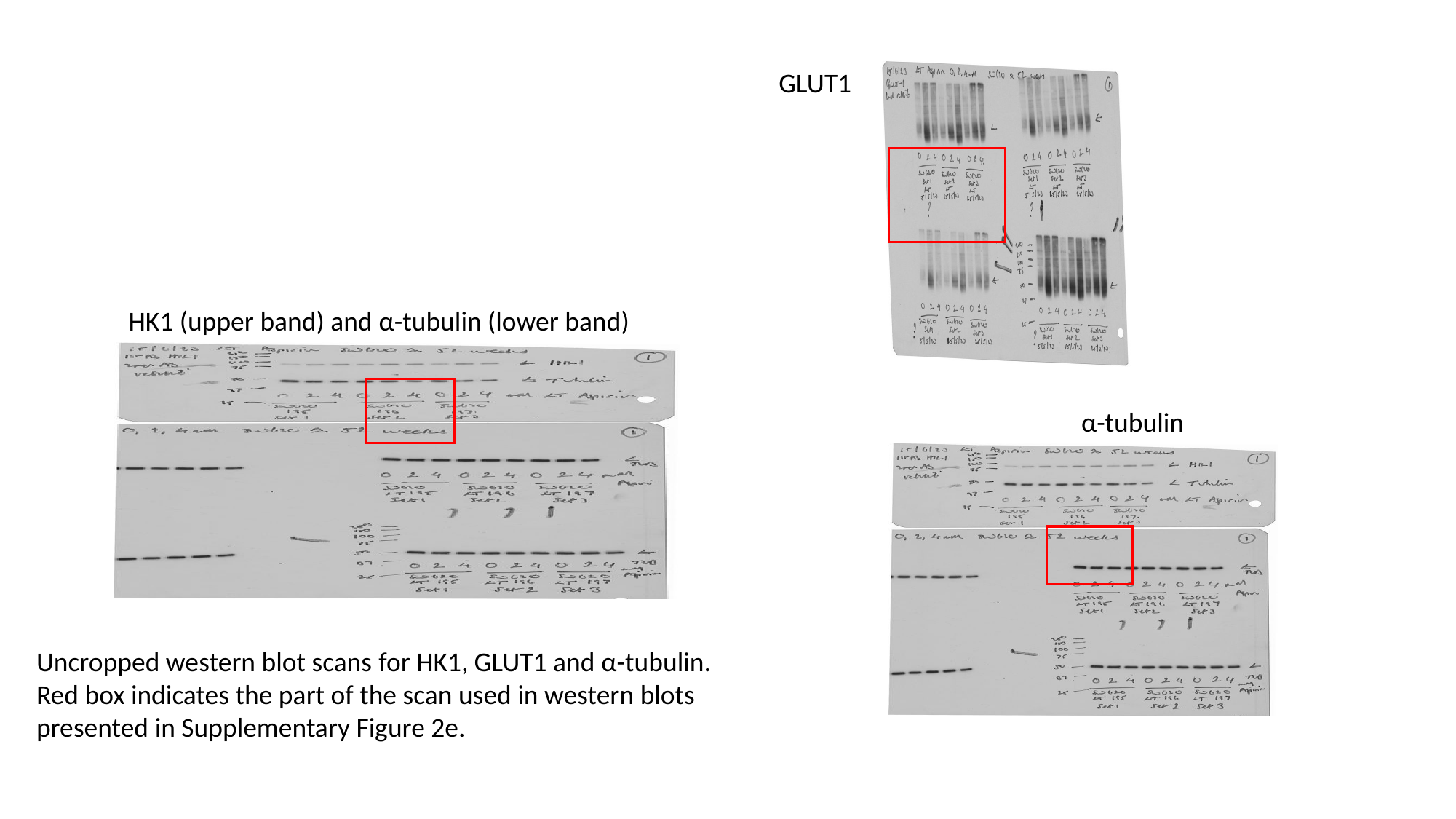

GLUT1
HK1 (upper band) and α-tubulin (lower band)
α-tubulin
Uncropped western blot scans for HK1, GLUT1 and α-tubulin. Red box indicates the part of the scan used in western blots presented in Supplementary Figure 2e.
